# Ring-closing metathesis of prochiral oxaenediynes to racemic 4-alkenyl-2-alkynyl-3,6-dihydro-2*H*-pyrans

**DOI:** 10.3762/bjoc.16.226

**Published:** 2020-11-13

**Authors:** Viola Kolaříková, Markéta Rybáčková, Martin Svoboda, Jaroslav Kvíčala

**Affiliations:** 1 Department of Organic Chemistry, University of Chemistry and Technology, Prague, Technická 5, 166 28 Prague 6, Czech Republic; 2 Institute of Organic Chemistry and Biochemistry, Academy of Sciences of the Czech Republic, v.v.i., Flemingovo nám. 2, 166 10 Prague 6, Czech Republic

**Keywords:** Diels–Alder reaction, enediyne, enyne metathesis, ring-closing metathesis, ruthenium precatalyst

## Abstract

The prochiral 4-(allyloxy)hepta-1,6-diynes, optionally modified in the positions 1 and 7 with an alkyl or ester group, undergo a chemoselective ring-closing enyne metathesis yielding racemic 4-alkenyl-2-alkynyl-3,6-dihydro-2*H*-pyrans. Among the catalysts tested, Grubbs 1st generation precatalyst in the presence of ethene (Mori conditions) gave superior results compared to the more stable Grubbs or Hoveyda–Grubbs 2nd generation precatalysts. This is probably caused by a suppression of the subsequent side-reactions of the enyne metathesis product with ethene. On the other hand, the 2nd generation precatalysts gave better yields in the absence of ethene. The metathesis products, containing both a triple bond and a conjugated system, can be successfully orthogonally modified. For example, the metathesis product of 5-(allyloxy)nona-2,7-diyne reacted chemo- and stereoselectively in a Diels–Alder reaction with *N*-phenylmaleimide affording the tricyclic products as a mixture of two separable diastereoisomers, the configuration of which was estimated by DFT computations. The reported enediyne metathesis paves the way to the enantioselective enyne metathesis yielding chiral building blocks for compounds with potential biological activity, e.g., norsalvinorin or cacospongionolide B.

## Introduction

Among the plethora of metathetic reactions of unsaturated compounds, e.g., alkene and enyne cross metathesis (CM [1] and EYCM [2–4]), alkene and enyne ring-closing metathesis (RCM and RCEYM) [5–6], ring-opening metathetic polymerization (ROMP) [7], etc., the CM and RCM are the most popular in organic synthesis [8]. Furthermore, the reactions of substrates containing three or more multiple bonds attracted special attention due to the applications in tandem metathesis processes [9–10] or stereodiscriminating enantioselective ring-closing metathetic reactions [11–13]. Apart from metathesis, enynes are also interesting substrates for other catalytic reactions, e.g., for the Pauson–Khand reaction [14].

In the enantioselective RCM, prochiral trienes have been most often employed, leading to chiral cycloalkenes. The Schrock molybdenum precatalysts [15–17] proved to be more effective than the Grubbs or Collins ruthenium precatalysts [18–20] in the enantioselective RCM, however, the high air and moisture sensitivity makes their use less practical.

**Scheme 1:**
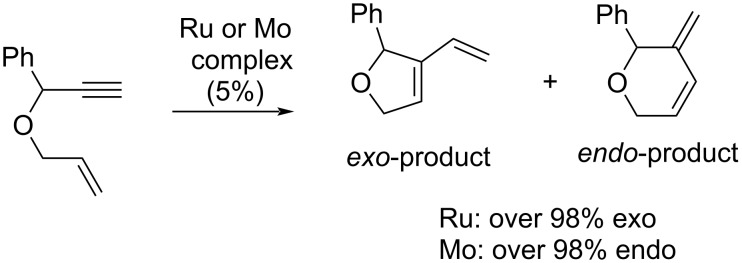
RCEYM with Ru and Mo catalysts.

The choice of the catalyst is one of the key elements in both cross and ring-closing enyne metathesis and the other is the substrate structure. Both of these factors determine the metathesis mechanism: i.e., whether a double (ene-then-yne mechanism) or a triple (yne-then-ene mechanism) bond first enters the initiation step of the precatalyst activation. The group 6 metal-based precatalysts prefer the latter mechanism and yield the *endo*-products, while Ru precatalysts enable both mechanisms and generally follow the ene-then-yne mechanism for substrates with a sterically unhindered double bond, yielding the *exo*-products (Scheme 1) [21–26].

**Scheme 2:**
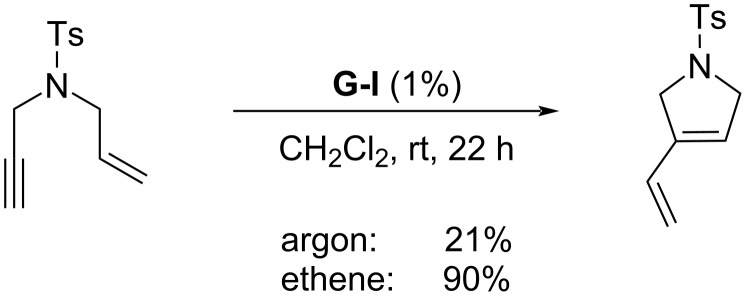
Beneficial effect of ethene atmosphere.

The presence of ethene (Mori conditions) is often beneficial in RCEYM reactions, giving significantly better yields (Scheme 2). This effect was explained to be caused not by an improved activation of the ruthenium precatalyst, but either by the participation in the second metathesis cycle releasing the diene product and returning methyleneruthenium complex to the catalytic cycle [22], or by preventing the catalytic intermediate to undergo subsequent metathesis reactions leading to catalyst deactivation [23]. In both cases, the effect of ethene was especially productive for the Grubbs 1st generation precatalyst and is typically applied for terminal alkynes or alkynes with little steric hindrance of the triple bond.

In order to obtain enantiomerically pure compounds by enyne metathesis, a diastereoselective approach starting from chiral substrates is by far the most common. It was frequently used in the construction of chiral compounds, e.g., the mansamine framework [27], anatoxin [28] or sulfoximines [29].

**Scheme 3:**
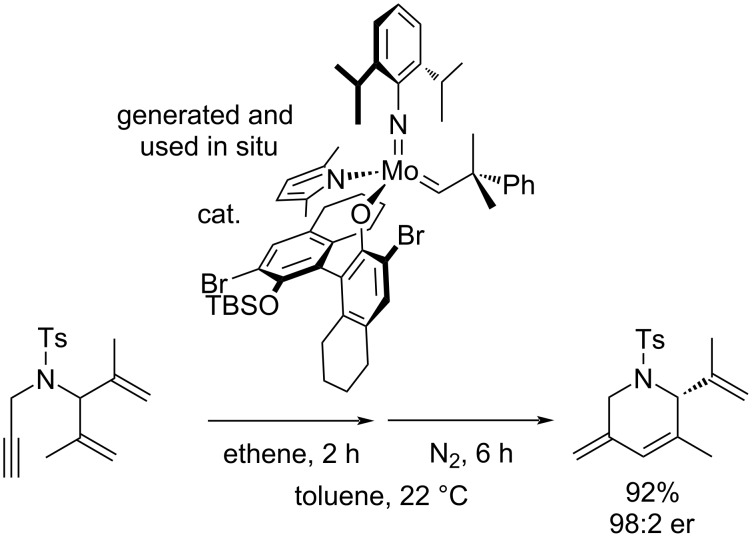
Enantioselective dienyne metathesis [21].

A significantly more demanding strategy uses prochiral substrates and chiral metathesis precatalysts. Despite many examples of enantioselective RCM, only two examples of an enantioselective RCEYM have been reported (both featuring a dienyne substrate and a Schrock complex) [21,30], no enantioselective enediyne metathesis or enantioselective RCEYM reactions catalyzed by ruthenium complexes are known (Scheme 3).

**Scheme 4:**
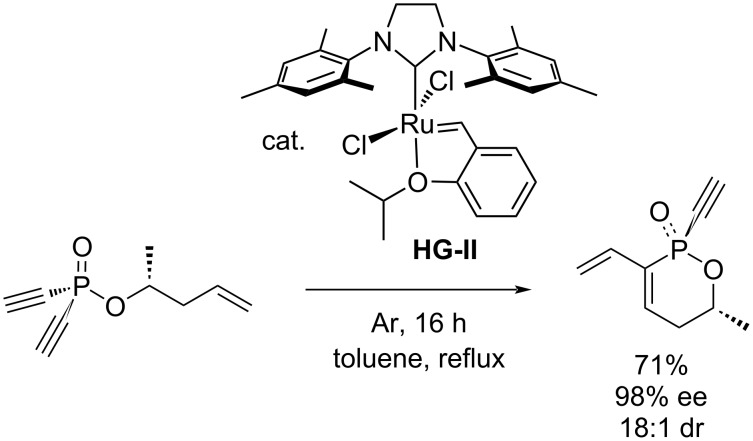
Diastereoselective endiyne metathesis [31].

The only RCEYM of a system containing two triple and one double bonds has been described by Gouverneur et al., who synthesized a series of dihydrooxaphosphinines by the diastereoselective metathesis of alkenyl dialkynylphosphinates (Scheme 4) [31].

Thus, the possibility to perform a desymmetrizing RCEYM of oxaenediynes, which should lead to chiral compounds bearing both alkyne and conjugated diene systems, seemed highly appealing to us. Before addressing the enantioselective RCEYM, we report in this article the scope and limitations of the racemic metathesis with the emphasis on catalysts used, optional application of Mori conditions, and substitution in both the alkyne and the allyloxy part of the enediynes studied.

## Results and Discussion

### Choice and synthesis of starting substrates

**Figure 1:**
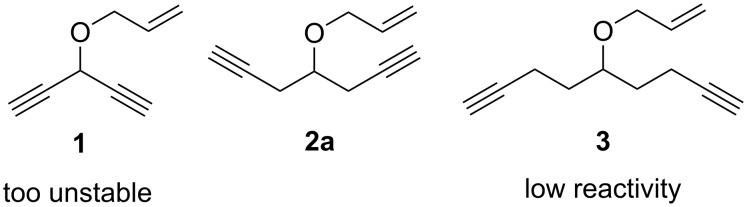
Oxaenediynes considered for the study of desymmetrizing RCEYM.

In our research of desymmetrizing RCEYM of substrates bearing two triple and one double bonds, we decided to study prochiral oxaenediynes due to their potential synthetic availability. We originally considered the three possible structures **1**–**3** (Figure 1). However, in preliminary experiments, oxaenediyne **1** proved to be too unstable and prone to polymerization. On the other hand, the reactivity of oxaenediyne **3** was too sluggish. Hence, 4-(allyloxy)hepta-1,6-diyne (**2a**) and its derivatives became the framework of choice.

**Scheme 5:**
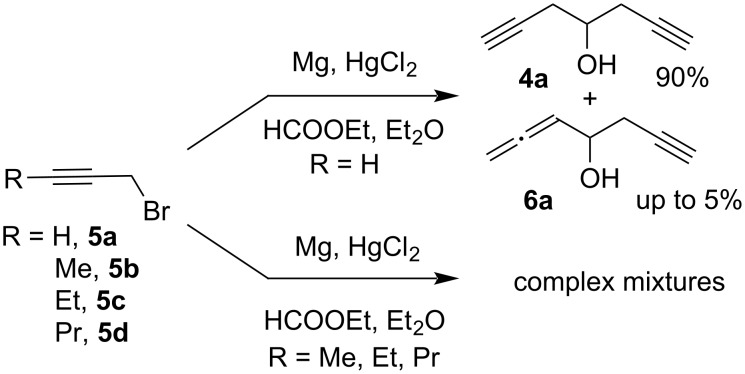
Synthesis of hepta-1,6-diyn-4-ol (**4a**).

The key starting compound for the synthesis of oxaenediyne **2a** and its derivatives was hepta-1,6-diyn-4-ol (**4a**). The compound was prepared according to a published procedure [32] by the reaction of ethyl formate with propargylmagnesium bromide, generated from propargyl bromide (**5a**), magnesium and a catalytic amount of HgCl_2_ to suppress the formation of allenylmagnesium bromide (Scheme 5). The target diynol **4a** was obtained in a 90% yield contaminated with up to 5% of hepta-1,2-dien-6-yn-4-ol (**6a**), which could not be separated at this stage; however, derivatives of this compound could be conveniently removed by column chromatography in the subsequent steps. Unfortunately, the analogous reactions of but-2-ynyl bromide (**5b**), pent-2-ynyl bromide (**5c**), and hex-2-ynyl bromide (**5d**) gave complex mixtures with significant admixtures of allenic products (Scheme 5).

**Scheme 6:**
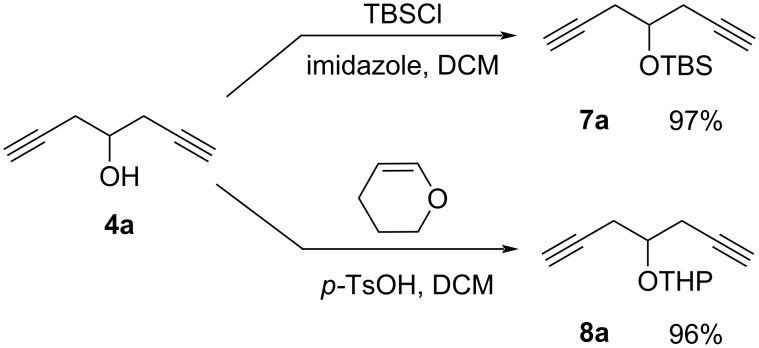
Protection of hepta-1,6-diyn-4-ol (**4a**).

An alternative synthetic pathway to diynols **4b**–**d** was hence developed consisting of the protection of the hydroxy group in diynol **4a** as *tert*-butyldiphenylsilyl ether according to reference [32] or with a tetrahydropyranyl group. In both cases, we obtained the target products **7a** and **8a** in nearly quantitative yields. Interestingly, a reaction time over 1 h in the THP protection led to the formation of degradation products and a significantly decreased yield (Scheme 6).

**Scheme 7:**
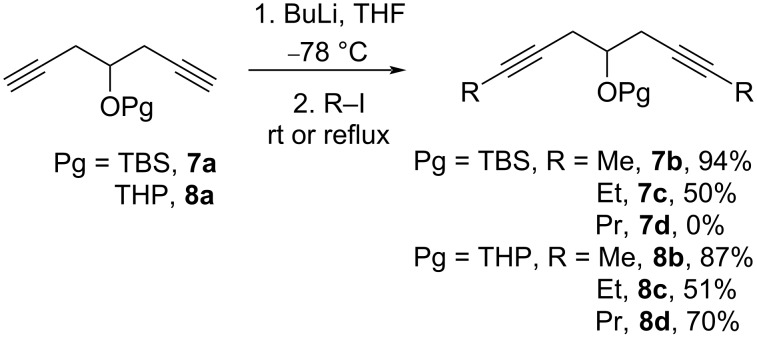
Alkylation of the protected diynols **7a** and **8a**.

Then, the TBS or THP-protected alcohols **7a** and **8a** were alkylated in an analogous manner to a published procedure [33]. Thus, the compounds **7a** and **8a** were treated with an excess of BuLi (3.5–4 equiv) in THF at −78 °C, followed by the addition of the respective alkyl iodides (4–5 equiv) and stirring the mixture either at rt (for R = Me) or at reflux (for R = Et, Pr). For longer alkyl-chain iodides, a complete disubstitution was difficult to achieve and only moderate yields of the products were obtained. The synthesis of oxadiynol **7d** starting from the silyl-protected substrate failed (Scheme 7).

**Scheme 8:**
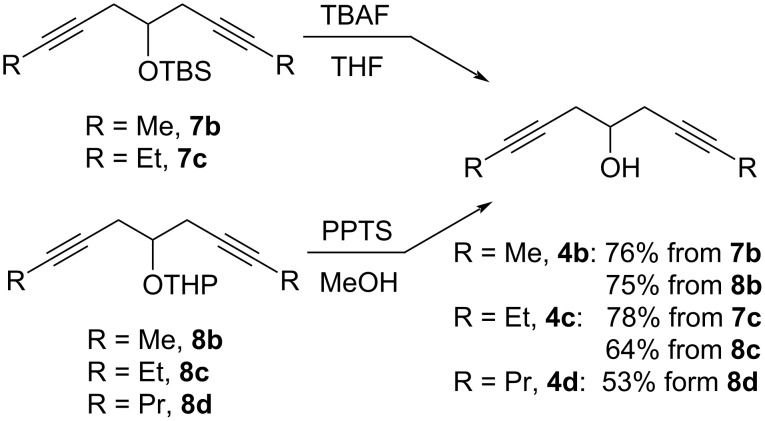
Deprotection of protected diynols **7b**, **7c** and **8b**–**d**.

Next, the alkylated diynols **7b**, **7c**, and **8b**–**d** were deprotected using standard methodologies affording the products in moderate to good yields (Scheme 8).

**Scheme 9:**
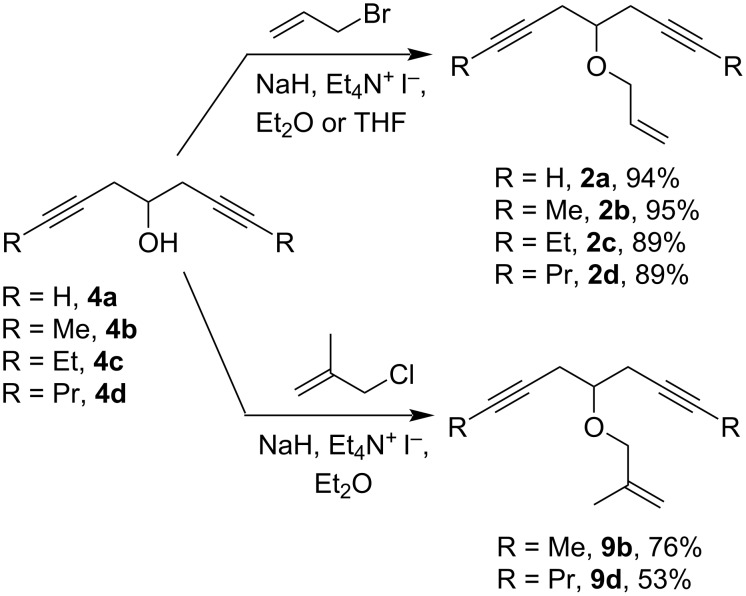
Synthesis of the oxaenediynes **2** and **9** bearing an allyl or a methallyl group.

With the diynols **4a**–**d** in hand, we were able to synthesize a small library of allylated oxaenediynes **2** by the alkylation of the diynols **4** with allyl bromide, NaH, and a catalytic amount of tetraethylammonium iodide. The analogous synthesis of selected methallylated oxaenediynes **9** required longer reaction times and a higher excess of methallyl chloride (3 equiv). Unreacted diynols **4** could be recovered by column chromatography (Scheme 9).

**Scheme 10:**
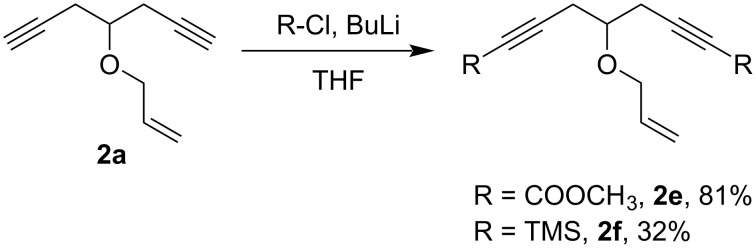
Synthesis of oxaenediynes **2e** and **2f** bearing ester or silyl groups.

With the aim to enable the further functionalization of the products of the oxaenediyne metathesis, we also synthesized substrates modified at the terminal alkyne positions with silyl or ester groups. Because these substrates were inaccessible by the above described methods, we obtained the compounds by substituting the terminal acetylenic hydrogens in the parent oxaenediyne **2a** by treatment with butyllithium and methyl chloroformate or chlorotrimethylsilane (Scheme 10). The target disubstituted products **2e** and **2f** were formed in acceptable to good yields, with the complete disubstitution being again the issue.

**Scheme 11:**
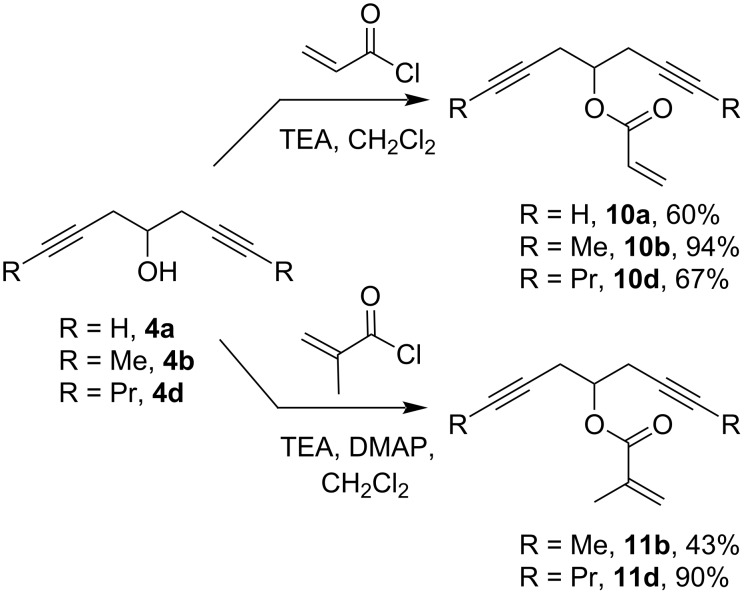
Synthesis of alkadiynyl acrylates **10** and methacrylates **11**.

To evaluate how the electron density on the double bond of the oxaenediyne could influence the RCEYM, we also synthesized oxaenediynes bearing an electron-deficient double bond from enediynols **4** and acryloyl chloride or methacryloyl chloride with triethylamine as a base. The formation of the less reactive methacrylates **11** required longer reaction times and the use of DMAP as an additive (Scheme 11).

### RCEYM of oxaenediynes **2** and **9**–**11**

**Figure 2:**
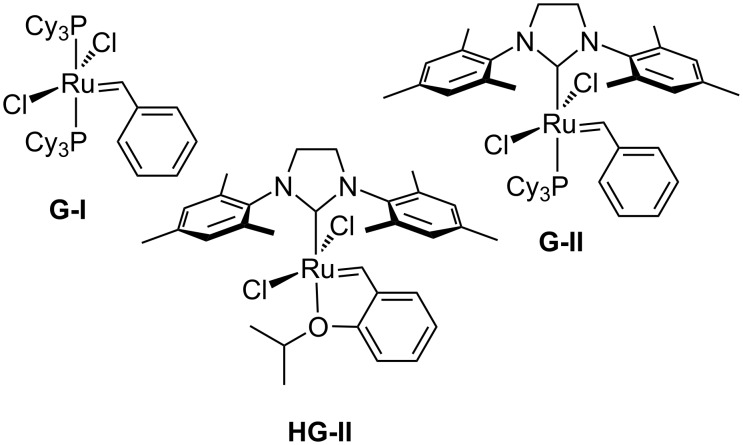
The ruthenium precatalysts employed.

As mentioned in the Introduction, the substrate structure [23–24,34–36], the choice of the precatalyst [24], and the presence of ethene (Mori conditions) [22–23,37] are the major factors influencing the success of the RCEYM. As precatalysts, we chose the Grubbs 1st and 2nd generation (**G-I** and **G-II**), as well as the more stable Hoveyda–Grubbs 2nd generation (**HG-II**) complexes (Figure 2).

Room temperature (25 °C) was chosen as it is common for this type of reactions. In selected cases, we attempted to rise the temperature to improve the conversion but without success. Moreover, the high volatility of most of the substrates resulted in a loss of material unless sealed systems were used. Among the most popular solvents for metathesis, e.g., dichloromethane, 1,2-dichloroethane, and toluene, the former was chosen due to the high volatility of some products, which prevented the isolation from the latter higher boiling solvents. Finally, the **G-I** catalyst loading was tested for selected endiyne metatheses under Mori conditions. While oxaendiyne **2b** bearing Me groups on the triple bonds underwent RCM quantitatively already at 1% catalyst loading, the unsubstituted endiyne **2a** gave negligible conversion under these conditions. On the other hand, 92% yield of the target product was obtained at 5% precatalyst loading in this case. A further increase of the catalyst loading to 10% gave the product in quantitative yield (Table 1). Due to economy and unified conditions, we hence decided to perform all reactions at 5% loading of the precatalyst.

**Table 1:** Optimization of the catalyst loading depending on compound **2** triple bond substitution (NMR yields)^a^.

substituent	cat. loading (%)	yield (%)

H	1	0
H	5	92
H	10	>98
Me	1	>98
Me	5	>98

^a^**G-I** catalyst was used.

**Scheme 12:**
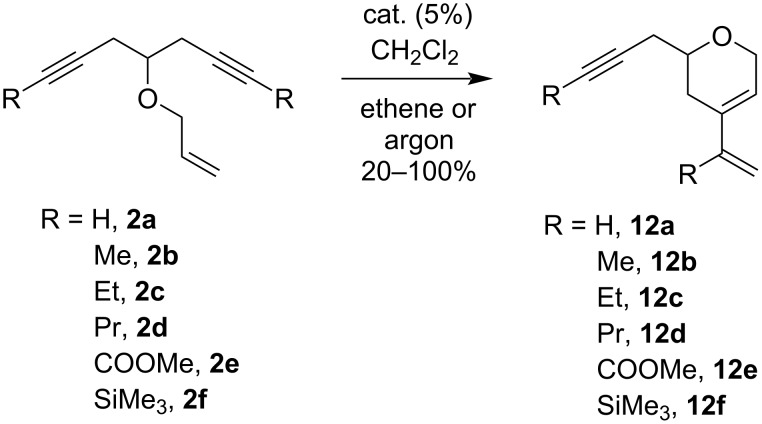
RCEYM of oxaenediynes **2**.

We started our study with substrates **2**, bearing an unsubstituted allyl chain (Scheme 12). The results of the individual experiments and the role of the substrate structure, the precatalyst employed, and atmosphere are collected in Table 2.

**Table 2:** RCEYM of oxaenediynes **2** (NMR yields).

substrate	R	product	catalyst					

			**G-I**		**G-II**		**HG-II**	
			atmosphere		atmosphere		atmosphere	
			ethene	argon	ethene	argon	ethene	argon
			yield (%)		yield (%)		yield (%)	

**2a**	H	**12a**	92^a^	>98	35^b,c^	7	<5^b,c^	<5^b^
**2b**	Me	**12b**	>98^a^	78	28^b,c^	61	40^b,c^	64
**2c**	Et	**12c**	>98^a^	53	29^b,c^	66	27^b,c^	70
**2d**	Pr	**12d**	>98^a^	52	13^b,c^	98	67^b,c^	90
**2e**	CO_2_Me	**12e**	>98^a^	>98	18^b,c^	36	50^b,c^	78
**2f**	SiMe_3_	**12f**	20^c^	0	<5^b,c^	0.5	<5^b,c^	11

^a^Isolated yields: **12a**: 75%, **12b**: 98%, **12c**: 87%,**12d**: 95%, and **12e**: 65%; ^b^contains side-products from CM with ethene (Figure 3); ^c^the yield was estimated from the inseparable product mixture by careful analysis of the vinylic area in the NMR spectra.

In the reactions catalyzed with **G-I**, the ethene atmosphere strongly promoted the reaction and suppressed a number of unwanted processes as described in references [22–23]. Under these conditions a full or nearly complete conversion was observed with the exception of the silylated oxaenediyne **2f** as was already reported for analogous published substrates [37]. Quite surprisingly, the necessity of a steric hindrance at the propargylic position in the RCEYM forming five-membered rings, as described in reference [23], was not observed in our cases. The absence of ethene resulted in a significant decrease of the yield, which gradually decreased from R = H (**2a**) to R = Pr (**2d**). Further, the reaction was not sensitive to the presence of electron-accepting groups (R = COOMe, **2e**) at the triple bonds. Again, substrate **2f** containing the bulkier electron-donating silyl groups gave only low yields of the product. With the exception of the silylated product **12f**, all substituted dihydropyrans **12a**–**e** were isolated as pure racemates in excellent yields when **G-I** as the precatalyst and an ethene atmosphere were applied in the reaction.

**Figure 3:**
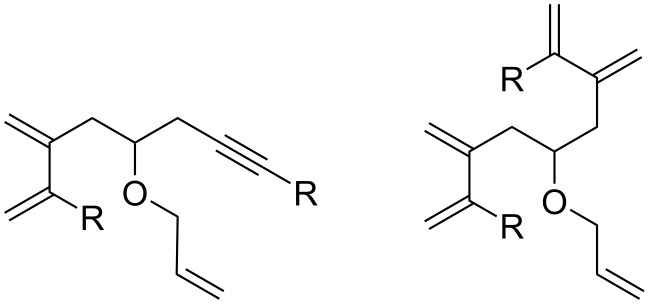
Examples of side products of CM with ethene.

In contrast to **G-I**, the presence of an ethene atmosphere was highly detrimental for the RCEYM catalyzed by the more stable and active **G-II** and **HG-II** precatalysts, as this led to the intermolecular cross-metathesis between the triple bonds of the substrates and ethene (Figure 3) as was previously observed [24]. Thus, complex inseparable mixtures with not fully assignable ^1^H NMR spectra were obtained in all cases.

The results of the RCEYM using the precatalysts **G-II** or **HG-II** in the absence of ethene were significantly better, although they did not reach those using the **G-I** precatalyst and Mori conditions. The yields gradually improved with increasing steric hindrance of the triple bonds from oxaenediyne **2a** (R = H) to **2d** (R = Pr) for both precatalysts **G-II** and **HG-II**. This observation was in agreement with results reported in reference [23], which proved that a ruthenium-containing metathesis intermediate, formed by the alkene cross metathesis in the initiating step followed by a RCEYM, was deactivated by a subsequent reaction with the triple bond of the second enyne molecule. The results of the RCEYM of the ester-modified oxaenediyne **2e** were significantly better for the reaction with the more stable **HG-II** precatalyst, but still inferior to the results achieved with the **G-I** precatalyst. Again, only very low yields were obtained from the RCEYM of the silylated oxaenediyne **2f**.

**Scheme 13:**
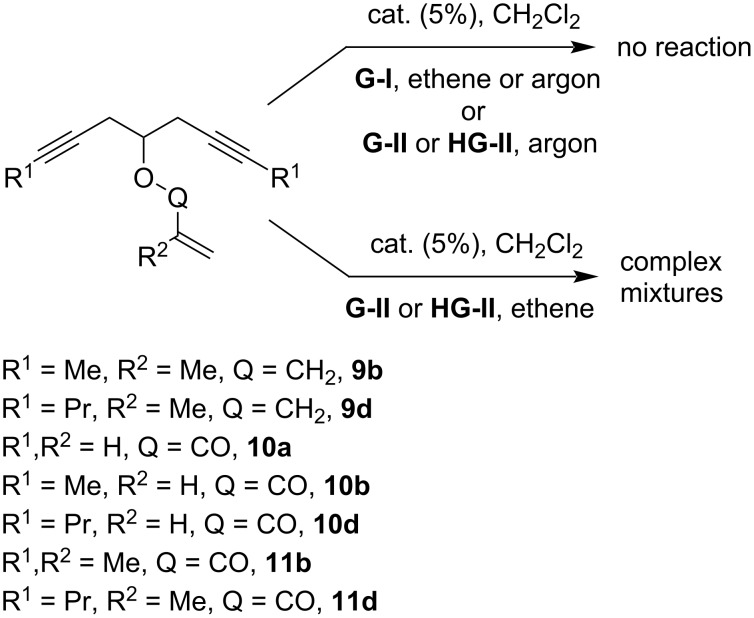
Attempted RCEYM of oxaenediynes **9**, alkadiynyl acrylates **10** and methacrylates **11**.

In sharp contrast to the relatively low sensitivity of the reaction towards the substitution of the triple bonds in the starting oxaenediynes, the modifications of the allyl group resulted in critical loss of reactivity. Thus, the oxaenediynes **9** bearing a methallyl group did not undergo RCEYM under **G-I** catalysis at all, regardless of the atmosphere (ethene or argon) chosen. Similarly, the use of the more active catalysts **G-II** and **HG-II** under an argon atmosphere failed to afford any product. On the other hand, the catalysis with **HG-II** under Mori conditions (ethene atmosphere) gave only inseparable complex mixtures, in which the products of cross metathesis with ethene prevailed and the target products could not be identified (Scheme 13).

To study the role of the electron density of the double bond, we also attempted the RCEYM of alkadiynyl acrylates **10** and alkadiynyl methacrylates **11**. The lower reactivity of the double bond in these substrates led to negative results analogous to the methallyl group containing oxaenediynes **9**, i.e., no reaction was observed for **G-I** catalysis and **G-II** or **HG-II** catalysis under argon, or a complex mixture was obtained for **G-II** or **HG-II** catalysis using Mori conditions (Scheme 13).

### Diels–Alder reaction of RCEYM product **12b**

**Scheme 14:**
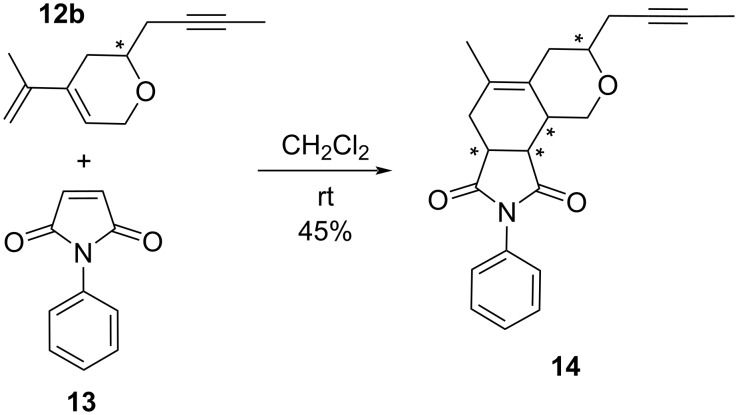
Diels–Alder reaction of dihydropyran **12b** with *N*-phenylmaleimide (**13**).

In general, the RCEYM of enynes leads to products containing a conjugated double bond system, which can undergo a Diels–Alder reaction and further can be employed in the synthesis of biologically active compounds, as for example cacospongiolide B [38], norsalvinorin A [39] or salvinorin A [40]. The substituted dihydropyrans **12** obtained by the RCEYM of oxaenediynes **2** contain both a triple bond and a conjugated diene system. We were interested in the possible orthogonal transformation of the products and hence carried out the Diels–Alder reaction of the dihydropyran **12b** with *N*-phenylmaleimide (**13**) as described in [41]. The reaction at rt in dichloromethane gave the target product **14** in a moderate 45% isolated yield as a mixture of two diastereoisomers (Scheme 14). Pure diastereoisomers were obtained by column chromatography in 24% and 17% yield, respectively.

### DFT study of the Diels–Alder reaction of compounds **12b** and **13**

**Figure 4:**
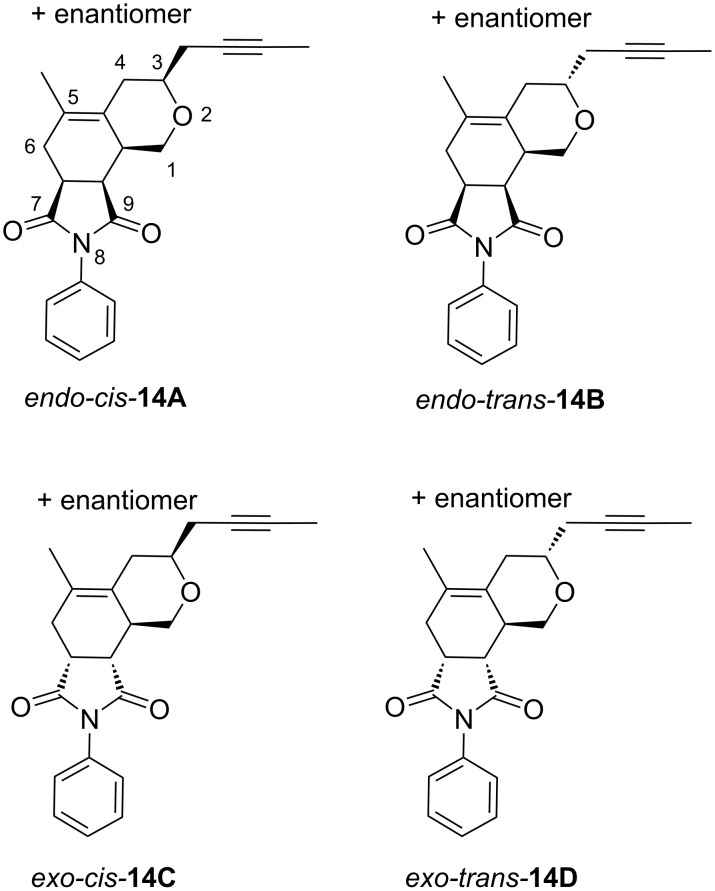
The four possible diastereoisomers of hexahydropyranoisoindole **14**.

Hexahydropyranoisoindole **14** contains four stereogenic centers and hence can form 8 possible diastereoisomers. Due to fixed configuration of maleimide **13**, four possible diastereoisomers come into account (Figure 4).

**Figure 5:**
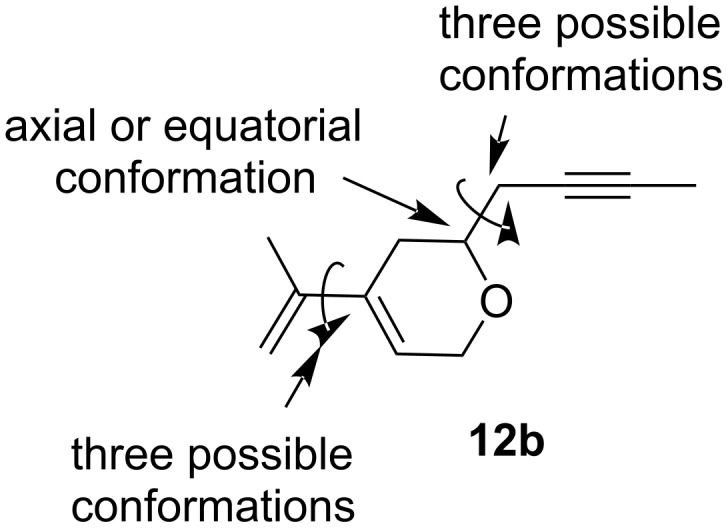
Conformations of dihydropyran **12b**.

With the aim to disclose the most probable candidates for the experimentally obtained two diastereoisomers, we decided to perform a short DFT study of the Diels–Alder reaction between dihydropyran **12b** and maleimide **13**. We started the study with the optimization of the starting compound. While the optimization of the rigid maleimide **13** was straightforward, 18 conformations had to be considered for the dihydropyran **12b**, namely all combinations of equatorial or axial positions of the but-2-ynyl substituent, three staggered conformations of the prop-1-ynyl group with respect to the C–O bond of the ring, and three conformations of the dienyl system (*s*-*trans* and two tilted *s-cis* conformations) (Figure 5).

**Figure 6:**
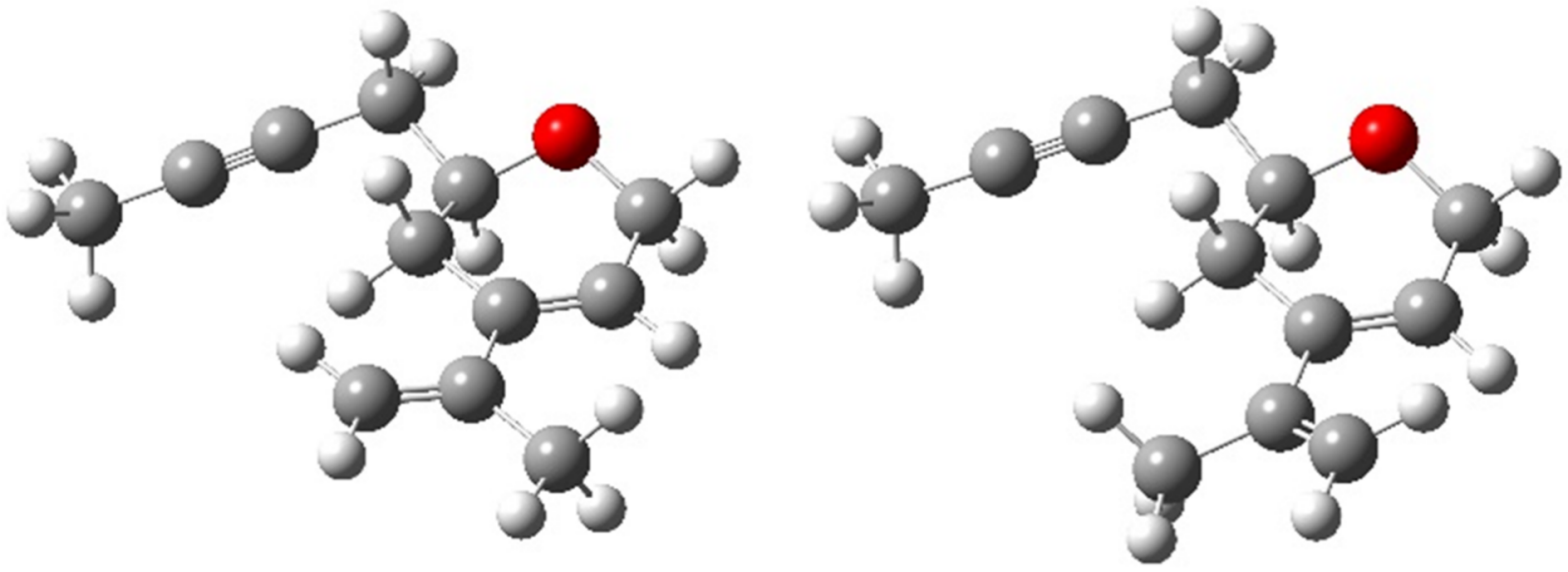
The two most stable *s-trans* (left) and *s-cis* (right) conformations of dihydropyran **12b**.

Among the 18 conformations, the structure with an equatorial conformation of the but-2-ynyl group, an *anti*-arrangement of the O–C bond and the prop-1-ynyl group, and an *s*-*trans* conformation of the diene system proved to be the most stable by more than 7 kJ/mol (Figure 6). Of course, this conformation cannot enter a Diels–Alder reaction and hence the more stable *s*-*cis* conformation with otherwise identical conformational arrangement of the other two parameters, although less stable by about 9 kJ/mol, was considered as the starting geometry for the Diels–Alder reaction (Figure 6).

**Figure 7:**
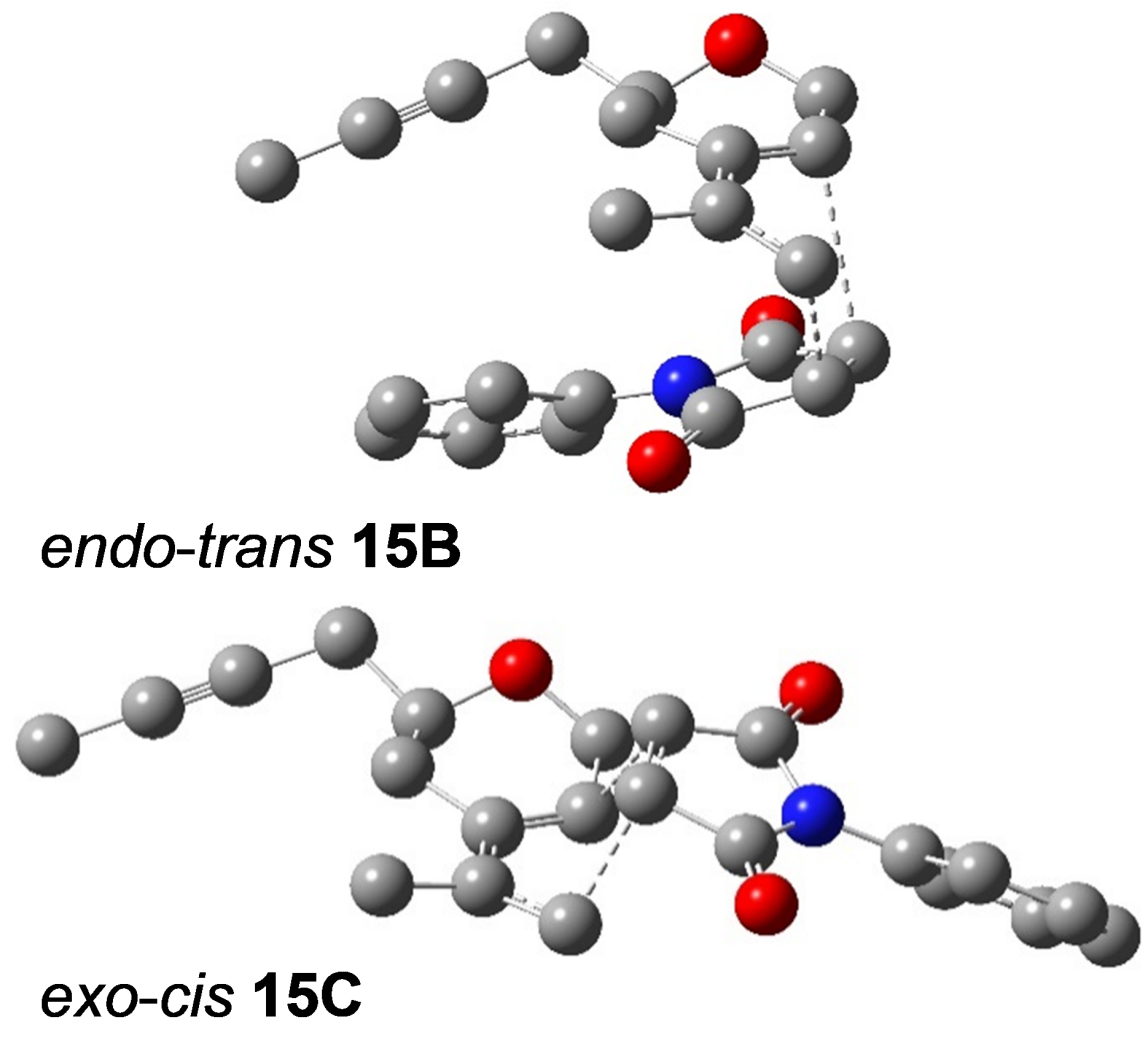
The two most stable transition states *endo-trans* **15B** and *exo*-*cis ***15C** (hydrogens are omitted for clarity).

**Figure 8:**
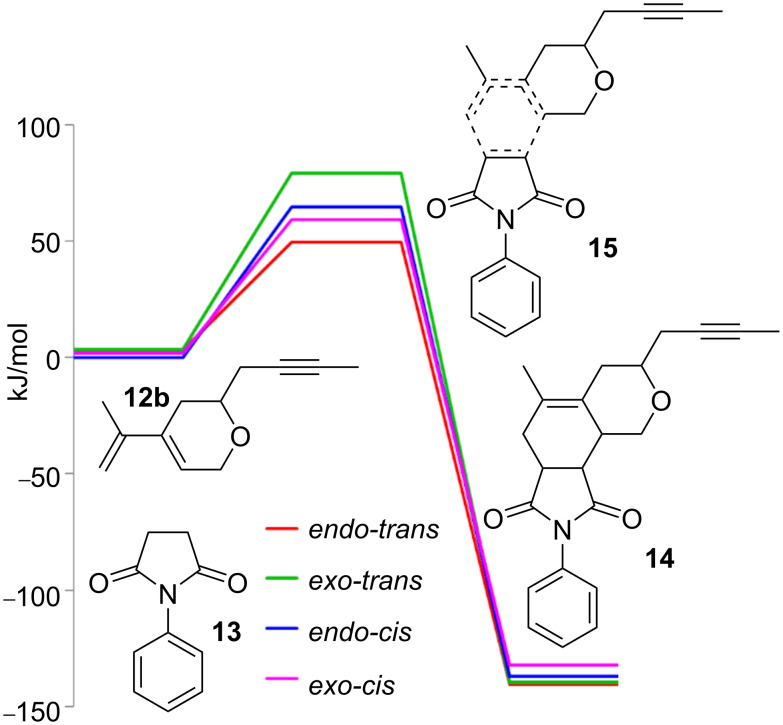
PES of the Diels–Alder reaction of dihydropyran **12b** and maleimide **13**.

The search for the saddle points (starting complexes of both unsaturated compounds **12b** and **13**, transition states **15A**–**D** and the corresponding Diels–Alder products **14A**–**D** (Figure 4)) showed that the reaction is strongly exergonic and irreversible with an activation free Gibbs energy in the range between 46 and 76 kJ/mol. From the activation free Gibbs energy results that two main stereoisomers should be formed, namely the *endo-trans* **14B** (99%) and the *exo*-*cis* **14C** (1%). The difference among the computed (99% and 1%) and experimentally observed values (29% and 16%) can be explained by a participation of other conformations, a lower accuracy of pure functional used, and an incompleteness of the double zeta basis set. The structures of the corresponding transition states **15B**, **15C** are depicted in Figure 7, and the relative free Gibbs energies of all saddle points for all four stereoisomers are shown in Figure 8.

## Conclusion

In the study of the ring-closing enyne metathesis of oxaenediynes, we found that the reaction is highly sensitive to the substitution in the allyloxy chain. While oxaenediynes bearing an unsubstituted allyl chain gave moderate to excellent yields of the target substituted dihydropyrans bearing alkynyl and vinyl groups, a substitution of the allyl group for methallyl, acryloyl or methacryloyl resulted in the complete loss of reactivity or formation of highly complex mixtures. On the other hand, the reaction showed a significantly lower sensitivity to terminal modifications of the triple bonds, tolerating well various alkyl or ester groups. Among the catalysts used, the Grubbs 1st generation catalyst under Mori conditions worked best, while the Grubbs 2nd and Hoveyda–Grubbs 2nd generation precatalysts gave lower yields. For the 2nd generation precatalysts, Mori conditions could not be applied due to multiple side metathesis reactions with ethene. To illustrate possible orthogonal modifications of the products bearing a triple bond and a conjugated diene system, we performed a pilot Diels–Alder reaction with *N*-phenylmaleimide. Two main diastereomers were formed, the structures of which were estimated by DFT computations. The scope and limitations of the oxaenediyne ring-closing enyne metathesis disclosed here gave us essential information for continuing studies on the enantioselective variant of this reaction.

## Experimental

### General comments

Temperature data were uncorrected. NMR spectra were recorded with a Varian MercuryPlus spectrometer (^1^H NMR spectra at 299.97 MHz and ^13^C NMR spectra at 75.77 MHz), or with an Agilent 400-MR DDR2 spectrometer (^1^H NMR spectra at 399.94 MHz and ^13^C NMR spectra at 100.58 MHz) using the residual deuterated solvent signals as the internal standards. Chemical shifts are given in parts per million and coupling constants in hertz. Mass spectra (ESI, APCI) and HRMS spectra were measured with a hybrid LTQ Obitrap XL instrument (Thermo Fisher Scientific). All reactions were performed in a dry inert atmosphere (Ar) in oven-dried flasks. Anhydrous solvents were obtained from a PureSolv MD7 drying line (Innovation Technologies). *N*-Phenylmaleimide (**13**) was prepared according to a literature procedure. [42]. The syntheses of compounds **2**, **4**, and **7**–**11** are given in Supporting Information File 1.

### RCEYM of oxaenediynes

#### General procedure under ethene atmosphere (A)

Anhydrous dichloromethane was degassed and then percolated with ethene for several min. To the oxaenediyne (1 equiv) in CH_2_Cl_2_ was added the Grubbs or Hoveyda–Grubbs catalyst (0.05 equiv) in CH_2_Cl_2_ and the mixture was stirred under an ethene atmosphere at 25 °C for 24 h. Afterwards, the mixture was filtered through a short pad of silica, the silica washed with CH_2_Cl_2_, and the organic solution was carefully evaporated to give the crude products. If necessary, the products were purified by column chromatography.

#### General procedure under argon atmosphere (B)

Anhydrous dichloromethane was degassed before reaction. To the oxaenediyne (1 equiv) in CH_2_Cl_2_ was added the Grubbs or Hoveyda–Grubbs catalyst (0.05 equiv) in CH_2_Cl_2_ and the mixture was stirred under an argon atmosphere at 25 °C for 24 h. Afterwards, the mixture was filtered through a short pad of silica, the silica washed with CH_2_Cl_2_, and the organic solution was carefully evaporated to give the crude products. If necessary, the products were purified by column chromatography.

#### 4-Ethenyl-(2-prop-2-yn-1-yl)-3,6-dihydro-*2H*-pyran (**12a**)

According to general procedure A, from oxaenediyne **2a** (243 mg, 1.64 mmol) in CH_2_Cl_2_ (6 mL) using **G-I** (67.5 mg, 82.0 μmol) in CH_2_Cl_2_ (7 mL) as a catalyst. The crude product was obtained and was further purified by column chromatography (eluent hexane/EtOAc 95:5, *R*_f_ = 0.54) to give dihydropyran **12a** as clear oil (182 mg, 75%). ^1^H NMR (299.97 MHz, CDCl_3_) δ 2.06 (t, *J* = 2.8 Hz, 1H, CH≡C), 2.13–2.23 (m, 1H, CH_2_-C=CH), 2.34 (dt, *J* = 16.7 Hz, *J* = 2.8 Hz, 1H, CH_2_), 2.49 (ddd, *J* = 16.7 Hz, *J* = 6.3 Hz, *J* = 2.8 Hz, 1H, C≡C-CH_2_), 2.56 (ddd, *J* = 16.7 Hz, *J* = 5.6 Hz, *J* = 2.8 Hz, 1H, C≡C-CH_2_), 3.65–3.76 (m, 1H, CH-O), 4.26–4.39 (m, 2H, O-CH_2_), 5.02 (d, *J* = 10.7 Hz, 1H, CH=CH_2_), 5.17 (d, *J* = 17.4 Hz, 1H, CH=CH_2_), 5.73 (m, 1H, C=CH), 6.38 (dd, *J* = 17.4 Hz, *J* = 10.7 Hz, 1H, CH=CH_2_); ^13^C NMR (75.44 MHz, CDCl_3_) δ 25.5 (s, 1C, CH_2_-C≡C), 28.9 (s, 1C, CH_2_-C=CH), 66.0 (s, 1C, O-CH_2_), 70.1 (s, 1C, HC≡C), 71.7 (s, 1C, CH-O), 80.5 (s, 1C, HC≡C), 111.6 (s, 1C, CH=CH_2_), 126.0 (s, 1C, C=CH), 133.2 (s, 1C, C=CH), 137.9 (s, 1C, CH=CH_2_); MS (CI^+^, *m/z*): 149.1 [M + H]^+^ (10), 109.1 [M − C_3_H_3_]^+^ (100), 91.1 [C_7_H_7_]^+^ (45), 81.1 [C_5_H_5_O]^+^ (50); HRMS (CI^+^, *m*/*z*): [M + H]^+^ calcd for C_10_H_13_O, 149.0966; found, 149.0964.

#### 2-(But-2-yn-1-yl)-4-(prop-1-en-2-yl)-3,6-dihydro-2*H*-pyran (**12b**)

According to general procedure A, from oxaenediyne **2b** (130 mg, 0.738 mmol) in CH_2_Cl_2_ (6 mL) using **G-I** (6.1 mg, 7.4 μmol) in CH_2_Cl_2_ (3 mL) as the catalyst. The crude product was obtained and further purified by column chromatography (eluent hexane/EtOAc 95:5, *R*_f_ = 0.41) to give dihydropyran **12b** as clear oil (127 mg, 98%). ^1^H NMR (299.97 MHz, CDCl_3_) δ 1.80 (t, *J* = 2.6 Hz, 3H, C≡C-CH_3_), 1.90 (s, 3H, C-CH_3_), 2.13–2.28 (m, 1H, CH_2_-C=CH), 2.33 (dm, *J* = 16.4 Hz, 1H, CH_2_-C=CH), 2.39–2.54 (m, 2H, CH_2_-C≡C), 3.56–3.66 (m, 1H, CH-O), 4.25–4.43 (m, 2H, O-CH_2_), 4.92 (s, 1H, C=CH_2_), 5.01 (s, 1H, C=CH_2_), 5.77–5.83 (m, 1H, C=CH); ^13^C NMR (75.44 MHz, CDCl_3_) δ 3.6 (s, 1C, C≡C-CH_3_), 20.2 (s, 1C, C-CH_3_), 25.9 (s, 1C, CH_2_-C≡C), 30.4 (s, 1C, CH_2_-C=CH), 66.3 (s, 1C, O-CH_2_), 72.5 (s, 1C, CH-O), 75.1 (s, 1C, C≡C-CH_2_), 77.4 (s, 1C, CH_3_-C≡C), 110.8 (s, 1C, C=CH_2_), 122.0 (s, 1C, C=CH), 134.1 (s, 1C, C=CH), 142.0 (s, 1C, C=CH_2_); MS (EI^+^, *m/z*) (%): 176 [M]^+^ (5), 147 [M − CH_2_=O + H^+^]^+^ (95), 121.1 [M − CH_2_=O − CH≡CH + H]^+^ (100); HRMS (EI^+^, *m*/*z*): [M]^+^ calcd for C_12_H_16_O, 176.1201; found, 176.1208.

#### 4-(But-1-en-2-yl)-2-(pent-2-yn-1-yl)-3,6-dihydro-2*H*-pyran (**12c**)

According to general procedure A, from oxaenediyne **2c** (31 mg, 0.15 mmol) in CH_2_Cl_2_ (1.2 mL) using **G-I** (6.2 mg, 7.6 μmol) in CH_2_Cl_2_ (1.2 mL) as the catalyst. Dihydropyran **12c** was of sufficient purity and obtained as clear oil (27 mg, 87%). ^1^H NMR (299.97 MHz, CDCl_3_) δ 1.09 (t, *J* = 7.3 Hz, 3H, CH_3_-CH_2_-C=CH_2_), 1.13 (t, *J* = 7.5 Hz, 3H, CH_3_-CH_2_-C≡C), 2.19 (qt, *J* = 7.4 Hz, *J* = 2.4 Hz, 2H, CH_3_-CH_2_-C≡C), 2.21–2.39 (m, 4H, CH_2_-C=CH + CH_2_-C=CH_2_), 2.43 (ddt, *J* = 16.6 Hz, *J* = 6.6 Hz, *J* = 2.4 Hz, 1H, C≡C-CH_2_-CH), 2.51 (ddt, *J* = 16.6 Hz, *J* = 5.5 Hz, *J* = 2.4 Hz, 1H, C≡C-CH_2_-CH), 3.59–3.67 (m, 1H, CH-O), 4.27–4.40 (m, 2H, O-CH_2_), 4.92 (s, 1H, C=CH_2_), 5.03 (s, 1H, C=CH_2_), 5.81–5.84 (m, 1H, C=CH); ^13^C NMR (75.44 MHz, CDCl_3_) δ 12.4 (s, 1C, CH_3_-CH_2_-C≡C), 13.2 (s, 1C, CH_3_-CH_2_-C=CH_2_), 14.2 (s, 1C, CH_3_-CH_2_-C≡C), 25.7 (s, 1C, CH_2_-C=CH_2_), 25.9 (s, 1C, C≡C-CH_2_-CH ), 30.9 (s, 1C, CH_2_-C=CH), 66.3 (s, 1C, O-CH_2_), 72.7 (s, 1C, CH-O), 75.3 (s, 1C, CH_3_-CH_2_-C≡C), 83.6 (s, 1C, CH_3_-CH_2_-C≡C), 108.9 (s, 1C, C=CH_2_), 121.3 (s, 1C, C=CH), 133.4 (s, 1C, C=CH), 148.2 (s, 1C, C=CH_2_); MS (CI^+^, *m/z*) (%): 175.1 [M − C_2_H_5_]^+^ (52), 161.1 [M − C_3_H_5_]^+^ (42), 145.1 [M − C_4_H_11_]^+^ (43), 137.1 [M − C_5_H_7_]^+^ (58), 135.1 [M − C_5_H_7_]^+^ (90), 119.1 [M − C_6_H_13_]^+^ (44), 109.1 [C_7_H_9_O]^+^ (100), 107.1 [C_7_H_7_O]^+^ (39), 79.1 [C_7_H_9_]^+^ (70); HRMS (CI^+^, *m*/*z*): [M + H]^+^ calcd for C_14_H_21_O, 205.1592; found, 205.1593.

#### 2-(Hex-2-yn-1-yl)-4-(pent-1-en-2-yl)-3,6-dihydro-2*H*-pyran (**12d**)

According to general procedure B, from oxaenediyne **2d** (25 mg, 0.11 mmol) in CH_2_Cl_2_ (1.5 mL) using **G-II** (4.6 mg, 5.4 μmol) in CH_2_Cl_2_ (1.5 mL) as the catalyst. Dihydropyran **12d** was of sufficient purity and obtained as clear oil (24 mg, 95%). ^1^H NMR (299.97 MHz, CDCl_3_) δ 0.92 (t, *J* = 7.3 Hz, 3H, CH_3_-CH_2_-CH_2_-C=CH_2_), 0.97 (t, *J* = 7.3 Hz, 3H, CH_3_-CH_2_-CH_2_-C≡C), 1.40–1.58 (m, 4H, CH_3_-CH_2_), 2.15 (tt, *J* = 7.0 Hz, *J* = 2.5 Hz, 2H, CH_2_-CH_2_-C≡C), 2.17–2.41 (m, 4H, CH_2_-C=CH + CH_2_-C=CH_2_), 2.43 (ddt, *J* = 16.4 Hz, *J* = 6.8 Hz, *J* = 2.4 Hz, 1H, C≡C-CH_2_-CH), 2.52 (ddt, *J* = 16.4 Hz, *J* = 5.7 Hz, *J* = 2.4 Hz, 1H, C≡C-CH_2_-CH), 3.58–3.68 (m, 1H, CH-O), 4.25–4.40 (m, 2H, O-CH_2_), 4.90 (s, 1H, C=CH_2_), 5.03 (s, 1H, C=CH_2_), 5.79–5.85 (m, 1H, C=CH); ^13^C NMR (75.44 MHz, CDCl_3_) δ 13.5 (s, 1C, CH_3_-CH_2_-CH_2_-C≡C), 14.0 (s, 1C, CH_3_-CH_2_-CH_2_-C=CH_2_), 20.8 (s, 1C, CH_2_-CH_2_-C≡C), 21.9 (s, 1C, CH_2_-CH_2_-C=CH_2_), 22.4 (s, 1C, CH_2_-CH_2_-C≡C), 26.0 (s, 1C, C≡C-CH_2_-CH), 30.8 (s, 1C, CH_2_-C=CH), 35.3 (s, 1C, CH_2_-C=CH_2_), 66.3 (s, 1C, O-CH_2_), 72.7 (s, 1C, CH-O), 76.1 (s, 1C, CH_2_-CH_2_-C≡C), 82.1 (s, 1C, CH_2_-CH_2_-C≡C), 109.9 (s, 1C, C=CH_2_), 121.4 (s, 1C, C=CH), 133.4 (s, 1C, C=CH), 146.6 (s, 1C, C=CH_2_); MS (CI^+^, *m/z*) (%): 189.2 [M − C_3_H_7_]^+^ (41), 151.1 [M − C_6_H_9_]^+^ (51), 149.1 [M − C_6_H_11_]^+^ (100), 133.1 [M − C_7_H_13_]^+^ (47), 123.1 [M − C_8_H_13_]^+^ (48), 107.1 [C_7_H_7_O]^+^ (60), 81.1 [C_5_H_5_O]^+^ (72); HRMS (CI^+^, *m*/*z*): [M + H]^+^ calcd for C_16_H_25_O, 233.1905; found, 233.1902.

#### Methyl 4-[4-(3-methoxy-3-oxoprop-1-en-2-yl)-3,6-dihydro-2*H*-pyran-2-yl]but-2-ynoate (**12e**)

According to general procedure A, from oxaenediyne **2e** (32 mg, 0.12 mmol) in CH_2_Cl_2_ (1.3 mL) using **G-I** (5.0 mg, 6.1 μmol) in CH_2_Cl_2_ (1.3 mL) as the catalyst. Dihydropyran **12e** was of sufficient purity and obtained as clear oil (20 mg, 63%). ^1^H NMR (299.97 MHz, CDCl_3_) δ 2.23–2.34 (m, 2H, CH_2_-C=CH), 2.62 (dd, *J* = 17.2 Hz, *J* = 6.6 Hz, 1H, C≡C-CH_2_), 2.69 (dd, *J* = 17.2 Hz, *J* = 6.0 Hz, 1H, C≡C-CH_2_), 3.77 (s, 3H, CH_3_), 3.78–3.83 (m, 4H, CH + CH_3_), 4.32–4.36 (m, 2H, O-CH_2_), 5.62 (s, 1H, C=CH_2_), 5.97 (s, 1H, C=CH_2_), 6.12–6.15 (m, 1H, C=CH); ^13^C NMR (75.44 MHz, CDCl_3_) δ 25.6 (s, 1C, CH_2_-C≡C), 31.3 (s, 1C, CH_2_-C=CH), 52.0 (s, 1C, CH_3_), 52.6 (s, 1C, CH_3_), 66.0 (s, 1C, O-CH_2_), 71.0 (s, 1C, CH-O), 74.3 (s, 1C, C≡C-CH_2_), 85.4 (s, 1C, CO-C≡C), 122.2 (s, 1C, C=CH_2_), 126.2 (s, 1C, C=CH), 129.9 (s, 1C, CH=C), 140.6 (s, 1C, C=CH_2_), 154.0 (s, 1C, C≡C-C=O), 167.1 (s, 1C, CH_2_=C-C=O); MS (CI^+^, *m/z*) (%): 233.1 [M − CH_3_O]^+^ (100), 167.1 [M − C_5_H_5_O_2_]^+^ (65), 139.1 [M − C_6_H_5_O_3_]^+^ (49); HRMS (CI^+^, *m*/*z*): [M + H]^+^ calcd for C_14_H_17_O_5_, 265.1076; found, 265.1077.

#### 4-[1-(Trimethylsilyl)ethenyl]-2-[3-(trimethylsilyl)prop-2-yn-1-yl]-3,6-dihydro-2*H*-pyran (**12f**)

According to general procedure A, from oxaenediyne **2f** (40 mg, 0.137 mmol) in CH_2_Cl_2_ (1.34 mL) using **G-I** (5.6 mg, 6.9 μmol) in CH_2_Cl_2_ (1.34 mL) as catalyst. A mixture of the target product **12f** and the starting material **11** in a 1:4 ratio was obtained. The product could not be isolated in a pure state and was only identified in ^1^H NMR spectra by comparison of the key signals with analogous compounds. ^1^H NMR (299.97 MHz, CDCl_3_) δ 4.26-4.33 (m, 2H, O-CH_2_), 5.38–5.40 (m, 1H, C=CH_2_), 5.64–5.68 (m, 1H, C=CH_2_), 5.71–5.74 (m, 1H, C=CH). Other ^1^H NMR signals could not be convincingly assigned due to overlapping signals with the starting compound.

### Diels–Alder reaction of dihydropyran **12b** with *N*-phenylmaleimide (**13**)

#### 3-(But-2-yn-1-yl)-5-methyl-8-phenyl-3,4,6,6a,9a,9b-hexahydropyrano[3,4-*e*]isoindole-7,9(1*H*,8*H*)-dione (**14**)

Dihydropyran **12b** (100 mg, 0.567 mmol) and *N*-phenylmaleimide (**13**, 108 mg, 0.624 mmol) were dissolved in CH_2_Cl_2_ (4 mL) and the mixture was stirred for 130 h at rt. After evaporation, the mixture was purified by gradient column chromatography (eluent hexane/EtOAc 2.5:1 → 2.4:1) to give the major diastereomer (probably *endo-trans-***14B**, 50 mg, 25%, white solid, *R*_f_ = 0.17 for hexane/EtOAc 2.5:1) and the minor diastereomer (probably *exo-cis-***14C**, 28 mg, 14%, clear solid, *R*_f_ = 0.24 for hexane/EtOAc 2.5:1). A mixture of the two diastereomers (14 mg, 7%, **14B**/**14C** 2:1) and starting *N*-phenylmaleimide (58 mg, 54%) were also recovered.

Major diastereoisomer (probably *endo-trans-***14B**): ^1^H NMR (299.97 MHz, CDCl_3_) δ 1.76 (t, *J* = 2.6 Hz, 3H, C≡C-CH_3_), 1.77 (s, 3H, C=C-CH_3_), 2.22–2.42 (m, 4H, C≡C-CH_2_ + CH_2_-C=C-CH_3_ + CH_3_-C-CH_2_), 2.58–2.67 (m, 2H, CH_2_-C=C-CH_3_ + O-CH_2_-CH), 2.71 (dd, *J* = 14.5 Hz, *J* = 1.9 Hz, 1H, CH_3_-C-CH_2_), 3.19 (dd, *J* = 8.8 Hz, *J* = 6.9 Hz, 1H, CH-CH-C=O), 3.25–3.30 (m, 1H, CH_2_-CH-C=O), 3.81–3.89 (m, 1H, CH-O), 3.95 (dd, *J* = 11.8 Hz, *J* = 5.7 Hz, 1H, O-CH_2_), 4.46 (dd, *J* = 11.8 Hz, *J* = 8.1 Hz, 1H, O-CH_2_), 7.17–7.20 (m, 2H, C_Ar_-CH_Ar_), 7.33–7.38 (m, 1H, CH_Ar_), 7.40–7.46 (m, 2H, CH_Ar_); ^13^C NMR (75.44 MHz, CDCl_3_) δ 3.6 (s, 1C, C≡C-CH_3_), 19.0 (s, 1C, C=C-CH_3_), 26.6 (s, 1C, C≡C-CH_2_), 28.9 (s, 1C, CH_2_-C=C-CH_3_), 30.6 (s, 1C, CH_3_-C-CH_2_), 37.1 (s, 1C, O-CH_2_-CH), 40.7 (s, 1C, CH_2_-CH-C=O), 42.1 (s, 1C, O-CH_2_-CH-CH), 63.0 (s, 1C, O-CH_2_), 72.9 (s, 1C, CH-O), 75.2 (s, 1C, CH_3_-C≡C), 77.5 (s, 1C, CH_3_-C≡C), 126.3 (s, 2C, C_Ar_-CH_Ar_), 127.4 (s, 1C, CH_3_-C=C), 128.5 (s, 1C, CH_Ar_), 129.0 (s, 1C, CH_3_-C=C), 129.1 (s, 2C, CH_Ar_), 131.9 (s, 1C, C_Ar_), 176.8 (s, 1C, CH-CH-C=O), 178.5 (s, 1C, CH_2_-CH-C=O); MS (ESI^+^, *m/z*) (%): 372.2 [M + Na]^+^ (100), 350.2 [M + H]^+^ (20); HRMS (ESI^+^, *m*/*z*): [M + H]^+^ calcd for C_22_H_24_O_3_N, 350.1751; found, 350.1752; HRMS (ESI^+^, *m*/*z*): [M + Na]^+^ calcd for C_22_H_23_O_3_NNa, 372.1570; found, 372.1571.

Minor diastereoisomer (probably *exo-cis-***14C**): ^1^H NMR (299.97 MHz, CDCl_3_) δ 1.72–1.76 (m, 3H, C=C-CH_3_), 1.78 (t, *J* = 2.6 Hz, 3H, C≡C-CH_3_), 2.16–2.45 (m, 4H, C≡C-CH_2_ + CH_2_-C=C-CH_3_ + CH_3_-C-CH_2_), 2.53–2.74 (m, 3H, CH_2_-C=C-CH_3_ + O-CH_2_-CH + CH_3_-C-CH_2_), 3.15–3.28 (m, 2H, CH-CH-C=O + CH_2_-CH-C=O), 3.60–3.72 (m, 1H, CH-O), 4.16 (dd, *J* = 14.6 Hz, *J* = 11.7 Hz, 1H, O-CH_2_), 4.19 (dd, *J* = 12.8 Hz, *J* = 11.7 Hz, 1H, O-CH_2_), 7.16–7.22 (m, 2H, C_Ar_-CH_Ar_), 7.33–7.39 (m, 1H, CH_Ar_), 7.40–7.48 (m, 2H, CH_Ar_); ^13^C NMR (75.44 MHz, CDCl_3_) δ 3.6 (s, 1C, C≡C-CH_3_), 18.8 (s, 1C, C=C-CH_3_), 26.4 (s, 1C, C≡C-CH_2_), 30.3 (s, 1C, CH_2_-C=C-CH_3_), 30.7 (s, 1C, CH_3_-C-CH_2_), 35.6 (s, 1C, O-CH_2_-CH), 40.1 (s, 1C, CH_2_-CH-C=O), 40.9 (s, 1C, CH-CH-C=O), 66.3 (s, 1C, O-CH_2_), 74.3 (s, 1C, CH-O), 75.0 (s, 1C, CH_3_-C≡C), 77.5 (s, 1C, CH_3_-C≡C), 126.3 (s, 2C, C_Ar_-CH_Ar_), 126.9 (s, 1C, CH_3_-C=C), 127.7 (s, 1C, CH_3_-C=C), 128.5 (s, 1C, CH_Ar_), 129.1 (s, 2C, CH_Ar_), 131.8 (s, 1C, C_Ar_), 177.0 (s, 1C, CH-CH-C=O), 178.5 (s, 1C, CH_2_-CH-C=O); MS (ESI^+^, *m/z*): 372.2 [M + Na]^+^ (100), 350.2 [M + H]^+^ (35); HRMS (ESI^+^, *m*/*z*): [M + H]^+^ calcd for C_22_H_24_O_3_N, 350.1751; found, 350.1752; HRMS (ESI^+^, *m*/*z*): [M + Na]^+^ calcd for C_22_H_23_O_3_NNa, 372.1570; found, 372.1571.

### Computational details

DFT calculations were performed using the Gaussian 16 program suite [43]. For visualizations, the GaussView 6 interface program was used [44]. Due to economy, the pure M06L functional [45], def2-SV(P) basis set [46], and resolution of identity approximation [47] were employed. The solvent (dichloromethane) was simulated using the SMD method [48].

## Supporting Information

File 1 Synthesis of compounds **2**, **4**, and **7**–**11**, copies of ^1^H and ^13^C NMR spectra of compounds **2**, **10**–**12**, and **14**, and XYZ files of all computed structures.
