# Supplementary material for: Ring-closing metathesis of prochiral oxaenediynes to racemic 4-alkenyl-2-alkynyl-3,6-dihydro-2H-pyrans
Source: Beilstein J Org Chem. 2020 Nov 13;16:2757–68. doi: 10.3762/bjoc.16.226 (PMC7670115; doi:10.3762/bjoc.16.226)
Supplement: File 1 — Synthesis of compounds 2, 4, and 7–11, copies of 1H and 13C NMR spectra of compounds 2, 10–12, and 14, and XYZ files of all computed structures. [file Beilstein_J_Org_Chem-16-2757-s001.pdf]

BEILSTEIN JOURNAL OF ORGANIC CHEMISTRY

Supporting Information

for

Ring-closing metathesis of prochiral oxaenediynes to racemic 4-alkenyl-2-alkynyl-3,6-dihydro-2*H*-pyrans

Viola Kolaříková, Markéta Rybáčková, Martin Svoboda and Jaroslav Kvíčala

Beilstein J. Org. Chem. **2020,** *16,* 2757–2768. doi:10.3762/bjoc.16.226

Synthesis of compounds 2, 4, and 7–11, copies of ^{1}H and ^{13}C NMR spectra of compounds 2, 10–12, and 14, and XYZ files of all computed structures

License and Terms: This is a supporting information file under the terms of the Creative Commons Attribution License (https://creativecommons.org/licenses/by/4.0). Please note that the reuse, redistribution and reproduction in particular requires that the authors and source are credited.
The license is subject to the *Beilstein Journal of Organic Chemistry* terms and conditions: (https://www.beilstein-journals.org/bjoc)

Table of contents

1. Experimental details for the synthesis of compounds **2**, **4**, and **7–11** S2

2. Copies of ^{1}H and ^{13}C NMR spectra..S20

3. XYZ files of all computed geometries S39

References..S52

1. Experimental details of synthesis of compounds 2, 4, and 7–11

Synthesis of starting oxaenediynes

Hepta-1,6-diyn-4-ol (4a) [1]

A mixture of Mg (6.69 g, 275 mmol) and $HgCl_2$ (122 mg, 449 mmol) in diethyl ether (130 mL) under an inert atmosphere was cooled to 0 °C and an 80% solution of propargyl bromide in toluene (30 mL, 269 mmol) was added dropwise over 1 h. The mixture was stirred at 0 °C for 1 h, then ethyl formate (7.5 mL, 8.18 g, 92.8 mmol) was added, the mixture was stirred for 2 h at 0 °C, and then quenched with ice water (130 mL) and HCl (1.2 M solution, 260 mL). The organic layer was extracted with Et_2O (3 × 130 mL), the combined organic layers were washed with a saturated $NaHCO_3$ solution (30 mL), and dried over Na_2SO_4. Evaporation gave crude alcohol **4a**, which was distilled at reduced pressure to give the desired hepta-1,6-diyn-4-ol (**4a**) as clear oil (9.02 g, 90%, bp 88-91°C at 3.5 kPa). ^{1}H NMR: δ 2.09 (t, *J* = 2.7 Hz, 2H, C**H**≡C), 2.22 (d, *J* = 5.4 Hz, 1H, O**H**), 2.44-2.63 (m, 4H, C**H**$_2$), 3.97 (m, 1H, C**H**-CH$_2$).

4-(*tert*-Butyldimethylsilyloxy)hepta-1,6-diyne (7a) [1]

tert-Butyldimethylsilyl chloride (2.51 g, 16.7 mmol) and imidazole (1.14 g, 16.7 mmol) were added to a solution of hepta-1,6-diyn-4-ol (**4a**, 1.39 g, 12.8 mmol) in DCM (23 mL) at 0 °C. The reaction mixture was allowed to warm to rt and stirred for 20 h. After quenching with water (23 mL), extraction with DCM (3 × 23 mL), and drying over anhydr. $MgSO_4$, the solvent was evaporated and the crude product was purified by column chromatography (eluent hexane/EtOAc 40:1, Rf = 0.48) to give the desired silyl ether **7a** as clear oil (2.86 g, quant.). ^{1}H NMR [1]: δ 0.12 (s, 6H, Si-C**H**$_3$), 0.91 (s, 9H, C**H**$_3$), 2.00 (t, *J* = 2.6 Hz, 2H, C**H**≡C), 2.44 (ddd, *J* = 16.7 Hz, *J* = 5.9 Hz, *J* = 2.6 Hz, 2H, C**H**$_2$), 2.51 (ddd, *J* = 16.7 Hz, *J* = 5.9 Hz, *J* = 2.6 Hz, 2H, C**H**$_2$), 3.97 (quint, *J* = 5.9 Hz, 1H, C**H**-CH$_2$).

2-(Hepta-1,6-diyn-4-yloxy)tetrahydro-*2H*-pyran (8a)

p-TsOH·H_2O (69 mg, 0.36 mmol) was added to a solution of hepta-1,6-diyn-4-ol (**4a**, 3.89 g, 36.0 mmol) in dichloromethane (57 mL) at 0 °C. Then, 3,4-dihydro-2*H*-pyran (3.33 g, 39.6 mmol) was slowly added, the mixture was stirred for 5 min at 0 °C, and then for further 55 min at rt. The reaction was quenched with satd. $NaHCO_3$ solution (38 mL), the layers were separated, and the aqueous phase was extracted with dichloromethane (3 × 67 mL). The combined organic layers were dried over anhydr. $MgSO_4$, the solvent was carefully evaporated under reduced pressure, and the crude product was purified by column chromatography (eluent hexane/EtOAc 32:1, Rf =0.21) to give the target pyran **8a** as clear oil (6.58 g, 96%). ^{1}H NMR: δ 1.50-1.91 (m, 6H, C**H**$_2$), 2.02 (dt, *J* = 8.8 Hz, *J* = 2.5 Hz, 2H, C≡C**H**), 2.53 (ddd, *J* = 17 Hz, *J* = 5.8 Hz, *J* = 2.7 Hz, 1H, C-C**H**$_2$), 2.57-2.69 (m, 3H, C-C**H**$_2$), 3.46-3.58 (m, 1H, O-C**H**$_2$), 3.90-4.01 (m, 2H, O-C**H**$_2$ + C**H**-CH_2), 4.82 (t, *J* = 3.5 Hz, 1H, O-C**H**-O). ^{13}C NMR: δ 19.2 (s, 1C, **C**H_2-CH_2), 22.9 (s, 1C, **C**H_2-C), 24.5 (s, 1C, **C**H_2-C), 25.3 (s, 1C, **C**H_2-CH_2), 30.5 (s, 1C, **C**H_2-CH_2), 62.3 (s, 1C, O-**C**H_2), 70.1 (s, 1C, **C**≡C), 70.3 (s, 1C, **C**≡CH), 72.9 (s, 1C, **C**H-O), 80.3 (s, 1C, C≡**C**H), 80.5 (s, 1C, C≡**C**H), 97.9 (s, 1C, O-**C**H-O). MS (EI$^+$), *m/z* (%): 153.1 $[M-C_3H_3]^+$ (30), 101.1 $[C_5H_9O_2]^+$ (40), 91.0 $[C_7H_7]^+$ (95), 85.7 $[C_6H_{13}]^+$ (100), 85.0 $[C_5H_9O]$ (95), 67.0 $[C_5H_7]^+$ (88), 65.0 $[C_5H_5]^+$ (95), 56.1 $[C_3H_4O]^+$ (74). HRMS (EI$^+$): calcd. for $C_{12}H_{15}O_2$ ($[M-H]^+$) 191.1072, found 191.1077.

***tert*-Butyldimethyl(nona-2,7-diyn-5-yloxy)silane (7b)**

Butyllithium (6.0 mL, 14.4 mmol, 2.4 M solution in hexanes) was added dropwise at −78 °C to a solution of TBS ether **7a** (800 mg, 3.60 mmol) in dry THF (15 mL). The mixture was stirred at −78 °C for 1 h, then methyl iodide (2.04 g, 14.4 mmol) was

added, the mixture was allowed to warm to rt, and stirred overnight. After quenching with saturated NH_4Cl solution (48 mL), the aqueous layer was extracted with diethyl ether (3 × 40 mL), and dried over anhydr. $MgSO_4$. Evaporation of the solvents and subsequent column chromatography (eluent hexane/EtOAc 60:1, Rf = 0.38) gave TBS ether **7b** as clear oil (850 mg, 94%). ^{1}H NMR: δ 0.11 (s, 6H, Si-C**H**$_3$), 0.91 (s, 9H, C**H**$_3$), 1.78 (t, *J* = 2.4 Hz, 6H, C≡C-C**H**$_3$), 2.31 (dm, *J* = 16.4 Hz, 2H, C**H**$_2$), 2.41 (dm, *J* = 16.4 Hz, 2H, C**H**$_2$), 3.86 (quint, *J* = 6.0 Hz, 1H, C**H**-CH$_2$). ^{13}C NMR: δ -4.7 (s, 2C, Si-**C**H$_3$), 3.5 (s, 2C, C≡C-**C**H$_3$), 18.2 (s, 1C, Si-**C**), 25.8 (s, 3C, Si-C-**C**H$_3$), 27.2 (s, 2C, **C**H$_2$), 71.0 (s, 1C, **C**H), 76.2 (s, 1C, **C**≡C-CH$_3$), 77.3 (s, 1C, C≡**C**-CH$_3$). MS (ESI$^+$), *m/z* (%): 273.1 [M+Na]$^+$ (40). HRMS (ESI): calcd. for $C_{15}H_{27}OSi$ ([M+H]$^+$) 251.1826, found 251.1827. HRMS (ESI): calcd. for $C_{15}H_{26}ONaSi$ ([M+Na]$^+$) 273.1645, found 273.1645.

***tert*-Butyldimethyl(undeca-3,8-diyn-6-yloxy)silane (7c)**

Butyllithium (3.75 mL, 9.00 mmol, 2.4 M solution in hexanes) was added dropwise at −78 °C to a solution of TBS ether **7a** (667 mg, 3.00 mmol) in dry THF (13 mL). The mixture was stirred at −78 °C for 1 h, then ethyl iodide (2.11 g, 13.5 mmol) was added, the mixture was allowed to warm to rt, and then refluxed for 6 h. After quenching with saturated NH_4Cl solution (25 mL), the aqueous layer was extracted with diethyl ether (3 × 25 mL), and dried over anhydr. $MgSO_4$. Evaporation of the solvents and subsequent column chromatography (eluent hexane/CH_2Cl_2 7:1, Rf = 0.23) gave TBS ether **7c** as clear oil (417 mg, 50%). ^{1}H NMR: δ 0.11 (s, 6H, Si-C**H**$_3$), 0.91 (s, 9H, C**H**$_3$), 1.12 (t, *J* = 7.5 Hz, 6H, CH$_2$-C**H**$_3$), 2.16 (qt, *J* = 7.5 Hz, *J* = 2.3 Hz, 4H, C**H**$_2$-CH$_3$), 2.33 (ddt, *J* = 16.3 Hz, *J* = 6.1 Hz, *J* = 2.3 Hz, 2H, CH-C**H**$_2$), 2.43 (ddt, *J* = 16.3 Hz, *J* = 6.1 Hz, *J* = 2.3 Hz, 2H, CH-C**H**$_2$), 3.85 (quint, *J* = 6.1 Hz, 1H,

C**H**-CH_2). ^{13}C NMR: δ -4.6 (s, 2C, Si-**C**H_3), 12.5 (s, 2C, C**H**$_2$-CH_3), 14.2 (s, 2C, CH_2**C**H_3), 18.1 (s, 1C, Si-**C**), 25.8 (s, 3C, Si-C-**C**H_3), 27.3 (s, 2C, CH-**C**H_2), 71.0 (s, 1C, **C**H), 76.4 (s, 1C, **C**≡C-CH_2-CH_3), 83.3 (s, 1C, C≡**C**-CH_2-CH_3). MS (CI^+), *m/z* (%): 263.2 $[M-CH_3]^+$ (51), 221.1 $[M-C_4H_9]^+$ (100), 211.1 $[M-C_5H_7]^+$ (98), 147.1 $[M-C_6H_{15}OSi]^+$ (52), 73.0 $[C_4H_9O]^+$ (47). HRMS (CI^+): calcd. for $C_{17}H_{31}OSi$ ($[M+H]^+$) 279.2144, found 279.2140.

2-(Nona-2,7-diyn-5-yloxy)tetrahydro-2*H*-pyran (8b)

Butyllithium (27 mL, 60 mmol, 2.25 M solution in hexanes) was added dropwise at −78 °C to a solution of THP ether **8a** (2.88 g, 15.0 mmol) in dry THF (60 mL). The mixture was stirred at −78 °C for 1 h, then methyl iodide (8.52 g, 60.0 mmol) was added, the mixture was allowed to warm to rt, and stirred for 20 h. After quenching with saturated NH_4Cl solution (120 mL), the aqueous layer was extracted with diethyl ether (3 × 100 mL) and dried over anhydr. $MgSO_4$. Evaporation of the solvents and subsequent column chromatography (eluent hexane/EtOAc 97:3, Rf = 0.22) gave THP ether **8b** as clear oil (2.88 g, 87%). ^{1}H NMR: δ 1.47-1.90 (m, 12H, 3 × C**H**$_2$ + 2 × C**H**$_3$), 2.35-2.59 (m, 4H, C-C**H**$_2$), 3.46-3.56 (m, 1H, O-C**H**$_2$), 3.80-3.89 (m, 1H, C**H**-CH_2), 3.92-4.02 (m, 1H, O-C**H**$_2$), 4.82 (t, *J* = 3.5 Hz, 1H, O-C**H**-O). ^{13}C NMR: δ 3.49 (s, 1C, **C**H_3), 3.53 (s, 1C, **C**H_3), 19.4 (s, 1C, **C**H_2-CH_2), 23.2 (s, 1C, **C**H_2-C), 25.0 (s, 1C, **C**H_2-C), 25.4 (s, 1C, **C**H_2-CH_2), 30.7 (s, 1C, **C**H_2-CH_2), 62.2 (s, 1C, O-**C**H_2), 73.9 (s, 1C, CH_2-**C**H-O), 75.3 (s, 1C, **C**≡C), 75.5 (s, 1C, **C**≡C), 77.2 (s, 1C, C≡**C**), 77.3 (s, 1C, C≡**C**), 97.7 (s, 1C, O-**C**H-O). MS (CI^+), *m/z* (%): 119.1 $[M-C_5H_9O_2]^+$ (52), 85.1 $[C_5H_9O]^+$ (100), 83.0 $[C_5H_7O]^+$ (44). HRMS (CI^+): calcd. for $C_{14}H_{21}O_2$ ($[M+H]^+$) 221.1542, found 221.1540.

2-(Undeca-3,8-diyn-6-yloxy)tetrahydro-2*H*-pyran (8c)

Butyllithium (6.0 mL, 14.4 mmol, 2.4 M solution in hexanes) was added dropwise at −78 °C to a solution of THP ether **8a** (1.00 g, 5.20 mmol) in dry THF (20 mL). The mixture was stirred at −78 °C for 1 h, then ethyl iodide (3.24 g, 20.8 mmol) was added, the mixture was allowed to warm to rt, and then refluxed for 6 h. After quenching with saturated NH_4Cl solution (48 mL), the aqueous layer was extracted with diethyl ether (3 × 40 mL) and dried over anhydr. $MgSO_4$. Evaporation of the solvents and subsequent column chromatography (eluent hexane/EtOAc 25:1, Rf = 0.27) gave THP ether **8c** as clear oil (660 mg, 51%). ^{1}H NMR: δ 1.12 (t, *J* = 7.5 Hz, 6H, C**H**$_3$), 1.48-1.91 (m, 6H, CH-C**H**$_2$-C**H**$_2$-C**H**$_2$), 2.11-2.21 (m, 4H, C**H**$_2$-CH$_3$), 2.42 (ddt, *J* = 16.6 Hz, *J* = 6.2 Hz, *J* = 2.5 Hz, 1H, C-C**H**$_2$-CH), 2.48-2.62 (m, 3H, C-C**H**$_2$-CH), 3.45-3.55 (m, 1H, O-C**H**$_2$), 3.86 (m, 1H, C**H**-CH$_2$), 3.92-4.05 (m, 1H, O-C**H**$_2$), 4.86 (t, *J* = 3.6 Hz, 1H, O-C**H**-O). ^{13}C NMR: δ 12.4 (s, 1C, **C**H$_2$-CH$_3$), 12.5 (s, 1C, **C**H$_2$-CH$_3$), 14.2 (s, 1C, **C**H$_3$), 14.2 (s, 1C, **C**H$_3$), 19.4 (s, 1 C, **C**H$_2$-CH$_2$), 23.5 (s, 1C, C-**C**H$_2$-CH), 25.2 (s, 1C, C-**C**H$_2$-CH), 25.5 (s, 1C, **C**H$_2$-CH$_2$), 30.7 (s, 1C, **C**H$_2$-CH$_2$), 62.2 (s, 1C, O-**C**H$_2$), 74.1 (s, 1C, C-CH$_2$-**C**H), 75.7 (s, 1C, **C**≡C-CH$_2$-CH$_3$), 75.9 (s, 1C, **C**≡C-CH$_2$-CH$_3$), 83.3 (s, 1C, C≡**C**-CH$_2$-CH$_3$), 83.5 (s, 1C, C≡**C**-CH$_2$-CH$_3$), 97.8 (s, 1C, O-**C**H-O). MS (ESI$^+$), *m/z* (%): 272.2 [M+Na]$^+$ (100). HRMS (ESI$^+$): calcd. for $C_{16}H_{24}O_2Na$ ([M+Na]$^+$) 271.1669, found 271.1669.

2-(Trideca-4,9-diyn-7-yloxy)tetrahydro-2*H*-pyran (8d)

Butyllithium (16.8 mL, 42.0 mmol, 2.5 M solution in hexanes) was added dropwise at −78 °C to a solution of THP ether **8a** (2.31 g, 12.0 mmol) in dry THF (48 mL). The mixture was stirred at −78 °C for 1 h, then propyl iodide (10.2 g, 60.0 mmol) was added, the mixture was allowed to warm to rt, and then refluxed for 24 h. After

quenching with saturated NH_4Cl solution (90 mL), the aqueous layer was extracted with diethyl ether (3 × 75 mL) and dried over anhydr. $MgSO_4$. Filtration, careful evaporation of solvents, and subsequent column chromatography (eluent hexane/EtOAc 30:1, Rf = 0.24) gave THP ether **8d** as clear oil (2.31 g, 70%). ^{1}H NMR: δ 0.97 (t, *J* = 7.4 Hz, 6H, C**H**3), 1.44-1.95 (m, 10H, C**H**2-CH3 + CH-C**H**2-C**H**2-C**H**2), 2.08-2.18 (m, 4H, C**H**2-CH2-CH3), 2.44 (ddt, *J* = 16.7 Hz, *J* = 6.2 Hz, *J* = 2.4 Hz, 1H, C-C**H**2-CH), 2.49-2.62 (m, 3H, C-C**H**2-CH), 3.46-3.56 (m, 1H, O-C**H**2), 3.81-3.92 (m, 1H, C**H**-CH2), 3.93-4.04 (m, 1H, O-C**H**2), 4.86 (t, *J* = 3.5 Hz, 1H, O-C**H**-O). ^{13}C NMR: δ 13.5 (s, 1C, CH2-**C**H3), 13.5 (s, 1C, CH2-**C**H3), 19.4 (s, 1C, **C**H2-CH2), 20.8 (s, 1C, **C**H2-CH2-CH3), 20.8 (s, 1C, **C**H2-CH2-CH3), 22.4 (s, 1C, **C**H2-CH3), 22.4 (s, 1C, **C**H2-CH3), 23.4 (s, 1C, C-**C**H2-CH), 25.2 (s, 1C, C-**C**H2-CH), 25.5 (s, 1C, **C**H2-CH2), 30.7 (s, 1C, **C**H2-CH2), 62.2 (s, 1C, O-**C**H2), 74.2 (s, 1C, C-CH2-**C**H), 76.5 (s, 1C, **C**≡C-CH2-CH2), 77.2 (s, 1C, **C**≡C-CH2-CH2), 81.8 (s, 1C, C≡**C**-CH2-CH2), 81.9 (s, 1C, C≡**C**-CH2-CH2), 97.8 (s, 1C, O-**C**H-O). MS (ESI$^+$), *m/z* (%): 299.3 [M+Na]$^+$ (100). HRMS (ESI$^+$): calcd. for $C_{18}H_{28}O_2Na$ ([M+Na]$^+$) 299.1982, found 299.1982.

Nona-2,7-diyn-5-ol (4b)

From TBS ether **7b**: tetrabutylammonium fluoride (1.18 g, 3.39 mmol, 75% solution in water) in THF (5 mL) was added to TBS ether **7b** (850 mg, 3.39 mmol) in THF (8 mL) and the mixture was stirred for 46 h. Evaporation of the solvents and column chromatography (eluent hexane/EtOAc 20:3, Rf = 0.26) gave alcohol **4b** as clear oil (351 mg, 76%).

From THP ether **8b**: a mixture of THP ether **8b** (2.87 g, 13.0 mmol) and pyridinium *p*-toluenesulfonate (327 mg, 1.30 mmol) in methanol (60 mL) was stirred for 3 days. Evaporation of the solvents and column chromatography (eluent hexane/EtOAc 20:3,

Rf = 0.26) gave alcohol **4b** as clear oil (1.33 g, 75%). ^{1}H NMR: δ 1.81 (t, *J* = 2.4 Hz, 6H, C-C**H**$_3$), 2.35-2.54 (m, 4H, C**H**$_2$), 3.82 (quint, *J* = 5.9 Hz, 1H, C**H**-CH$_2$). ^{13}C NMR: δ 3.6 (s, 2C, **C**H$_3$), 26.4 (s, 2C, **C**H$_2$), 69.0 (s, 1C, **C**H), 74.7 (s, 2C, CH$_2$-**C**≡C), 78.6 (s, 2C, C≡**C**-CH$_3$). MS (ESI$^+$), *m/z* (%): 159.1 $[M+Na]^+$ (27). HRMS (ESI$^+$): calcd. for $C_9H_{12}ONa$ ($[M+Na]^+$) 159.0781, found 159.0782.

Undeca-3,8-diyn-6-ol (4c)

From TBS ether **7c**: tetrabutylammonium fluoride (272 mg, 0.779 mmol, 75% solution in water) in THF (2 mL) was added to TBS ether **7c** (217 mg, 0.779 mmol) in THF (1 mL) and the mixture was stirred for 5 days. Evaporation of the solvents and column chromatography (eluent hexane/EtOAc 5:1, Rf = 0.48) gave alcohol **4c** as clear oil (100 mg, 78%).

From THP ether **8c**: a mixture of THP ether **8c** (639 mg, 2.57 mmol) and pyridinium *p*-toluenesulfonate (65 mg, 0.26 mmol) in methanol (13 mL) was stirred for 4 days. Evaporation of solvents and column chromatography (eluent hexane/EtOAc 5:1, Rf = 0.48) gave alcohol **8c** as clear oil (272 mg, 64%). ^{1}H NMR: δ 1.14 (t, *J* = 7.5 Hz, 6H, CH$_2$-C**H**$_3$), 2.19 (qt, *J* = 7.5 Hz, *J* = 2.4 Hz, 4H, C**H**$_2$-CH$_3$), 2.22-2.26 (bs, 1H, OH), 2.37-2.55 (m, 4H, CH-C**H**$_2$), 3.77-3.88 (m, 1H, C**H**-CH$_2$). ^{13}C NMR: δ 12.4 (s, 2C, **C**H$_2$-CH$_3$), 14.2 (s, 2C, **C**H$_3$), 26.4 (s, 2C, CH-**C**H$_2$), 69.0 (s, 1C, **C**H), 74.9 (s, 1C, **C**≡C-CH$_2$-CH$_3$), 84.7 (s, 1C, C≡**C**-CH$_2$-CH$_3$). MS (EI$^+$), *m/z* (%): 135.1 $[M-C_2H_5]^+$ (35), 97.1 $[M-C_5H_7]^+$ (37), 79.1 $[M-C_5H_9O]^+$ (37), 67.0 $[M-C_6H_9O]^+$(100), 53.0 $[C_4H_5]^+$(53). HRMS (EI$^+$): calcd. for $C_{11}H_{15}O$ ($[M-H]^+$) 163.1123, found 163.1125.

Trideca-4,9-diyn-7-ol (4d)

A mixture of THP ether **8d** (2.31 g, 8.35 mmol) and pyridinium *p*-toluenesulfonate (210 mg, 0.835 mmol) in methanol (41 mL) was stirred for 3 days. Evaporation of the solvents and column chromatography (eluent hexane/EtOAc 7:1, Rf = 0.46) gave alcohol **4d** as clear oil (851 mg, 53%). ^{1}H NMR: δ 0.98 (t, *J* = 7.4 Hz, 6H, CH_2-C**H**$_3$), 1.47-1.57 (m, 4H, C**H**$_2$-CH_3), 2.15 (tt, *J* = 7.0 Hz, *J* = 2.4 Hz, 4H, C**H**$_2$-CH_2-CH_3), 2.23 (d, *J* = 5.3 Hz, 1H, OH), 2.40-2.53 (m, 4H, CH-C**H**$_2$), 3.78-3.87 (m, 1H, C**H**-CH_2). ^{13}C NMR: δ 13.5 (s, 2C, CH_2-**C**H_3), 20.8 (s, 2C, **C**H_2-CH_2-CH_3), 22.4 (s, 2C, CH_2-**C**H_2-CH_3), 26.4 (s, 2C, CH-**C**H_2), 69.1 (s, 1C, **C**H), 75.7 (s, 1C, **C**≡C-CH_2-CH_2), 83.2(s, 1C, C≡**C**-CH_2-CH_2). MS (EI$^+$), *m/z* (%): 163.1 $[M-C_2H_5]^+$ (45), 145.1 $[M-C_2H_7O]^+$ (55), 93.1 $[C_7H_9]^+$ (57), 91.0 $[C_7H_7]^+$ (52), 79.1 $[C_6H_7]^+$ (51), 77.0 $[C_6H_5]^+$ (48), 67.0 $[C_5H_7]^+$(100), 53.0 $[C_4H_5]^+$(63). HRMS (EI$^+$): calcd. for $C_{13}H_{19}O$ ($[M-H]^+$) 191.1436, found 191.1432.

4-(Allyloxy)hepta-1,6-diyne (2a)

Sodium hydride (1.12 g, 60% in oil, 28.0 mmol) was added to a mixture of hepta-1,6-diyn-4-ol (**4a**, 1.84 g, 16.0 mmol), allyl bromide (3.39 g, 28.0 mmol) and tetraethylammonium iodide (477 mg, 1.86 mmol) in dry THF (40 mL) at 0 °C under an argon atmosphere. The mixture was stirred at 0 °C for 10 min, then allowed to warm to rt, and stirred for 2 days. After the addition of satd. NH_4Cl solution (20 mL), the mixture was extracted with Et_2O (3 × 30 mL) and dried over anhydr. $MgSO_4$. Filtration and evaporation gave the crude product, which was purified by column chromatography (hexane/EtOAc 25:1, Rf = 0.5) to give 4-(allyloxy)hepta-1,6-diyne (**2a**) as pale yellow oil (2.22 g, 94%). ^{1}H NMR: δ 2.00 (t, *J* = 2.7 Hz, 2H, C≡C**H**), 2.44-2.57 (m, 4H, CH-C**H**$_2$), 3.62 (quint, *J* = 5.7 Hz, 1H, C**H**), 4.07 (dm, *J* = 6.0 Hz, 2H, O-

C**H**$_2$), 5.15 (d, *J* = 10.3 Hz, 1H, CH=C**H**$_2$), 5.27 (dm, *J* = 17.2 Hz, 1H, CH=C**H**$_2$), 5.83-5.94 (m, 1H, C**H**=CH$_2$). ^{13}C NMR: δ 23.2 (s, 2C, C-**C**H$_2$), 70.3 (s, 1C, O-**C**H$_2$), 70.5 (s, 2C, **C**≡C), 75.0 (s, 1C, **C**H-CH$_2$), 80.2 (s, 2C, C≡**C**H), 117.2 (s, 1C, CH=**C**H$_2$), 134.4 (s, 1C, **C**H=CH$_2$). MS (ESI$^-$), *m/z* (%): 147 [M-H]$^-$ (20), 127 [M-H$_2$-H$_2$O]$^-$ (26), 109 [M-C$_3$H$_3$]$^-$ (34), 89 [M-C$_3$H$_7$O]$^-$ (36), 87 [M-C$_3$H$_9$O]$^-$ (59), 75 [M-C$_4$H$_{10}$O]$^-$ (100), 67 [M-C$_5$H$_5$O]$^-$ (96). HRMS (ESI$^-$): calcd. for C$_{10}$H$_{11}$O ([M-H]$^-$) 147.0815, found 147.0812.

5-(Allyloxy)nona-2,7-diyne (2b)

Sodium hydride (131 mg, 60% in oil, 3.27 mmol) was added to a mixture of nona-2,7-diyn-5-ol (**4b**, 254 mg, 1.87 mmol), allyl bromide (396 g, 3.27 mmol), and tetraethyl-ammonium iodide (60 mg, 0.22 mmol) in dry Et$_2$O (20 mL) at 0 °C. The mixture was stirred at 0 °C for 10 min, then allowed to warm to rt and stirred for 3 days. After the addition of saturated NH$_4$Cl solution (7 mL), the mixture was extracted with Et$_2$O (2 × 17 mL), washed with brine (17 mL), and dried over anhydr. MgSO$_4$. Filtration and evaporation gave the crude product, which was purified by column chromatography (eluent hexane/EtOAc 50:1, Rf = 0.23) to give 5-(allyloxy)nona-2,7-diyne (**2b**) as clear oil (314 mg, 95%). ^{1}H NMR: δ 1.78 (t, *J* = 2.5 Hz, 6H, C**H**$_3$), 2.37-2.56 (m, 4H, C-C**H**$_2$), 3.54 (quint, *J* = 5.7 Hz, 1H, C**H**), 4.09 (dt, *J* = 5.7 Hz, *J* = 1.4 Hz, 2H, C**H**$_2$), 5.17 (ddm, *J* = 10.4 Hz, *J* = 1.4 Hz, 1H, CH=C**H**$_2$), 5.29 (dm, *J* = 17.2 Hz, 1H, CH=C**H**$_2$), 5.85-6.00 (m, 1H, C**H**=CH$_2$). ^{13}C NMR: δ 3.6 (s, 2C, **C**H$_3$), 23.7 (s, 2C, C-**C**H$_2$), 70.6 (s, 1C, O-**C**H$_2$), 75.2 (s, 1C, **C**H), 76.2 (s, 1C, CH$_2$-**C**≡C), 77.5 (s, 1C, C≡**C**-CH$_3$), 117.1 (s, 1C, CH=**C**H$_2$), 134.9 (s, 1C, **C**H=CH$_2$). MS (ESI$^+$), *m/z* (%): 199.1 [M+Na]$^+$ (100). HRMS (ESI$^+$): calcd. for C$_{12}$H$_{17}$O ([M+H]$^+$) 177.1274, found 177.1275. HRMS (ESI$^+$): calcd. for C$_{12}$H$_{16}$ONa ([M+Na]$^+$) 199.1093, found 199.1094.

6-(Allyloxy)undeca-3,8-diyne (2c)

Sodium hydride (116 mg, 60% in oil, 2.90 mmol) was added to a mixture of undeca-3,8-diyn-6-ol (**4c**, 272 mg, 1.66 mmol), allyl bromide (351 mg, 2.90 mmol), and tetraethylammonium iodide (53 mg, 0.19 mmol) in dry THF (7 mL) at 0 °C. The mixture was stirred at 0 °C for 10 min, then allowed to warm to rt and stirred for 4 days. After the addition of saturated NH_4Cl solution (7 mL), the mixture was extracted with Et_2O (2 × 16 mL) and dried over anhydr. $MgSO_4$. Filtration and careful evaporation gave the crude product, which was purified by column chromatography (eluent hexane/EtOAc 50:1, Rf = 0.25) to give 6-(allyloxy)undeca-3,8-diyne (**2c**) as clear oil (303 mg, 89%). ^{1}H NMR: δ 1.13 (t, *J* = 7.5 Hz, 6H, C**H**$_3$), 2.18 (qt, *J* = 7.5 Hz, *J* = 2.4 Hz, 4H, C**H**$_2$-CH$_3$), 2.38-2.57 (m, 4H, CH-C**H**$_2$), 3.56 (quint, *J* = 5.8 Hz, 1H, C**H**), 4.12 (dm, *J* = 5.6 Hz, 2H, O-C**H**$_2$), 5.18 (dm, *J* = 10.4 Hz,1H, CH=C**H**$_2$), 5.31 (dm, *J* = 17.2 Hz, 1H, CH=C**H**$_2$), 5.86-6.02 (m, 1H, C**H**=CH$_2$). ^{13}C NMR: δ 12.5 (s, 2C, **C**H$_2$-CH$_3$), 14.2 (s, 2C, **C**H$_3$), 23.9 (s, 2C, CH-**C**H$_2$), 70.7 (s, 1C, O-**C**H$_2$), 75.6 (s, 1C, **C**≡C-CH$_2$-CH$_3$), 76.4 (s, 1C, **C**H), 83.5 (s, 1C, C≡**C**-CH$_2$-CH$_3$), 117.0 (s, 1C, CH=**C**H$_2$), 135.0 (s, 1C, **C**H=CH$_2$). MS (EI$^+$), *m/z* (%): 137.1 $[M-C_5H_7]^+$ (97), 95.0 $[C_7H_{11}]^+$ (72), 91.0 $[C_7H_7]^+$ (84), 79.0 $[C_6H_7]^+$ (85), 67.0 $[C_5H_7]^+$ (100), 57.0 $[C_3H_5O]^+$ (95). HRMS (EI$^+$): calcd. for $C_{14}H_{19}O$ ($[M-H]^+$) 203.1436, found 203.1432.

7-(Allyloxy)trideca-4,9-diyne (2d)

Sodium hydride (313 mg, 60% in oil, 7.83 mmol) was added to a mixture of trideca-4,9-diyn-7-ol (**4d**, 851 mg, 4.47 mmol), allyl bromide (947 mg, 7.83 mmol), and tetraethylammonium iodide (144 mg, 0.519 mmol) in dry Et_2O (19 mL) at 0 °C. The mixture was stirred at 0 °C for 10 min, then allowed to warm to rt and stirred for 2 days. After the addition of saturated NH_4Cl solution (19 mL), the mixture was

extracted with Et_2O (3 × 29 mL) and dried over anhydr. $MgSO_4$. Filtration and evaporation gave the crude product, which was purified by column chromatography (eluent hexane/EtOAc 50:1, Rf = 0.35) to give 7-(allyloxy)trideca-4,9-diyne (**2d**) as clear oil (927 mg, 89%). ^{1}H NMR: δ 0.98 (t, *J* = 7.5 Hz, 6H, C**H**$_3$), 1.52 (m, 4H, C**H**$_2$-CH$_3$), 2.14 (tt, *J* = 7.0 Hz, *J* = 2.4 Hz, 4H, C**H**$_2$-CH$_2$-CH$_3$), 2.43-2.56 (m, 4H, CH-C**H**$_2$), 3.57 (quint, *J* = 5.8 Hz, 1H, C**H**), 4.12 (dt, *J* = 5.6 Hz, *J* =1.4 Hz, 2H, O-C**H**$_2$), 5.18 (dm, *J* = 10.4 Hz,1H, CH=C**H**$_2$), 5.31 (dm, *J* = 17.3 Hz, 1H, CH=C**H**$_2$), 5.88-5.99 (m, 1H, C**H**=CH$_2$). ^{13}C NMR: δ 13.5 (s, 2C, **C**H$_3$), 20.8 (s, 2C, **C**H$_2$-CH$_2$-CH$_3$), 22.4 (s, 2C, CH$_2$-**C**H$_2$-CH$_3$), 23.9 (s, 2C, CH-**C**H$_2$), 70.7 (s, 1C, O-**C**H$_2$), 76.4 (s, 1C, **C**≡C-CH$_2$-CH$_2$), 76.5 (s, 1C, **C**H), 82.0 (s, 1C, C≡**C**-CH$_2$-CH$_2$), 117.0 (s, 1C, CH=**C**H$_2$), 135.0 (s, 1C, **C**H=CH$_2$). MS (CI$^+$), *m/z* (%): 175.1 $[M-C_3H_5O]^+$ (23), 151.1 $[M-C_6H_9]^+$ (100), 133.1 $[M-C_6H_{11}O]^+$ (30), 81.1 $[C_6H_9]^+$ (31). HRMS (CI$^+$): calcd. for $C_{16}H_{25}O$ ($[M+H]^+$) 233.1905, found 233.1906.

5-[(2-Methylallyl)oxy]nona-2,7-diyne (9b)

Sodium hydride (105 mg, 60% in oil, 2.62 mmol) was added to a mixture of nona-2,7-diyn-5-ol (**4b**, 204 mg, 1.50 mmol), 2-methylprop-2-enyl chloride (407 mg, 4.49 mmol), and tetraethylammonium iodide (125 mg, 0.449 mmol) in dry Et_2O (6 mL) at 0 °C. The mixture was stirred at 0 °C for 10 min, then allowed to warm to rt and stirred for 5 days. After the addition of saturated NH_4Cl solution (6 mL), the mixture was extracted with Et_2O (2 × 10 mL) and dried over anhydr. $MgSO_4$. Filtration, evaporation, and separation by column chromatography (eluent hexane/EtOAc 50:1, Rf = 0.30) gave ether **9b** as clear oil (217 mg, 76%). ^{1}H NMR: δ 1.77-1.81 (m, 9H, C**H**$_3$), 2.39-2.57 (m, 4H, CH-C**H**$_2$), 3.53 (quint, *J* = 5.7 Hz, 1H, C**H**), 4.00 (s, 2H, O-C**H**$_2$), 4.91 (s, 1H, CH=C**H**$_2$), 5.00 (s, 1H, CH=C**H**$_2$). ^{13}C NMR: δ 3.6 (s, 2C, C≡C-

CH3), 19.5 (s, 1C, CH2=C-**C**H3), 23.6 (s, 2C, CH-**C**H2), 73.3 (s, 1C, O-**C**H2), 75.3 (s, 1C, **C**≡C-CH3), 75.9 (s, 1C, **C**H), 77.4 (s, 1C, C≡**C**-CH3), 112.7 (s, 1C, C=**C**H2), 142.3 (s, 1C, **C**=CH2). MS (ESI$^+$), *m/z* (%): 213.1 $[M+Na]^+$ (100). HRMS (ESI$^+$): calcd. for $C_{13}H_{19}O$ ($[M+H]^+$) 191.1430, found 191.1429. HRMS (ESI$^+$): calcd. for $C_{13}H_{18}ONa$ ($[M+Na]^+$) 213.1250, found 213.1248.

7-[(2-Methylallyl)oxy]trideca-4,9-diyne (9d)

Sodium hydride (70 mg, 60% in oil, 1.8 mmol) was added to a mixture of trideca-4,9-diyn-7-ol (**4d**, 192 mg, 1.00 mmol), 2-methylprop-2-enyl chloride (272 mg, 3.00 mmol), and tetraethylammonium iodide (83 mg, 0.30 mmol) in dry Et_2O (4 mL) at 0 °C. The mixture was stirred at 0 °C for 10 min, then allowed to warm to rt and stirred for 7 days. After the addition of saturated NH_4Cl solution (4 mL), the mixture was extracted with Et_2O (2 × 7 mL) and dried over anhydr. $MgSO_4$. Filtration, evaporation, and separation by gradient column chromatography (eluent hexane/EtOAc 65:1→20:1, Rf = 0.32 for hexane/EtOAc 55:1) gave ether **9d** as clear oil (131 mg, 53%). Starting trideca-4,9-diyn-7-ol (73 mg, 38%) was partially recovered. ^{1}H NMR: δ 0.97 (t, *J* = 7.5 Hz, 6H, CH_2-C**H**3), 1.44-1.57 (m, 4H, C**H**2-CH_3), 2.14 (tt, *J* = 7.0 Hz, *J* = 2.3 Hz, 4H, C**H**2-CH_2-CH_3), 2.41-2.58 (m, 4H, CH-C**H**2), 3.55 (quint, *J* = 5.7 Hz, 1H, C**H**), 4.01 (s, 2H, O-C**H**2), 4.88-4.91 (s, 1H, C=C**H**2), 4.99-5.02 (s, 1H, C=C**H**2). ^{13}C NMR: δ 13.5 (s, 2C, CH_2-**C**H3), 19.5 (s, 1C, CH_2=C-**C**H3), 20.8 (s, 2C, **C**H2-CH_2-CH_3), 22.4 (s, 2C, CH_2-**C**H2-CH_3), 23.8 (s, 2C, CH-**C**H2), 73.4 (s, 1C, O-**C**H2), 76.3 (s, 1C, **C**H), 76.4 (**C**≡C-CH_2-CH_2), 81.9 (s, 1C, **C**≡C-CH_2-CH_2), 112.5 (s, 1C, C=**C**H2), 142.4 (s, 1C, **C**=CH_2). MS (EI$^+$), *m/z* (%): 165.1 $[M-C_6H_9]^+$ (100), 123.1 $[C_8H_{11}O]^+$ (57), 121.1 $[C_8H_9O]^+$ (90), 109.1 $[C_7H_9O]^+$ (68), 105.1 $[C_7H_5O]^+$ (74), 95.1 $[C_6H_7O]^+$ (75), 93.1 $[C_7H_9]^+$ (98), 91.0 $[C_6H_3O]^+$ (85), 81.0 $[C_6H_9]^+$ (65), 79.0

$[C_6H_7]^+$ (93), 77.0 $[C_6H_5]^+$ (67), 71.0 $[C_4H_7O]^+$ (85), 55.0 $[C_3H_3O]^+$ (95). HRMS (EI^+): calcd. for $C_{17}H_{26}O$ ($[M]^+$) 246.1984, found 246.1985.

Dimethyl 5-(allyloxy)nona-2,7-diynedioate (2e)

Butyllithium (3.4 mL, 7.7 mmol, 2.3 M solution in hexanes) was added dropwise at −78 °C to a solution of oxaenediyne **2a** (520 mg, 3.51 mmol) in dry THF (13 mL). The mixture was stirred at −78 °C for 1 h, then methyl chloroformate (1.53 g, 16.1 mmol) was added, and the mixture was allowed to warm to rt over 4 h. After quenching with saturated NH_4Cl solution (13 mL), the aqueous layer was extracted with diethyl ether (3 × 13 mL) and dried over anhydr. $MgSO_4$. Filtration and evaporation of the solvents and subsequent column chromatography (eluent hexane/Et_2O 3:1, Rf=0.26) gave diester **2e** as clear oil (751 mg, 81%). ^{1}H NMR: δ 2.70 (d, J = 5.6 Hz, 4H, CH-C**H**$_2$), 3.73-3.83 (m, 7H, C**H**-O + C**H**$_3$), 4.11 (dm, J = 5.7 Hz, 2H, O-C**H**$_2$), 5.23 (dm, J = 10.2 Hz, 1H, CH=C**H**$_2$), 5.32 (dm, J = 17.4 Hz, 1H, CH=C**H**$_2$), 5.84-5.98 (m, 1H, C**H**=CH$_2$). ^{13}C NMR: δ 24.1 (s, 2C, CH-**C**H$_2$), 52.7 (s, 2C, **C**H$_3$), 71.1 (s, 1C, O-**C**H$_2$), 73.9 (s, 1C, **C**H-O), 74.9 (s, 2C, **C**≡C-CO), 84.7 (s, 2C, C≡**C**-CO), 118.0 (s, 1C, CH=**C**H$_2$), 134.0 (s, 1C, **C**H=CH$_2$), 153.8 (s, 2C, **C**=O). MS (CI^+), *m/z* (%): 265.1 $[M+H]^+$ (38), 201.1 $[M-C_2H_7O_2]^+$ (47), 191.0 $[M-C_3H_5O_2]^+$ (45), 173.1 $[M-C_3H_7O_3]^+$ (85), 167.1 $[M-C_5H_5O_2]^+$ (100), 145.1 $[M-C_4H_7O_4]^+$ (65), 117.1 $[M-C_5H_7O_5]^+$ (46), 107.1 $[C_8H_{11}]^+$ (61), 79.1 $[C_6H_7]^+$ (64). HRMS (CI^+): calcd. for $C_{14}H_7O_5$ ($[M+H]^+$) 265.1076, found 265.1078.

[4-(Allyloxy)hepta-1,6-diyne-1,7-diyl]bis(trimethylsilane) (2f)

Butyllithium (2.7 mL, 6.1 mmol, 2.25 M solution in hexanes) was added dropwise at −78 °C to a solution of oxaenediyne **2a** (222 mg, 1.50 mmol) in dry THF (7 mL). The

mixture was stirred at −78 °C for 1 h, then trimethylsilyl chloride (660 mg, 6.08 mmol) was added, the mixture was allowed to warm to rt, and then stirred for 26 h. After quenching with saturated NH_4Cl solution (8 mL), the aqueous layer was extracted with diethyl ether (3 × 5 mL) and dried over anhydr. Na_2SO_4. Evaporation of the solvents and subsequent column chromatography (eluent hexane/EtOAc 98:2, Rf = 0.34) gave silylated oxaenediyne **2f** as clear oil (140 mg, 32%). ^{1}H NMR (299.97 MHz, $CDCl_3$): δ 0.16 (s, 18H, Si-C**H**$_3$), 2.46-2.61 (m, 4H, C**H**$_2$), 3.66 (quint, *J* = 6.0 Hz, 1H, C**H**), 4.16 (dm, *J* = 5.3 Hz, 2H, O-C**H**$_2$), 5.19 (d, *J* = 10.3 Hz, 1H, CH=C**H**$_2$), 5.32 (dm, *J* = 17.1 Hz, 1H, CH=C**H**$_2$), 5.87-6.01 (m, 1H, C**H**=CH$_2$). ^{13}C NMR (75.44 MHz, $CDCl_3$): δ 0.0 (s, 6C, Si-**C**H$_3$), 25.5 (s, 2C, **C**H$_2$-C≡C), 71.1 (s, 1C, O-**C**H$_2$), 75.9 (s, 1C, **C**H), 86.6 (s, 2C, C≡**C**-Si), 103.3 (s, 2C, **C**≡C-Si), 117.1 (s, 1C, CH=**C**H$_2$), 134.8 (s, 1C, **C**H=CH$_2$). MS (ESI$^+$), *m/z* (%): 315.2 [M+Na]$^+$ (21), 156.1 [$C_8H_{16}OSi$]$^+$ (81), 116.1 [$C_6H_{16}Si$]$^+$ (78), 105.1 [C_7H_5O]$^+$ (100). HRMS (ESI$^+$): calcd. for $C_{16}H_{28}ONaSi_2$ ([M+Na]$^+$) 315.1571, found 315.1567.

Hepta-1,6-diyn-4-yl acrylate (10a)

Freshly distilled acryloyl chloride (462 mg, 5.10 mmol) in CH_2Cl_2 was added dropwise to a solution of diynol **4a** (368 mg, 3.40 mmol) and triethylamine (688 mg, 6.80 mmol) in CH_2Cl_2 (10 mL). After stirring for 10 min at 0 °C, the mixture was allowed to warm to rt and stirred for 6 h. The reaction was quenched with water (20 mL), extracted with CH_2Cl_2 (3 × 10 mL), and dried over anhydr. Na_2SO_4. Careful evaporation gave the crude product, which was purified by column chromatography (eluent hexane/EtOAc 95:5, Rf = 0.42) to give acrylate **10a** as clear oil (329 mg, 60%). ^{1}H NMR: δ 2.04 (t, *J* = 2.7 Hz, 2H, C≡C**H**), 2.61-2.78 (m, 4H, C-C**H**$_2$), 5.12 (quint, *J* = 5.8 Hz, 1H, CH$_2$-C**H**), 5.89 (dd, *J* = 10.4 Hz, *J* = 1.4 Hz, 1H, CH=C**H**$_2$), 6.15 (dd, *J* =

17.3 Hz, *J* = 10.4 Hz, 1H, CH=C**H**$_2$), 6.47 (dd, *J* = 17.3 Hz, *J* = 1.4 Hz, 1H, **C**H=CH$_2$). ^{13}C NMR: δ 22.8 (s, 2C, CH-**C**H$_2$), 69.7 (s, 1C, **C**H-CH$_2$), 71.0 (s, 1C, **C**≡CH), 78.8 (s, 1C, C≡**C**H), 128.1 (s, 1C, **C**H=CH$_2$), 131.6 (s, 1C, CH=**C**H$_2$), 165.3 (s, 1C, **C**=O). MS (CI$^+$), *m/z* (%): 163.1 [M+H]$^+$ (50), 123.0 [M-C_3H_3]$^+$ (100), 91.1 [M-$C_3H_3O_2$]$^+$ (80). HRMS (CI$^+$): calcd. for $C_{10}H_{11}O_2$ ([M+H]$^+$) 163.0759, found 163.0757.

Nona-2,7-diyn-5-yl acrylate (10b)

Nona-2,7-diyn-5-ol (**4b**, 250 mg, 1.84 mmol) and triethylamine (372 mg, 3.68 mmol) were dissolved in dry CH_2Cl_2 (6 mL) and the mixture was cooled to 0 °C. Freshly distilled acryloyl chloride (250 mg, 2.76 mmol) was added dropwise and the mixture was stirred for 1 h at 0 °C. After quenching with brine (4 mL), the layers were separated, the aqueous phase extracted with Et_2O (2 × 6 mL), and the combined organic layers were dried over anhydr. Na_2SO_4. Careful evaporation gave the crude product, which was purified by column chromatography (eluent hexan/EtOAc 97:3, Rf = 0.32) to give acrylate **10b** as clear oil (328 mg, 94%). ^{1}H NMR: δ 1.79 (t, *J* = 2.5 Hz, 6H, C**H**$_3$), 2.50-2.68 (m, 4H, C-C**H**$_2$), 5.01 (quint, *J* = 6.0 Hz, 1H, CH$_2$-C**H**), 5.85 (dd, *J* = 10.0 Hz, *J* = 1.4 Hz, 1H, CH=C**H**$_2$), 6.15 (dd, *J* = 17.5 Hz, *J* = 10.4 Hz, 1H, C**H**=CH$_2$), 6.45 (dd, *J* = 17.3 Hz, *J* = 1.4 Hz, 1H, CH=C**H**$_2$). ^{13}C NMR: δ 3.5 (s, 2C, **C**H$_3$), 23.2 (s, 2C, CH-**C**H$_2$), 71.0 (s, 1C, **C**H-CH$_2$), 73.4 (s, 1C, **C**≡C-CH$_3$), 78.1 (s, 1C, C≡**C**-CH$_3$), 128.5 (s, 1C, **C**H=CH$_2$), 131.0 (s, 1C, CH=**C**H$_2$), 165.4 (s, 1C, **C**=O). MS (ESI$^+$), *m/z* (%): 213.1 [M+Na]$^+$ (100). HRMS (ESI$^+$): calcd. for $C_{12}H_{15}O_2$ ([M+H]$^+$) 191.1067, found 191.1065. HRMS (ESI$^+$): calcd. for $C_{12}H_{14}O_2Na$ ([M+Na]$^+$) 213.0886, found 213.0885.

Trideca-4,9-diyn-7-yl acrylate (10d)

Trideca-4,9-diyn-7-ol (**4d**, 192 mg, 1.00 mmol) and triethylamine (202 mg, 2.00 mmol) were dissolved in dry CH_2Cl_2 (3.5 mL) and the mixture was cooled to 0 °C. Freshly distilled acryloyl chloride (136 mg, 1.50 mmol) was added dropwise and the mixture was stirred for 90 min at 0 °C. After quenching with brine (2.5 mL), the layers were separated, the aqueous phase extracted with Et_2O (2 × 3.5 mL), and the combined organic layers were dried over Na_2SO_4. Careful evaporation gave the crude product, which was purified by column chromatography (eluent hexan/EtOAc 55:1, Rf = 0.24) to give acrylate **10d** as clear oil (166 mg, 67%). ^{1}H NMR: δ 0.97 (t, *J* = 7.4 Hz, 6H, C**H**$_3$), 1.44-1.56 (m, 4H, C**H**$_2$-CH$_3$), 2.13 (tt, *J* = 7.0 Hz, *J* = 2.3 Hz, 4H, C**H**$_2$-CH$_2$-CH$_3$), 2.53-2.69 (m, 4H, CH-C**H**$_2$), 5.04 (quint, *J* = 5.9 Hz, 1H, C**H**-CH$_2$), 5.85 (dd, *J* = 10.5 Hz, *J* = 1.4 Hz, 1H, CH=C**H**$_2$), 6.15 (dd, *J* = 17.0 Hz, *J* = 10.5 Hz, 1H, C**H**=CH$_2$), 6.44 (dd, *J* = 17.0 Hz, *J* = 1.4 Hz, 1H, CH=C**H**$_2$). ^{13}C NMR: δ 13.4 (s, 2C, **C**H$_3$), 20.7 (s, 2C, **C**H$_2$-CH$_2$-CH$_3$), 22.3 (s, 2C, CH$_2$-**C**H$_2$-CH$_3$), 23.3 (s, 2C, CH-**C**H$_2$), 71.0 (s, 1C, **C**H-CH$_2$), 74.9 (s, 1C, **C**≡C-CH$_2$-CH$_2$), 82.6 (s, 1C, C≡**C**-CH$_2$-CH$_2$), 128.5 (s, 1C, **C**H=CH$_2$), 130.9 (s, 1C, CH=**C**H$_2$), 165.4 (s, 1C, **C**=O). MS (EI$^+$), *m/z* (%): 145.1 $[M-C_5H_9O_2]^+$ (96), 131.1 $[M-C_6H_{11}O_2]^+$ (58), 117.1 $[M-C_7H_{13}O_2]^+$ (80), 91.0 $[C_7H_7]^+$ (48), 55.0 $[C_3H_3O]^+$ (100). HRMS (EI$^+$): calcd. for $C_{16}H_{22}O_2$ ($[M]^+$) 246.1620, found 246.1622.

Nona-2,7-diyn-5-yl methacrylate (11b)

Triethylamine (559 mg, 5.52 mmol) and 4-(dimethylamino)pyridine (34 mg, 0.28 mmol) were slowly added to a solution of nona-2,7-diyn-5-ol (**4b**, 250 mg, 1.84 mmol) in dry CH_2Cl_2 (5 mL) at 0 °C. Methacryloyl chloride (385 mg, 3.68 mmol) was added dropwise, the mixture was allowed to warm to rt, and stirred for 24 h. After quenching with saturated NH_4Cl (5 mL), the layers were separated, the aqueous

phase was extracted with Et_2O (2 × 5 mL), and the combined organic layers were dried over anhydr. $MgSO_4$. Evaporation and purification by column chromatography (eluent hexan/EtOAc 97:3, Rf = 0.43) gave methacrylate **11b** as clear oil (161 mg, 43%). ^{1}H NMR: δ 1.78 (t, *J* = 2.6 Hz, 6H, C≡C-C**H**$_3$), 1.96 (s, 3H, C**H**$_3$), 2.49-2.67 (m, 4H, C-C**H**$_2$), 4.97 (quint, *J* = 5.9 Hz, 1H, CH$_2$-C**H**), 5.57-5.60 (m, 1H, C=C**H**$_2$), 6.14-6.16 (m, 1H, C=C**H**$_2$). ^{13}C NMR: δ 3.2 (s, 2C, C≡C-**C**H$_3$), 17.9 (s, 1C, **C**H$_3$), 22.8 (s, 2C, C-**C**H$_2$), 70.8 (s, 1C, **C**H), 73.6 (s, 1C, **C**≡C-CH$_3$), 77.6 (s, 1C, C≡**C**-CH$_3$), 125.4 (s, 1C, C=**C**H$_2$), 136.0 (s, 1C, **C**=CH$_2$), 166.3 (s, 1C, **C**=O). MS (ESI$^+$), *m/z* (%): 227.1 $[M+Na]^+$ (100). HRMS (ESI$^+$): calcd. for $C_{13}H_{17}O_2$ ($[M+H]^+$) 205.1223, found 205.1224. HRMS (ESI$^+$): calcd. for $C_{13}H_{16}O_2Na$ ($[M+Na]^+$) 227.1043, found 227.1044.

Trideca-4,9-diyn-7-yl methacrylate (11d)

Triethylamine (405 mg, 4.00 mmol) and 4-(dimethylamino)pyridine (24 mg, 0.20 mmol) were slowly added to a solution of trideca-4,9-diyn-7-ol (**4d**, 192 mg, 1.00 mmol) in dry CH_2Cl_2 (3 mL) at 0 °C. Methacryloyl chloride (209 mg, 2.00 mmol) was added dropwise, the mixture was allowed to warm to rt, and stirred for 20 h. After quenching with saturated NH_4Cl (3 mL), the layers were separated, the aqueous phase was extracted with Et_2O (2 × 3 mL), and the combined organic layers were dried over anhydr. $MgSO_4$. Careful evaporation and purification by column chromatography (eluent hexan/EtOAc 55:1, Rf = 0.23) gave methacrylate **11d** as clear oil (234 mg, 90%). ^{1}H NMR: δ 0.96 (t, *J* = 7.3 Hz, 6H, CH$_2$-C**H**$_3$), 1.43-1.56 (m, 4H, C**H**$_2$-CH$_3$), 1.96 (s, 3H, C**H**$_3$), 2.12 (tt, *J* = 7.0 Hz, *J* = 2.3 Hz, 4H, C**H**$_2$-CH$_2$-CH$_3$), 2.53-2.69 (m, 4H, CH-C**H**$_2$), 5.01 (quint, *J* = 5.6 Hz, 1H, CH$_2$-C**H**), 5.56-5.59 (m, 1H, C=C**H**$_2$), 6.15 (s, 1H, C=C**H**$_2$). ^{13}C NMR: δ 13.4 (s, 2C, CH$_2$-**C**H$_3$), 18.2 (s, 1C, **C**H$_3$), 20.7 (s, 2C, **C**H$_2$-CH$_2$-CH$_3$), 22.3 (s, 2C, CH$_2$-**C**H$_2$-CH$_3$), 23.4 (s, 2C, CH-**C**H$_2$), 71.1

(s, 1C, **C**H-CH$_2$), 75.0 (s, 2C, **C**≡C-CH$_2$-CH$_2$), 82.5 (s, 2C, C≡**C**-CH$_2$-CH$_2$), 125.6 (s, 1C, C=**C**H$_2$), 136.3 (s, 1C, **C**=CH$_2$), 166.6 (s, 1C, **C**=O). MS (EI$^+$), *m/z* (%): 174.1 [M-M-$C_4H_6O_2$]$^+$ (62), 159.1 [M-$C_5H_9O_2$]$^+$ (55), 145.1 [M-$C_6H_{11}O_2$]$^+$ (100), 131.1 [M-$C_7H_{13}O_2$]$^+$ (80), 117.1 [M-$C_8H_{15}O_2$]$^+$ (95), 91.0 [C_7H_7]$^+$ (70). HRMS (EI$^+$): calcd. for $C_{17}H_{24}O_2$ ([M]$^+$) 260.1776, found 260.1775.

2. Copies of ^{1}H and ^{13}C NMR spectra

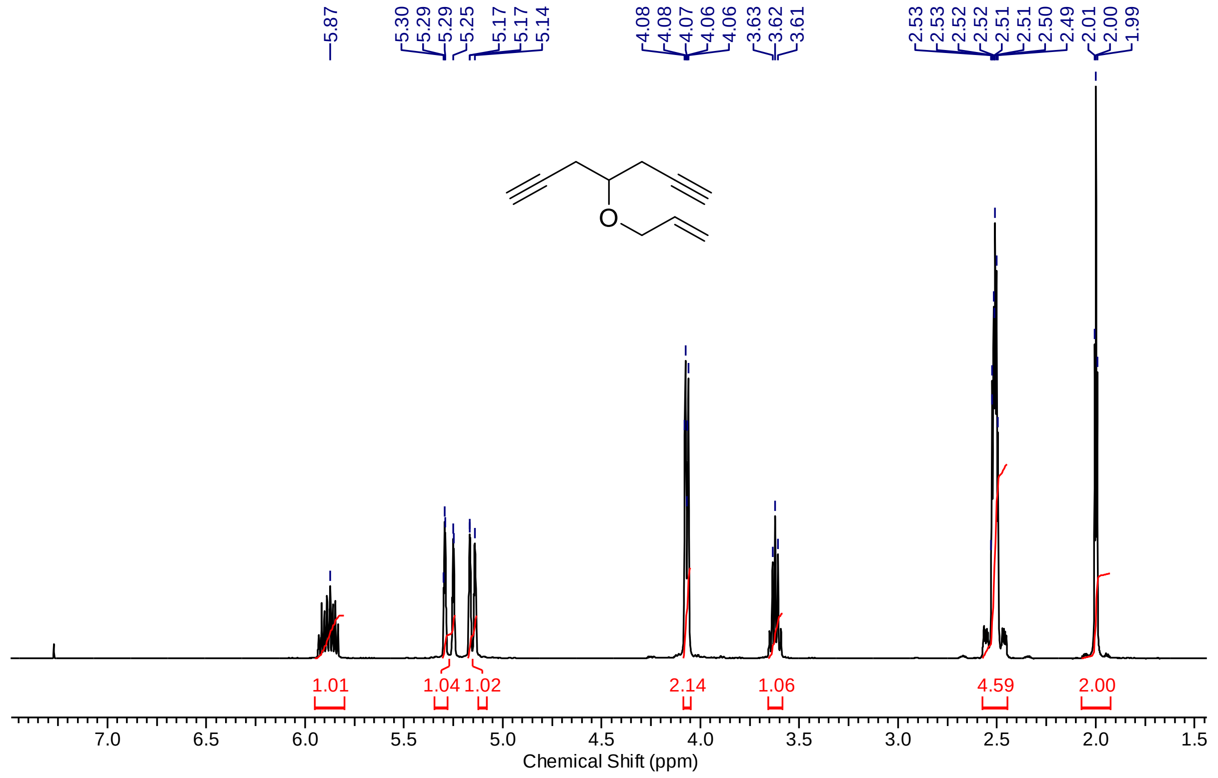

Figure S1. ^{1}H NMR spectrum, 299.97 MHz, $CDCl_3$, 4-(allyloxy)hepta-1,6-diyne (**2a**).

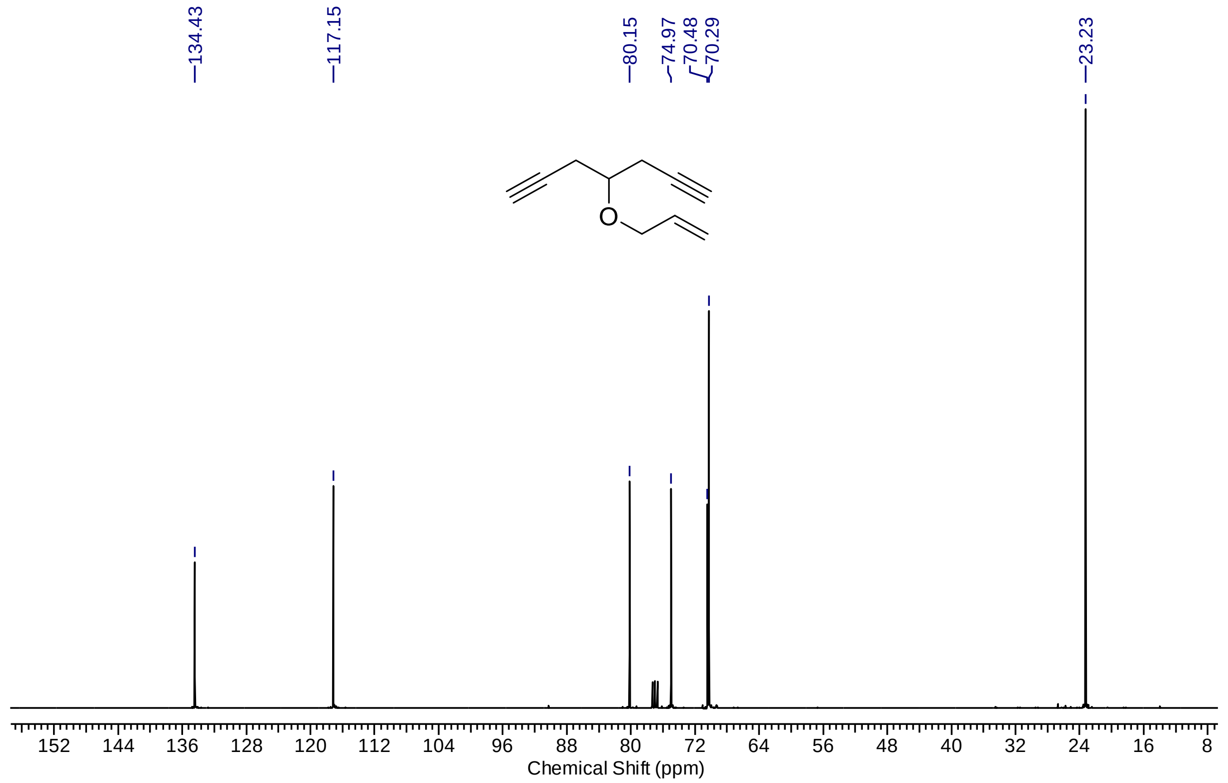

Figure S2. $^{13}C\{1H\}$ NMR spectrum, 75.44 MHz, $CDCl_3$, 4-(allyloxy)hepta-1,6-diyne (**2a**).

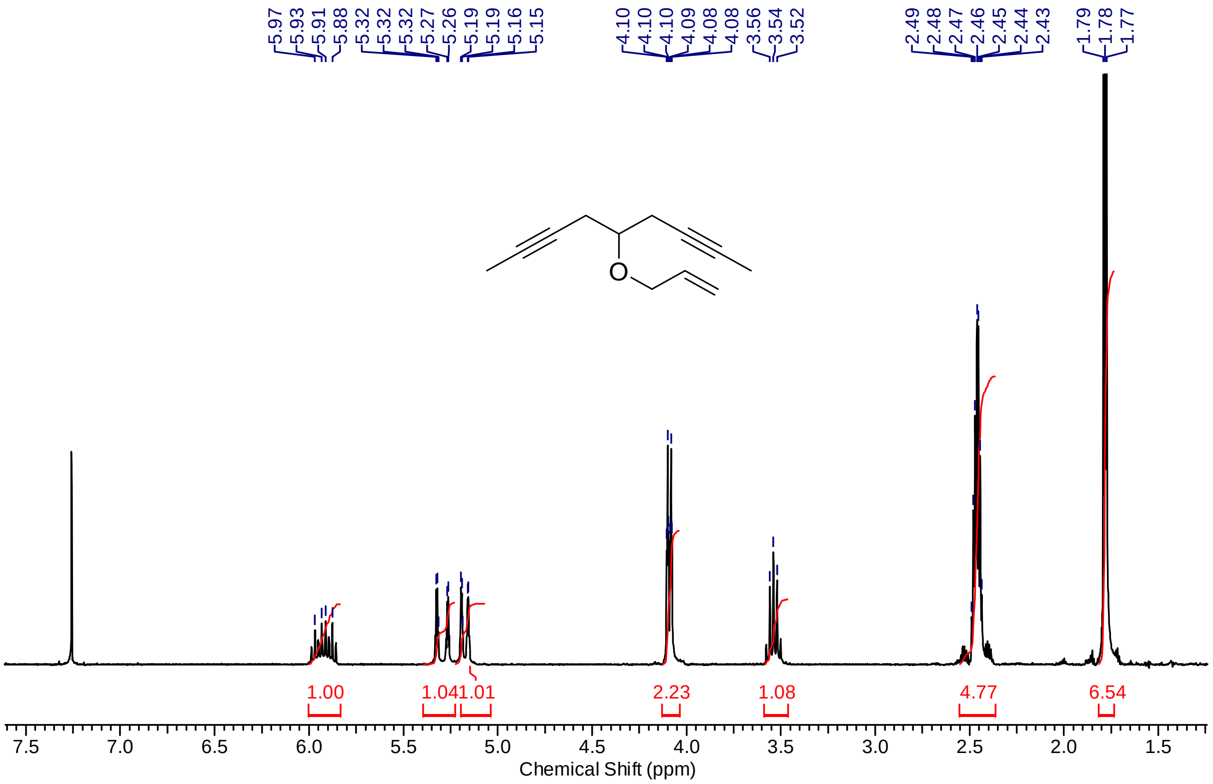

Figure S3. ^{1}H NMR spectrum, 299.97 MHz, CDCl3, 5-(allyloxy)nona-2,7-diyne (**2b**).

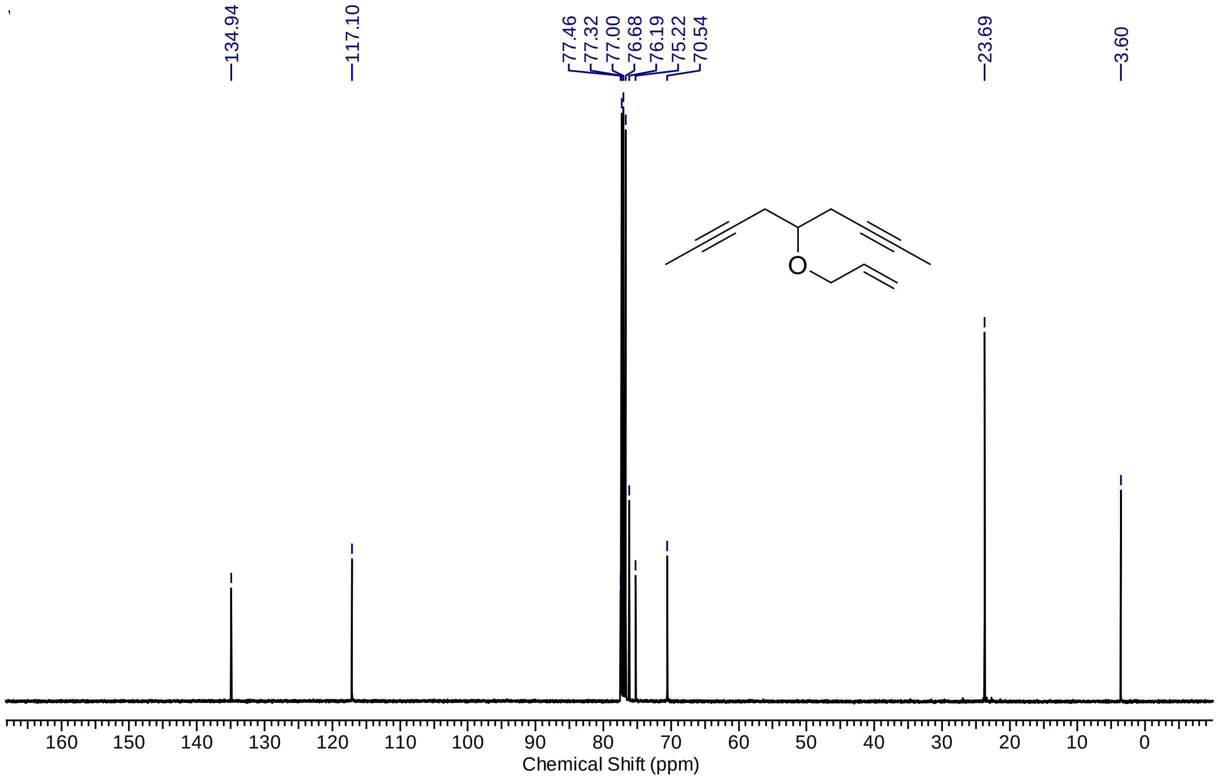

Figure S4. ^{13}C{1H} NMR spectrum, 75.44 MHz, CDCl3, 5-(allyloxy)nona-2,7-diyne (**2b**).

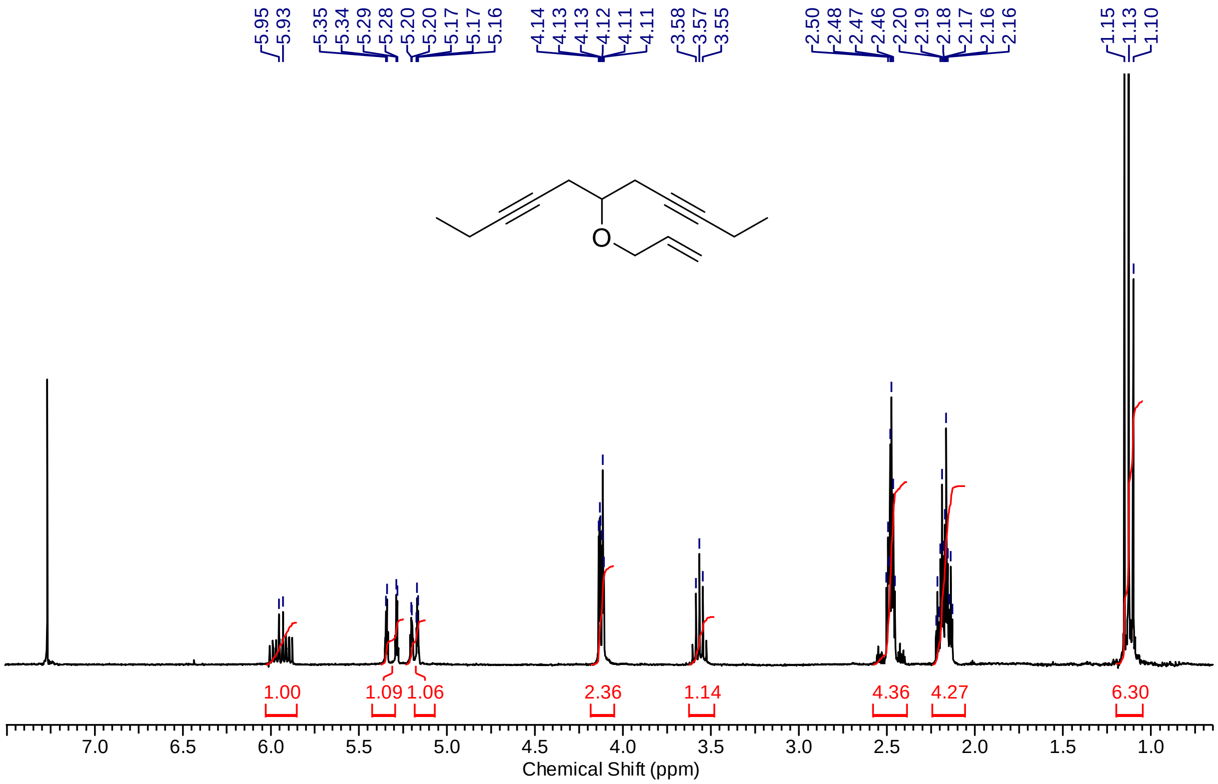

Figure S5. ^{1}H NMR spectrum, 299.97 MHz, $CDCl_3$, 6-(allyloxy)undeca-3,8-diyne (**2c**).

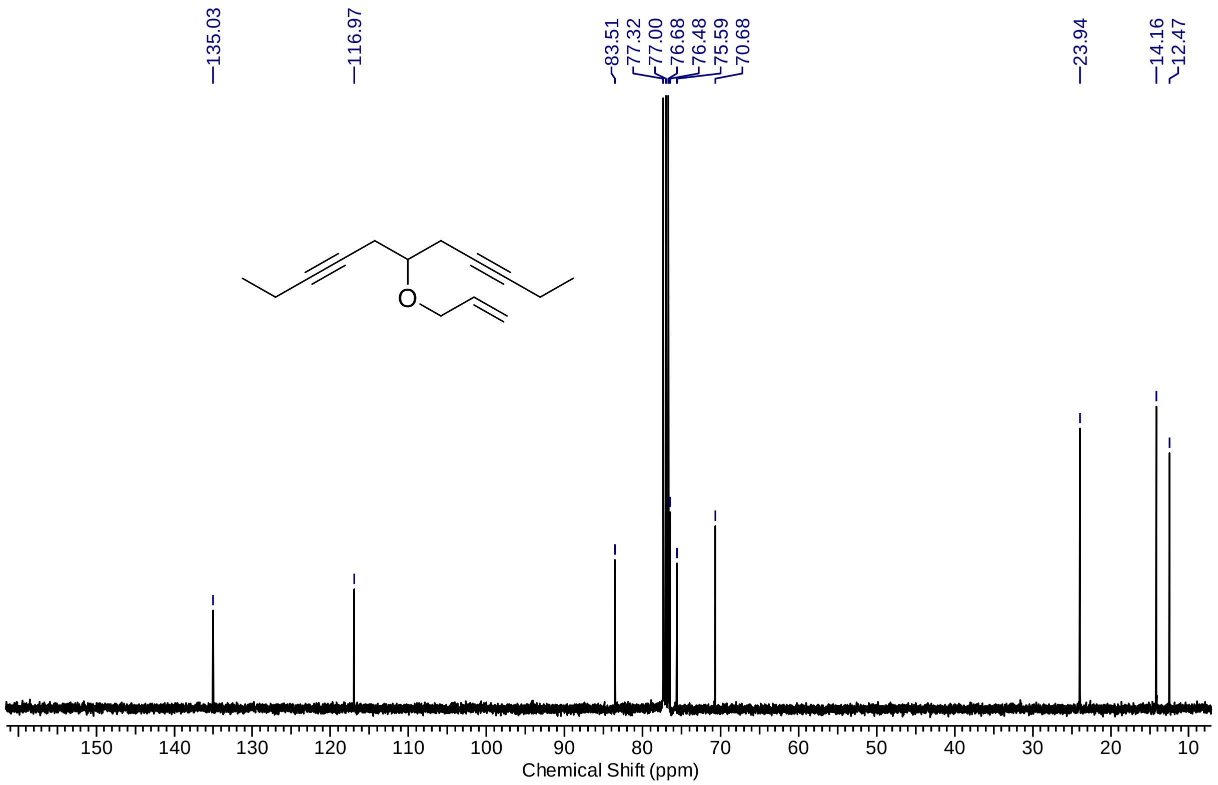

Figure S6. $^{13}C\{1H\}$ NMR spectrum, 75.44 MHz, $CDCl_3$, 6-(allyloxy)undeca-3,8-diyne (**2c**).

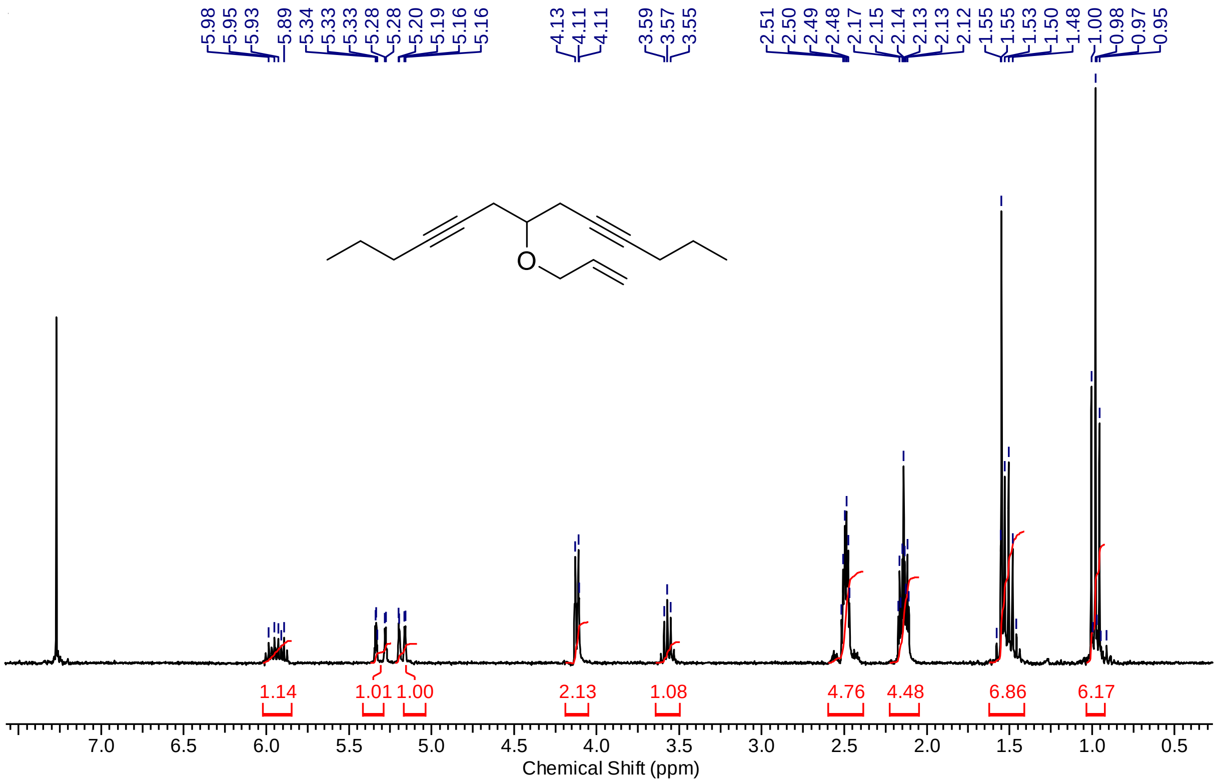

Figure S7. ^{1}H NMR spectrum, 299.97 MHz, $CDCl_3$, 7-(allyloxy)trideca-4,9-diyne (**2d**).

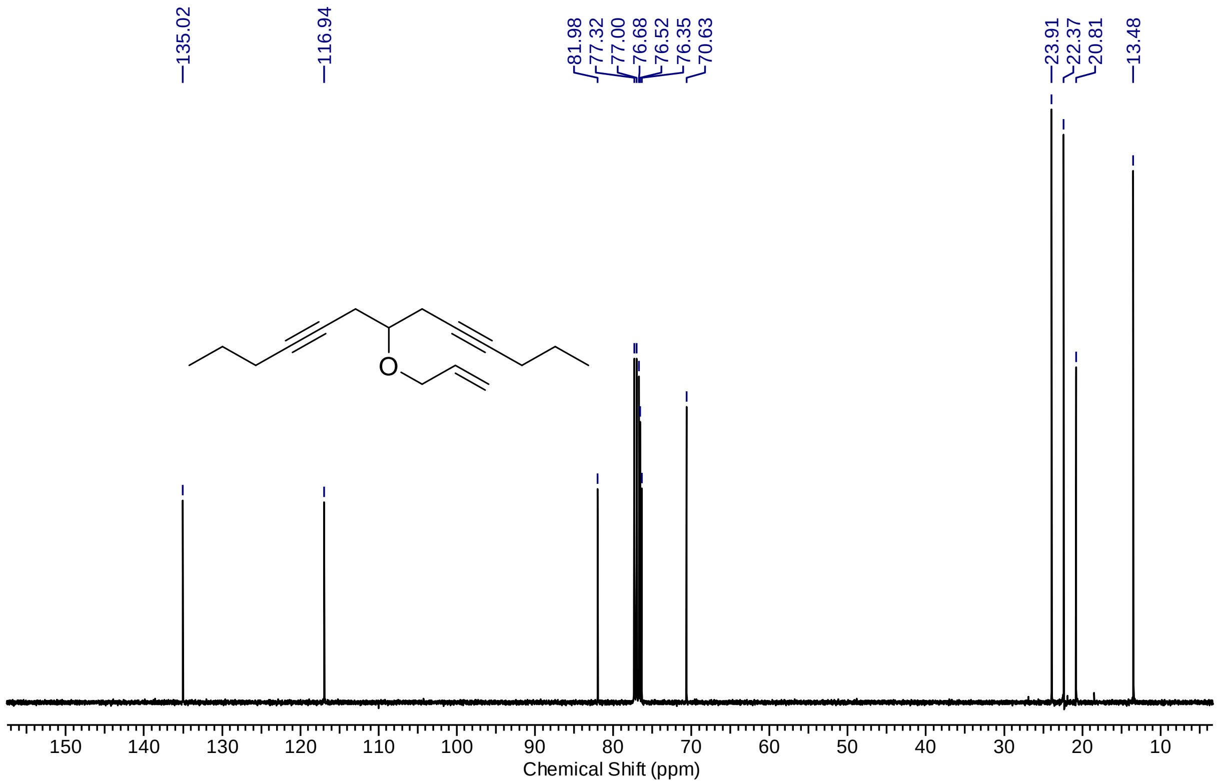

Figure S8. $^{13}C\{1H\}$ NMR spectrum, 75.44 MHz, $CDCl_3$, 7-(allyloxy)trideca-4,9-diyne (**2d**).

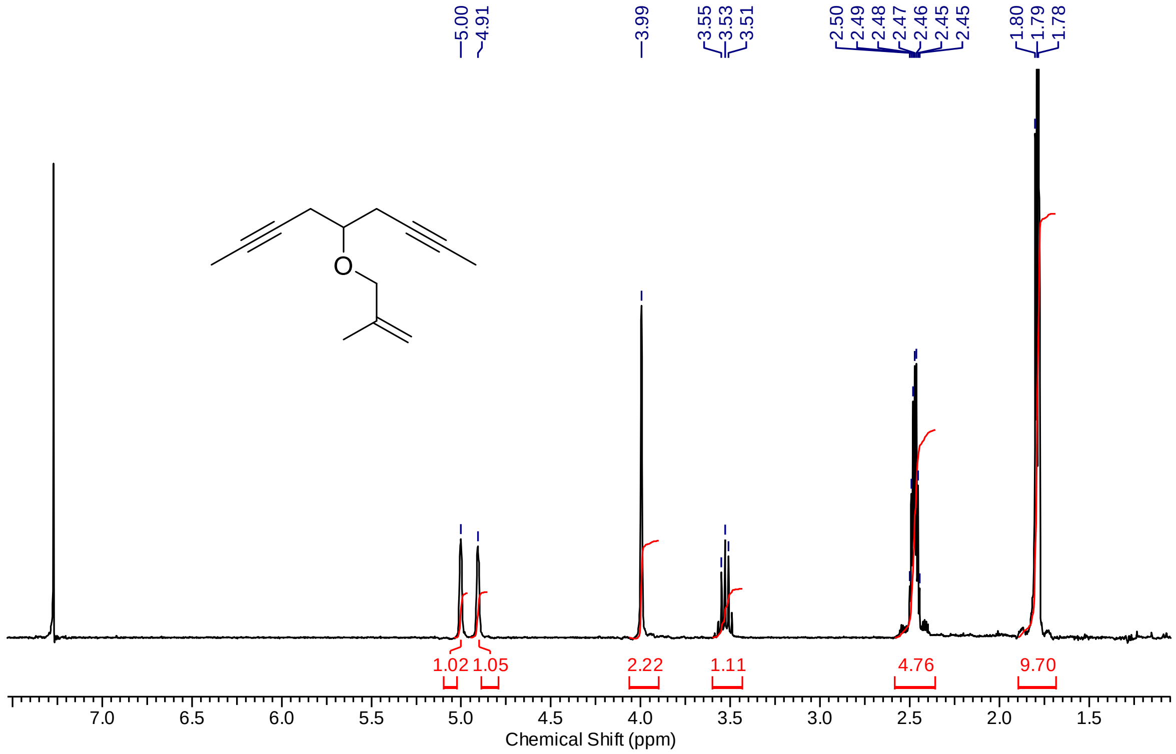

Figure S9. ^{1}H NMR spectrum, 299.97 MHz, $CDCl_3$, 5-((2-methylallyl)oxy)nona-2,7-diyne (**9b**).

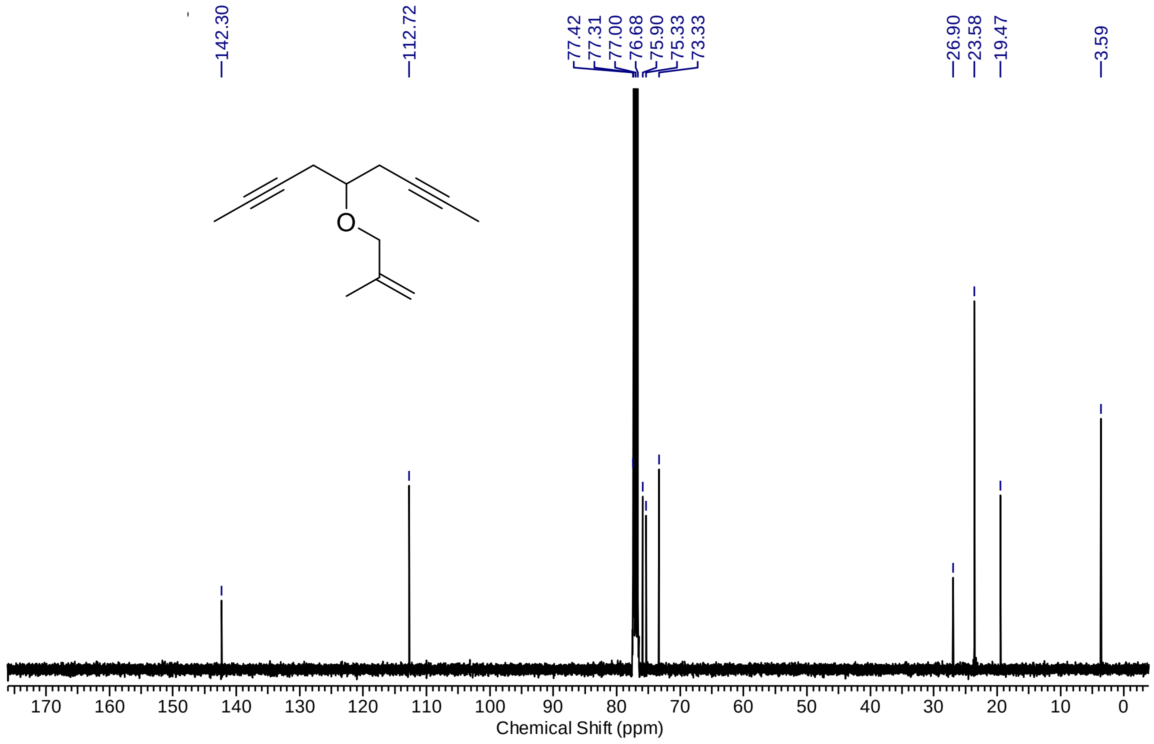

Figure S10. ^{13}C{1H} NMR spectrum, 75.44 MHz, $CDCl_3$, 5-((2-methylallyl)oxy)nona-2,7-diyne (**9d**).

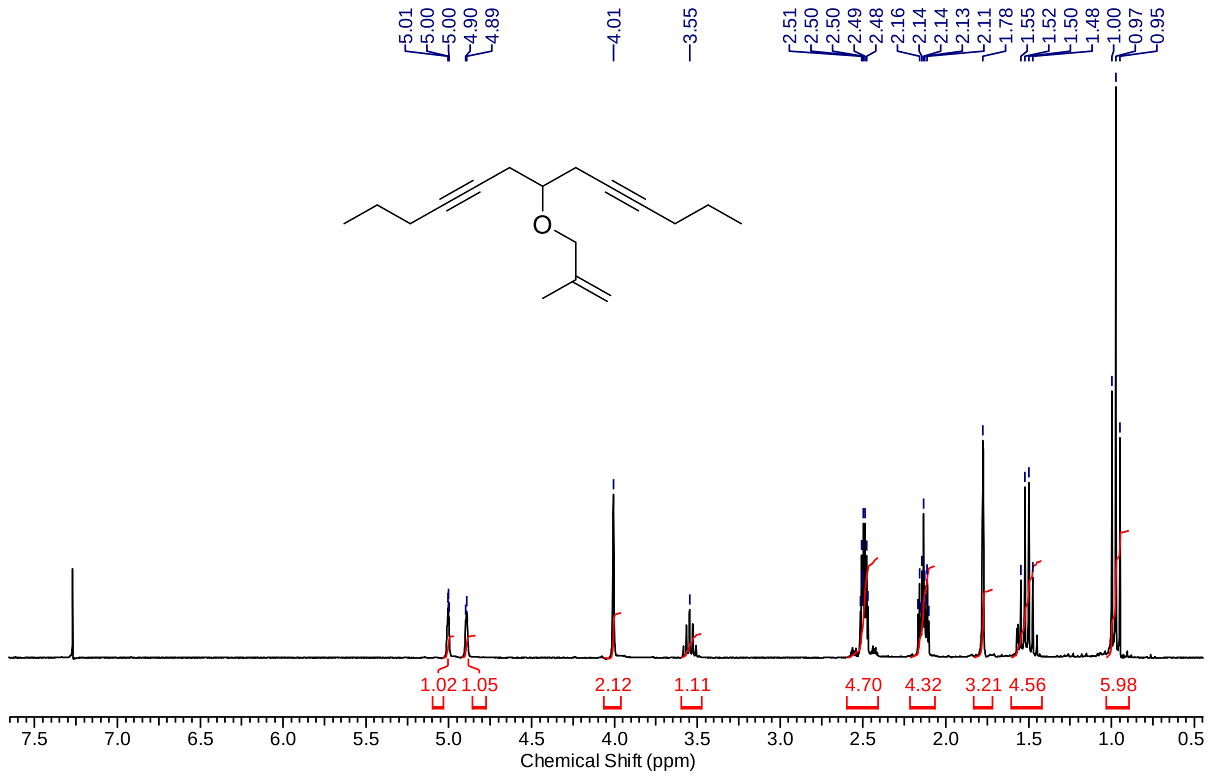

Figure S11. ^{1}H NMR spectrum, 299.97 MHz, $CDCl_3$, 7-((2-methylallyl)oxy)trideca-4,9-diyne (**9d**).

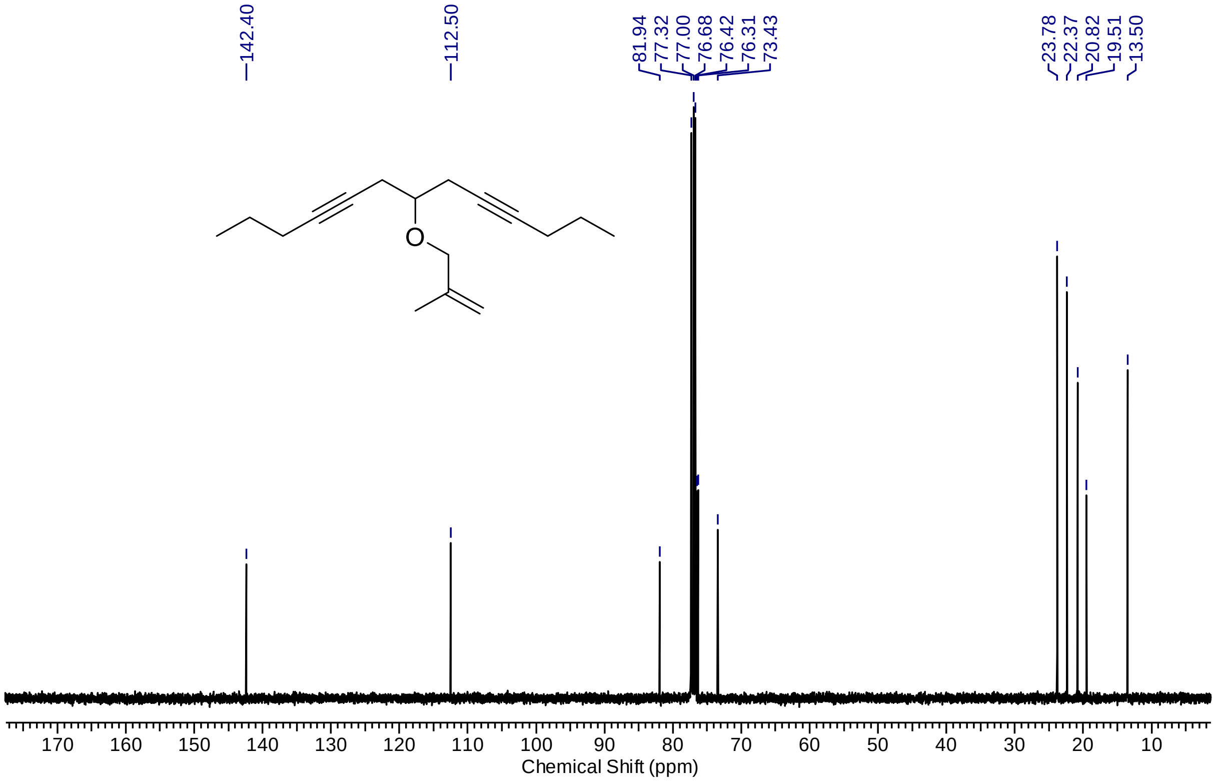

Figure S12. ^{13}C{1H} NMR spectrum, 75.44 MHz, $CDCl_3$, 7-((2-methylallyl)oxy)trideca-4,9-diyne (**9d**).

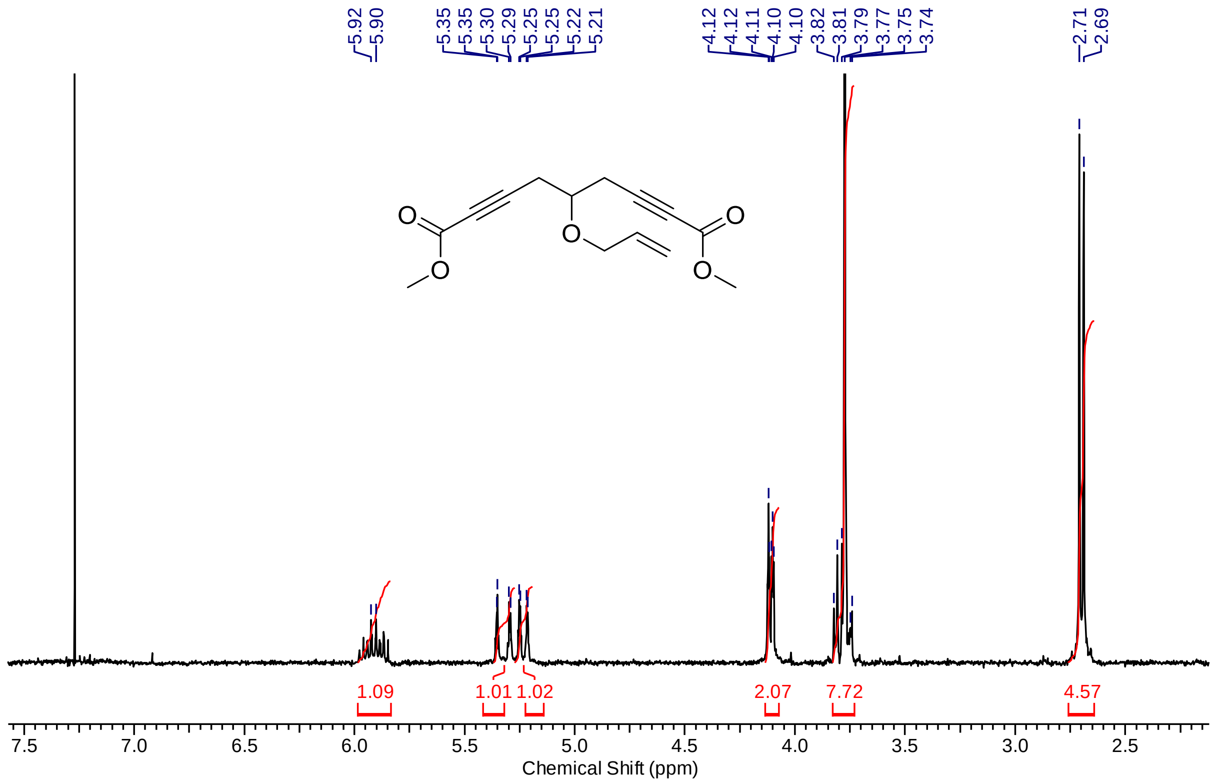

Figure S13. ^{1}H NMR spectrum, 299.97 MHz, $CDCl_3$, dimethyl 5-(allyloxy)nona-2,7-diynedioate (**2e**).

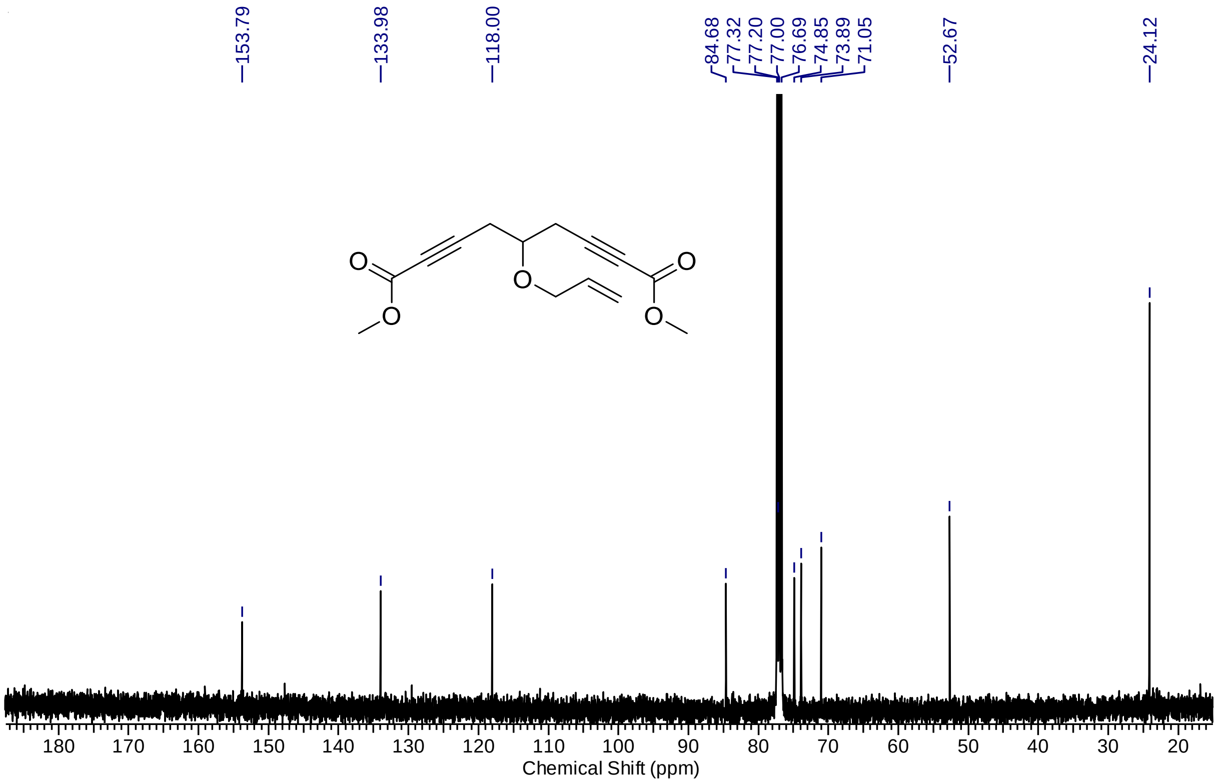

Figure S14. ^{13}C{1H} NMR spectrum, 75.44 MHz, $CDCl_3$, dimethyl 5-(allyloxy)nona-2,7-diynedioate (**2e**).

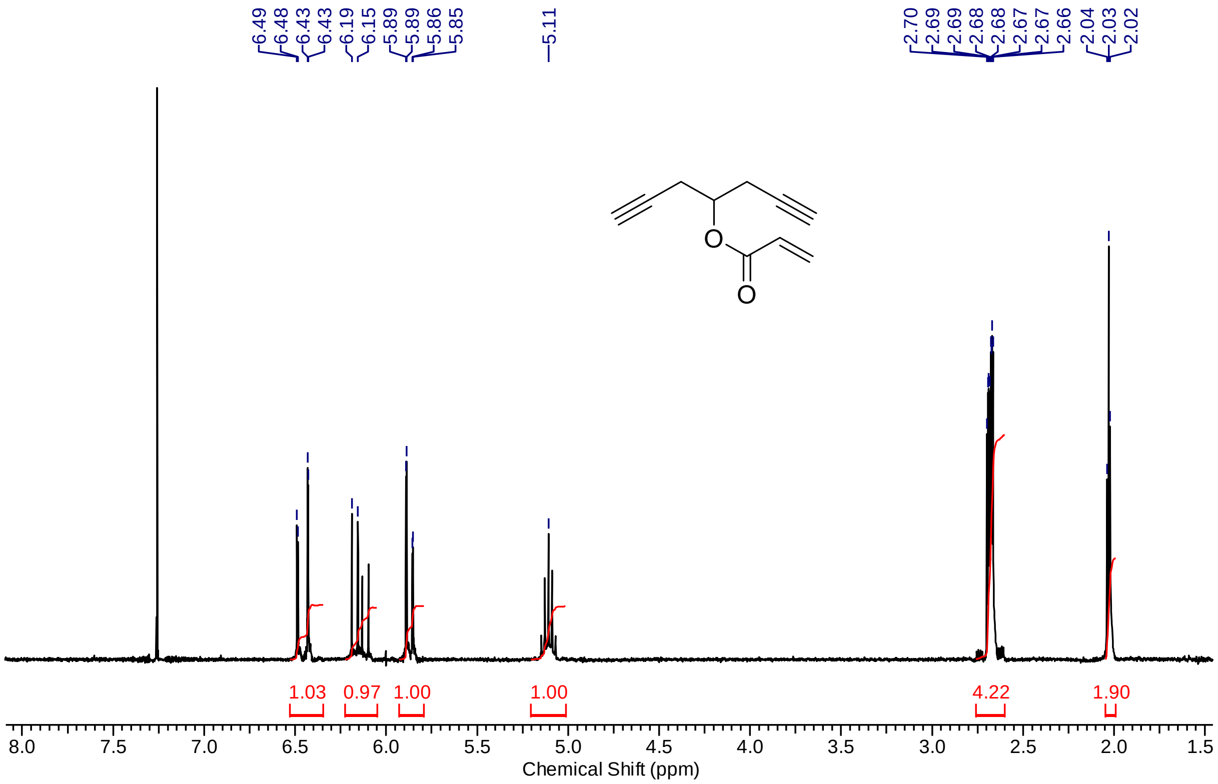

Figure S15. ^{1}H NMR spectrum, 299.97 MHz, $CDCl_3$, hepta-1,6-diyn-4-yl acrylate (**10a**).

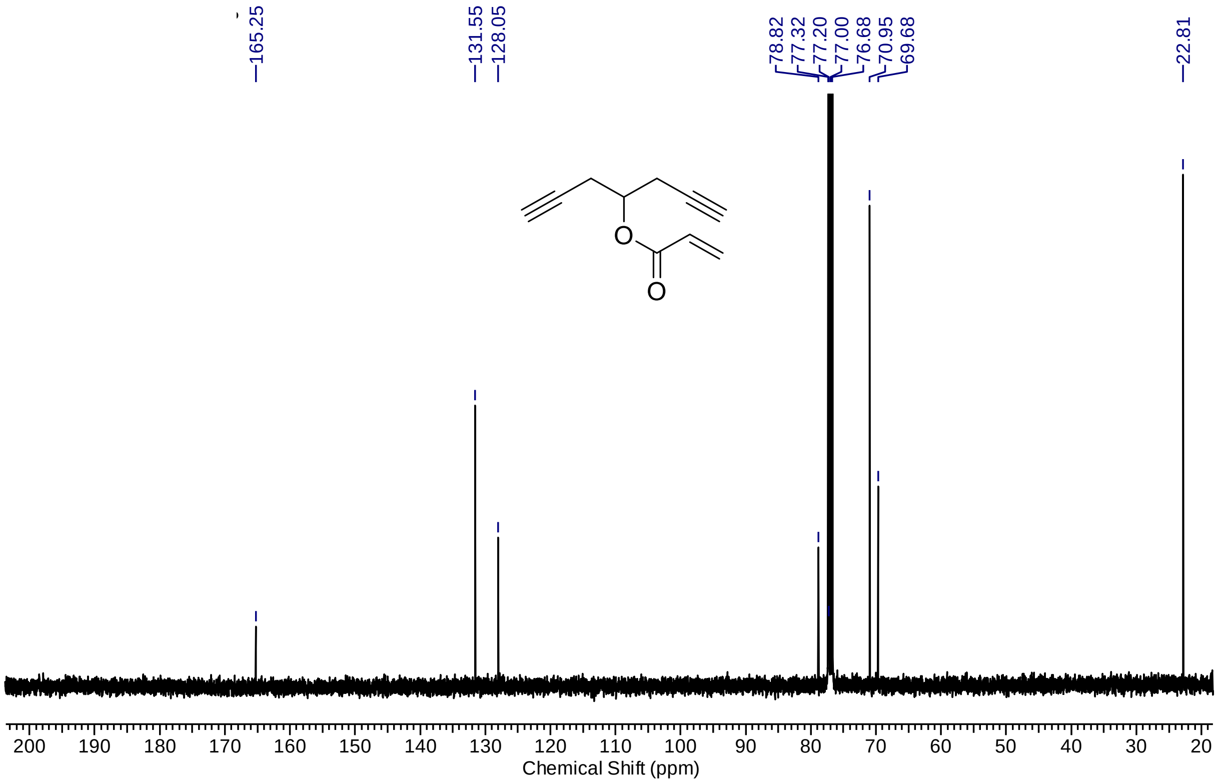

Figure S16. ^{13}C{1H} NMR spectrum, 75.44 MHz, $CDCl_3$, hepta-1,6-diyn-4-yl acrylate (**10a**).

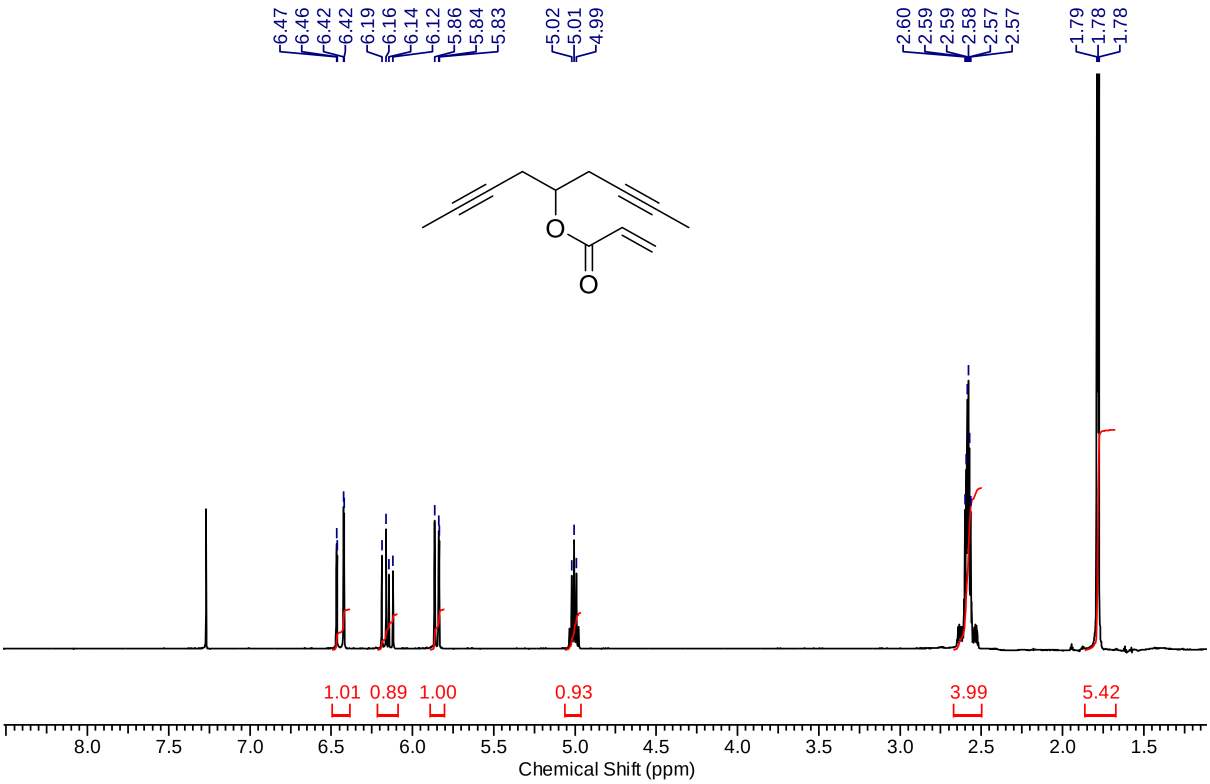

Figure S17. ¹H NMR spectrum, 299.97 MHz, CDCl₃, nona-2,7-diyn-5-yl acrylate (**10b**).

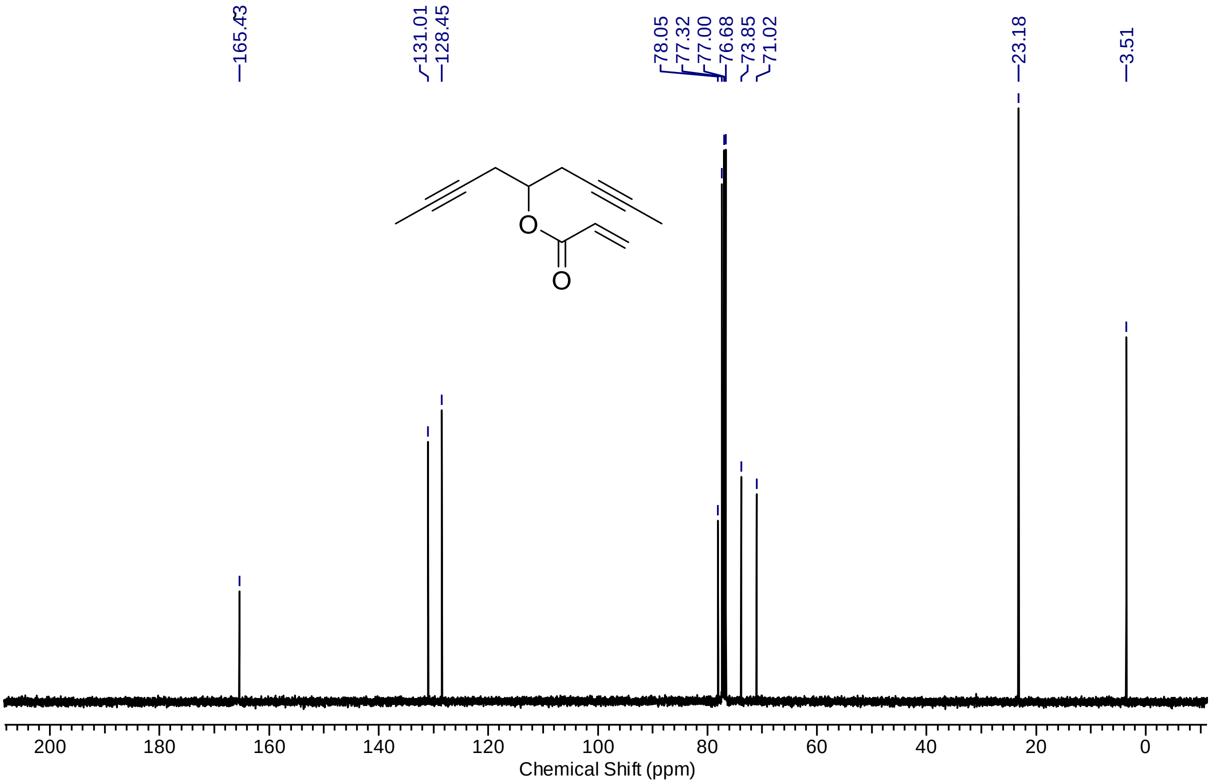

Figure S18. ¹³C{1H} NMR spectrum, 75.44 MHz, CDCl₃, nona-2,7-diyn-5-yl acrylate (**10b**).

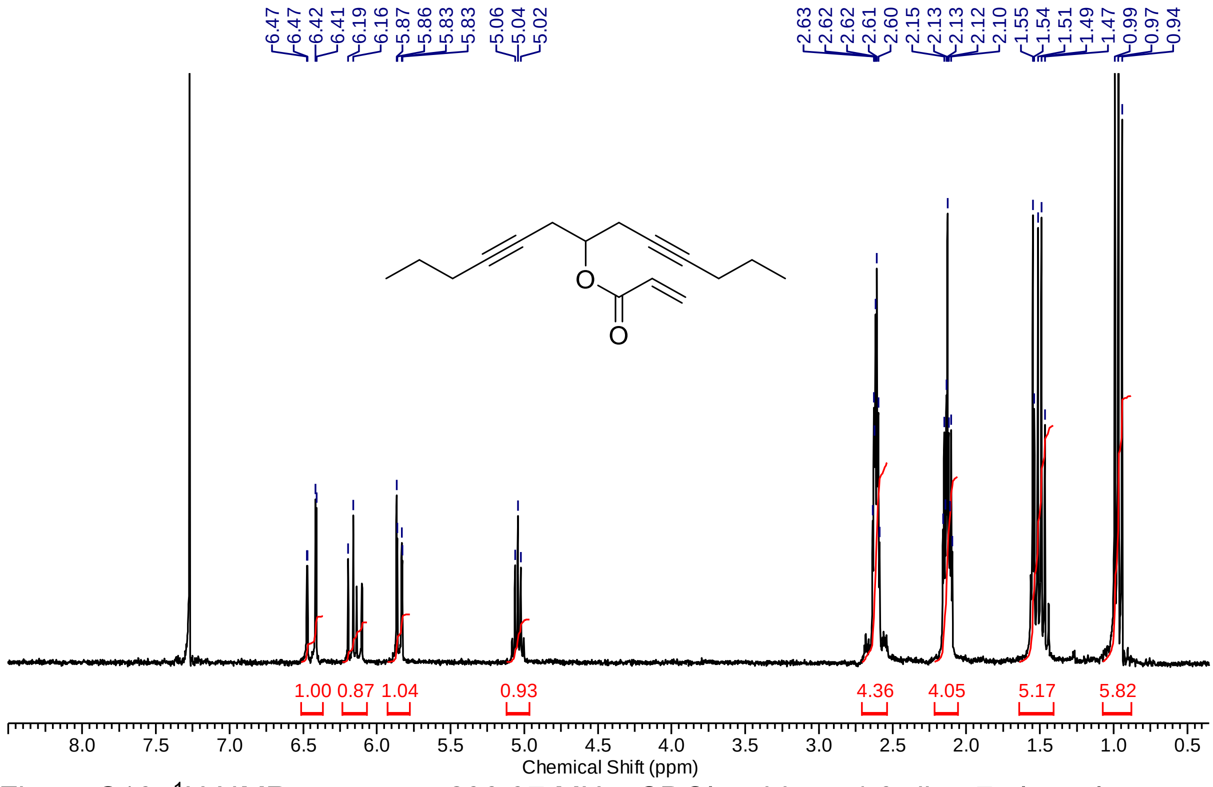

Figure S19. ^{1}H NMR spectrum, 299.97 MHz, CDCl3, trideca-4,9-diyn-7-yl acrylate (**10d**).

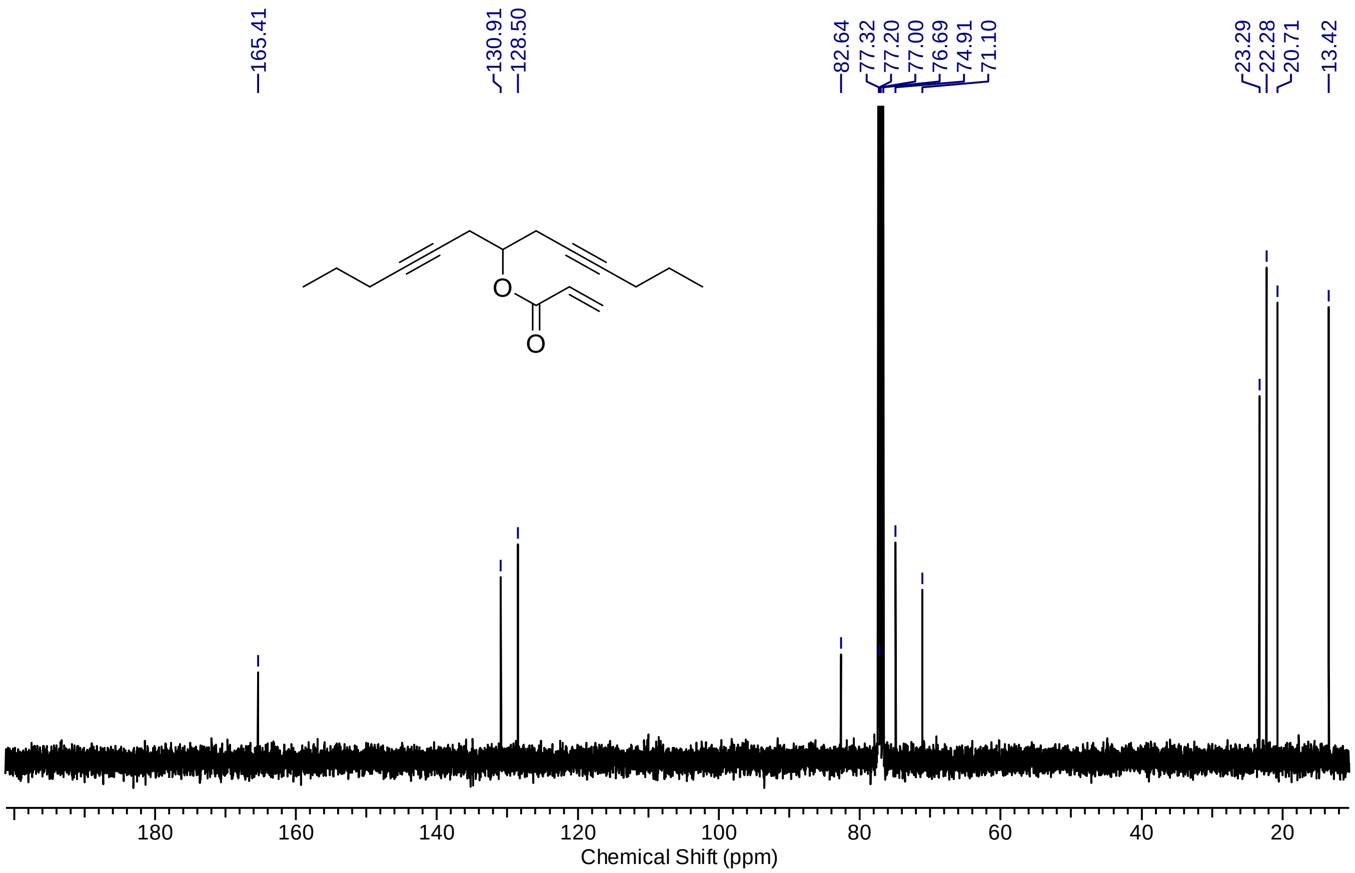

Figure 20. ^{13}C{1H} NMR spectrum, 75.44 MHz, CDCl3, trideca-4,9-diyn-7-yl acrylate (**10d**).

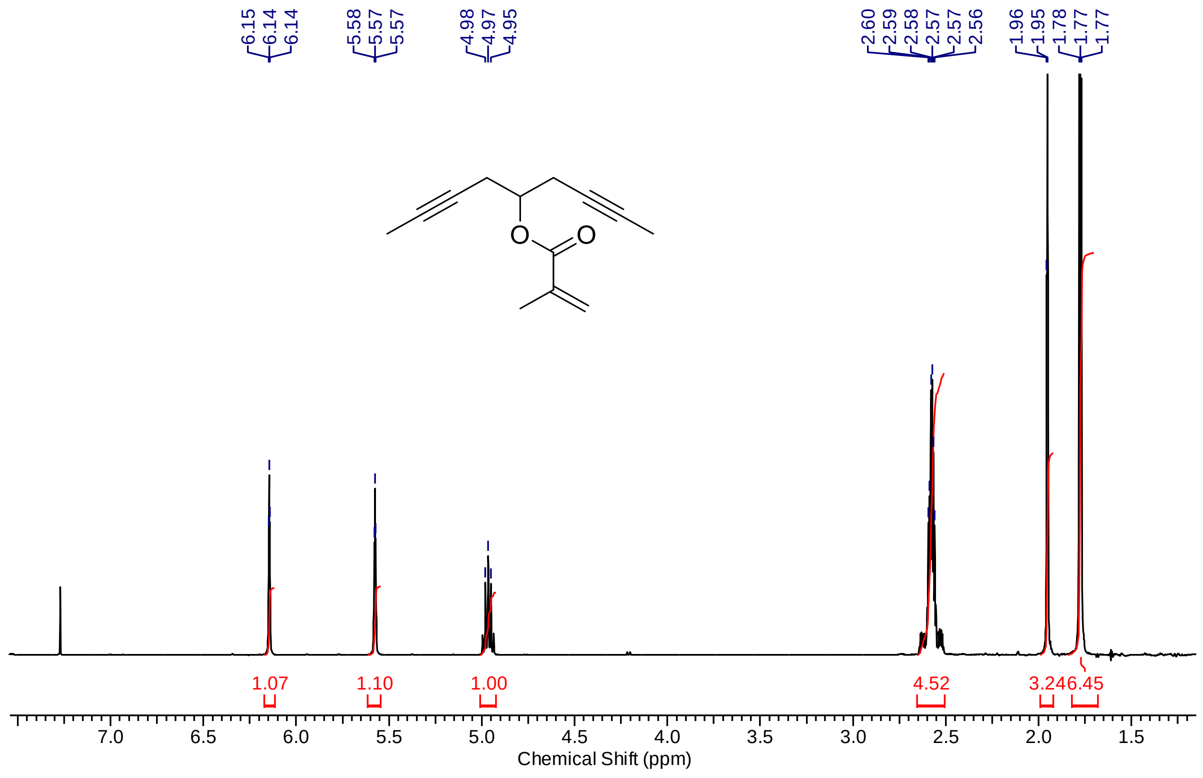

Figure S21. ^{1}H NMR spectrum, 299.97 MHz, $CDCl_3$, nona-2,7-diyn-5-yl methacrylate (**11b**).

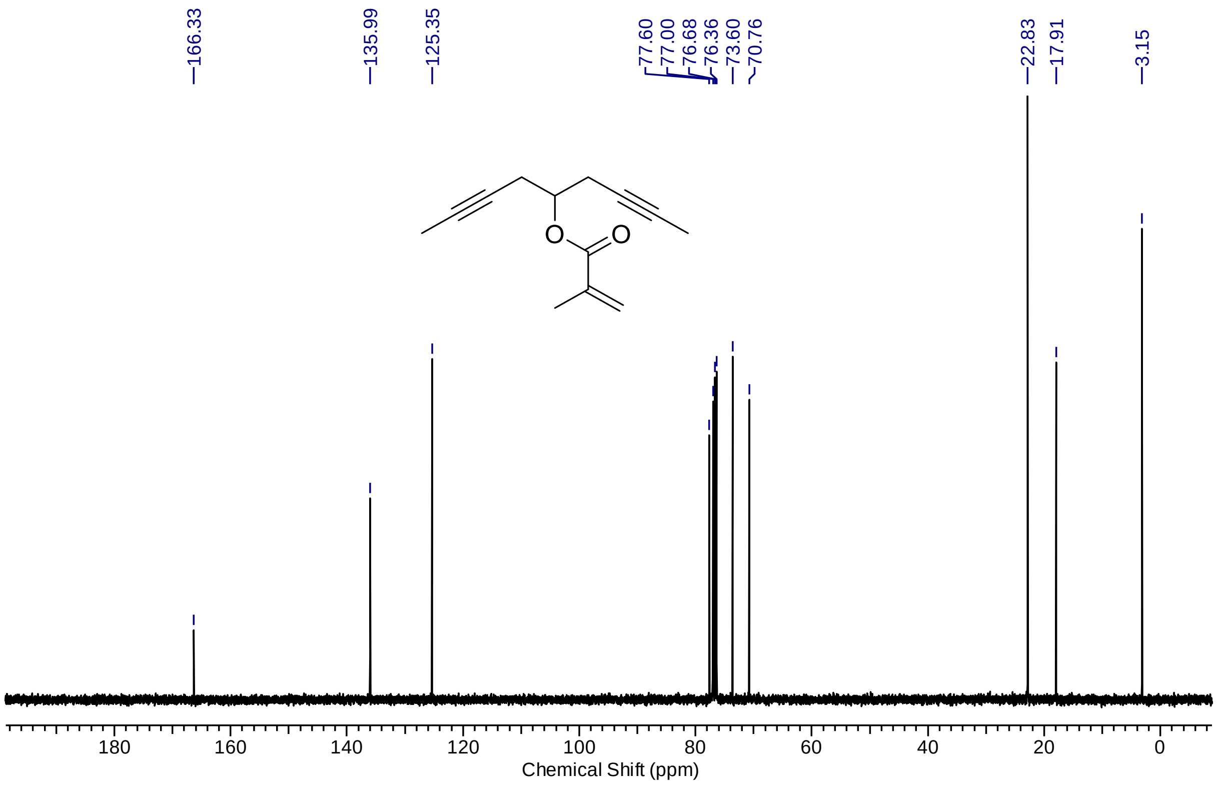

Figure S22. $^{13}C\{1H\}$ NMR spectrum, 75.44 MHz, $CDCl_3$, nona-2,7-diyn-5-yl methacrylate (**11b**).

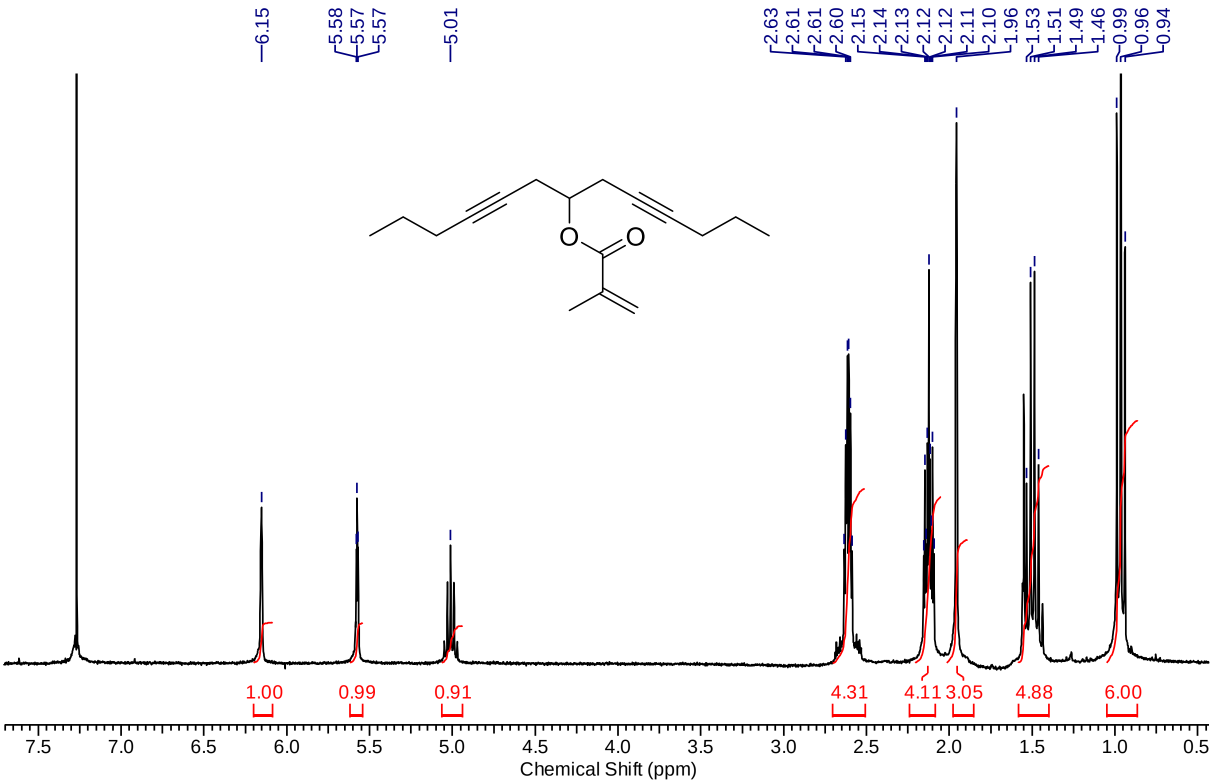

Figure S23. ^{1}H NMR spectrum, 299.97 MHz, $CDCl_3$, trideca-4,9-diyn-7-yl methacrylate (**11d**).

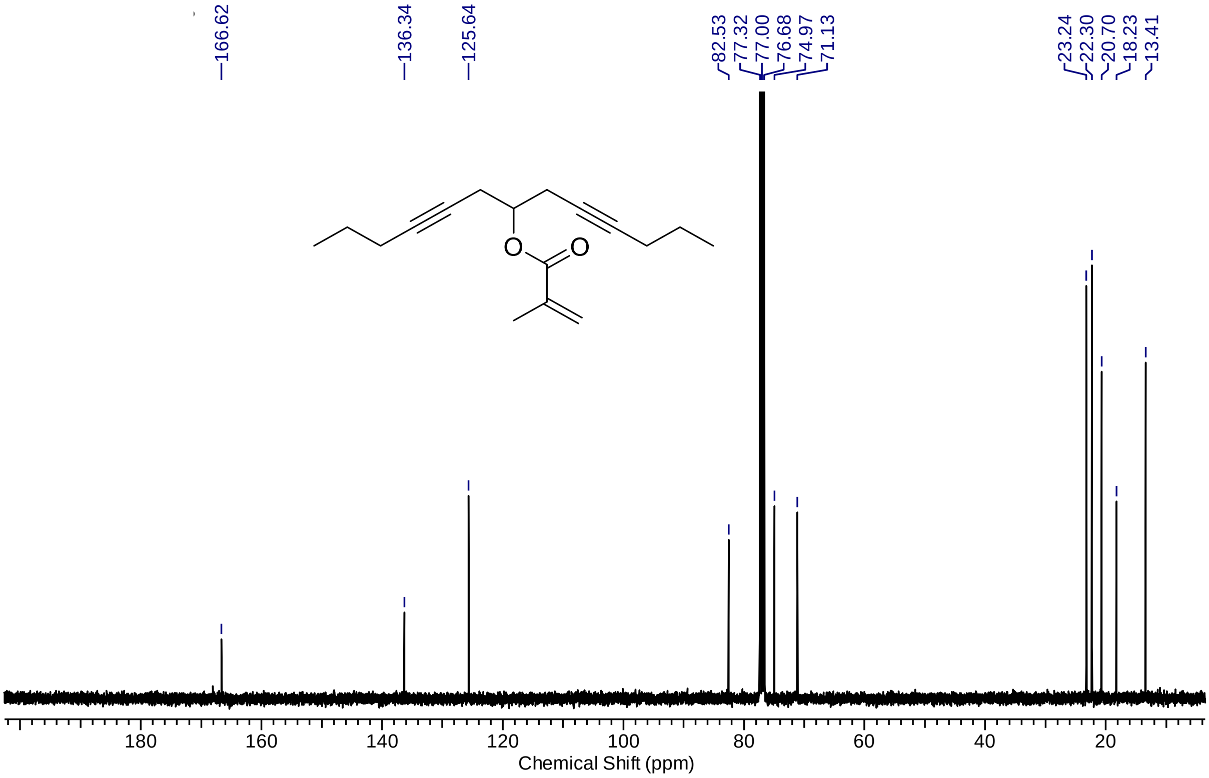

Figure S24. $^{13}C\{1H\}$ NMR spectrum, 75.44 MHz, $CDCl_3$, trideca-4,9-diyn-7-yl methacrylate (**11d**).

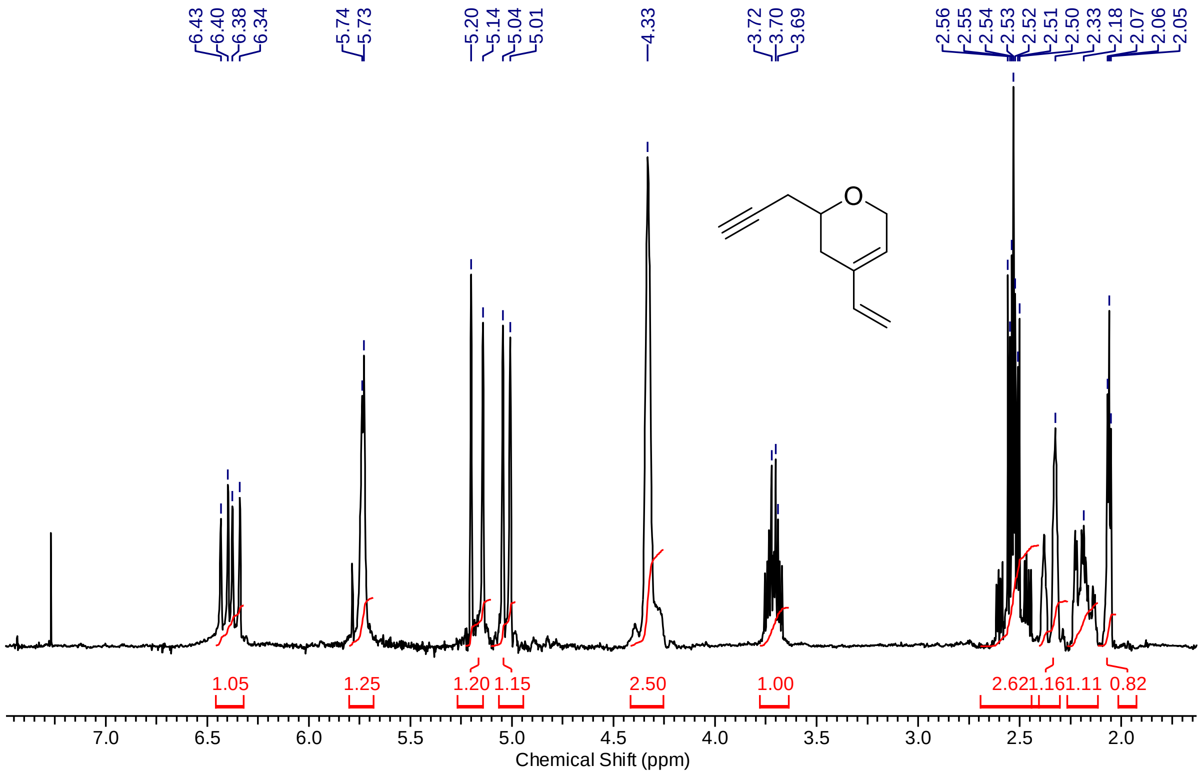

Figure S25. ^{1}H NMR spectrum, 299.97 MHz, $CDCl_3$, 4-ethenyl-2-prop-2-yn-1-yl-3,6-dihydro-*2H*-pyran (**12a**).

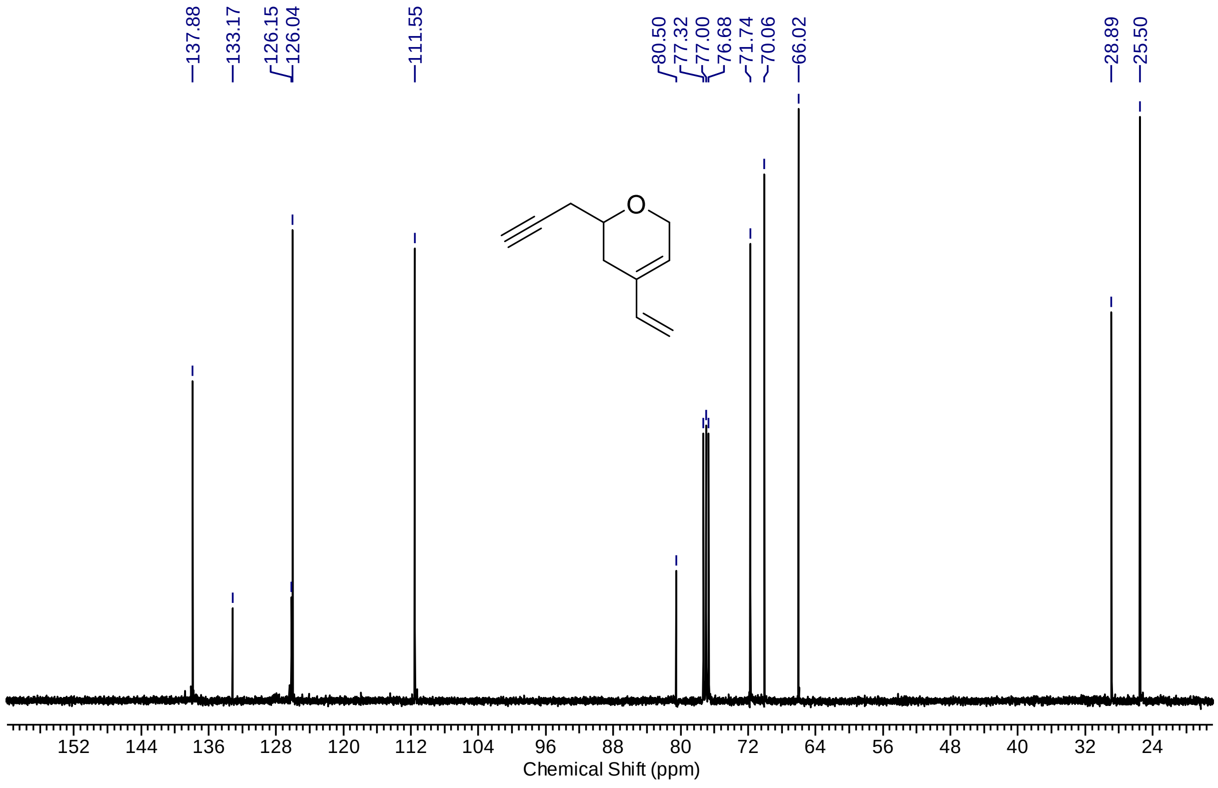

Figure S26. ^{13}C{1H} NMR spectrum, 75.44 MHz, $CDCl_3$, 4-ethenyl-2-prop-2-yn-1-yl-3,6-dihydro-*2H*-pyran (**12a**).

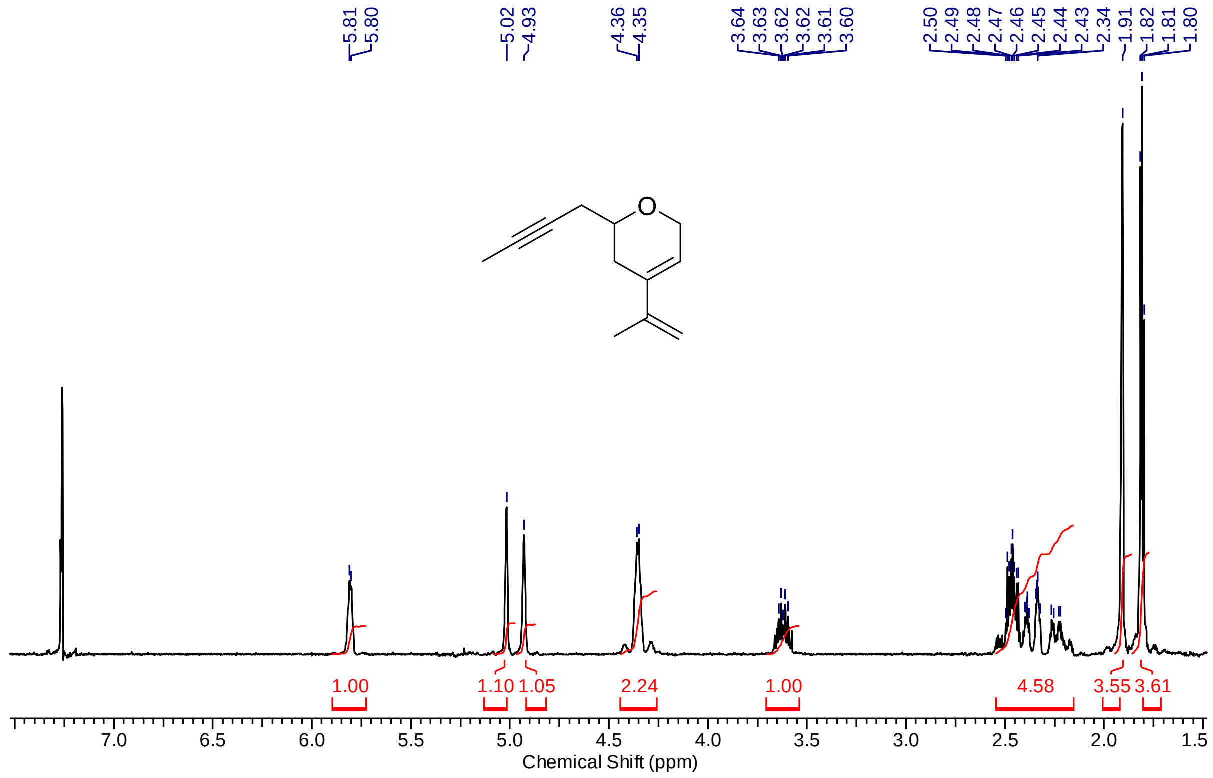

Figure S27. ^{1}H NMR spectrum, 299.97 MHz, $CDCl_3$, 2-(but-2-yn-1-yl)-4-(prop-1-en-2-yl)-3,6-dihydro-2*H*-pyran (**12b**).

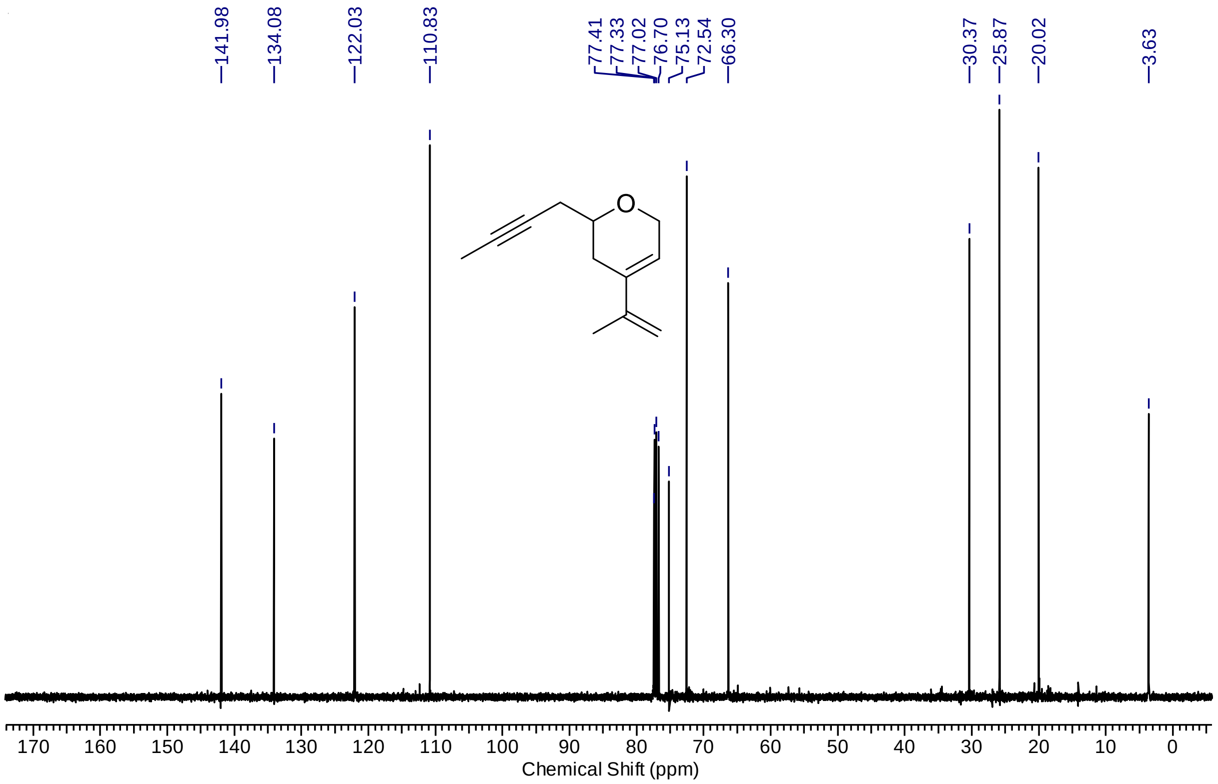

Figure S28. ^{13}C{1H} NMR spectrum, 75.44 MHz, $CDCl_3$, 2-(but-2-yn-1-yl)-4-(prop-1-en-2-yl)-3,6-dihydro-2*H*-pyran (**12b**).

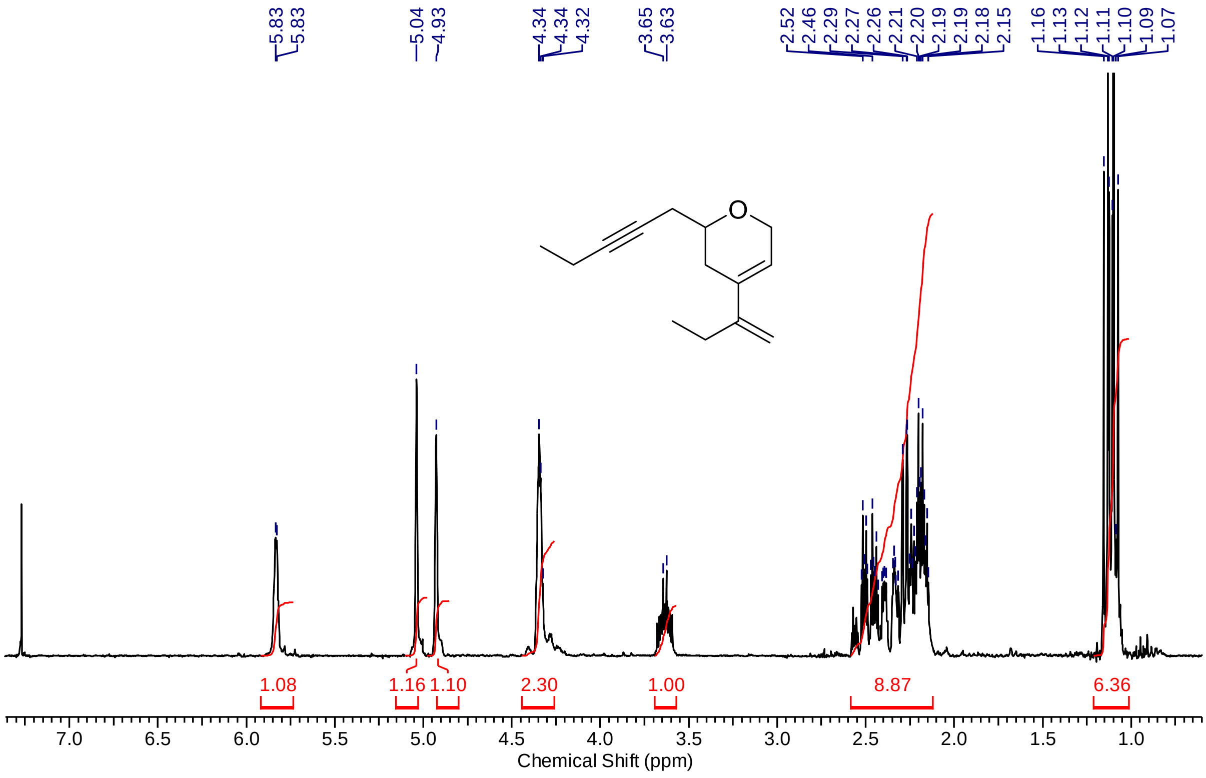

Figure S29. ^{1}H NMR spectrum, 299.97 MHz, $CDCl_3$, 4-(but-1-en-2-yl)-2-(pent-2-yn-1-yl)-3,6-dihydro-2*H*-pyran (**12c**).

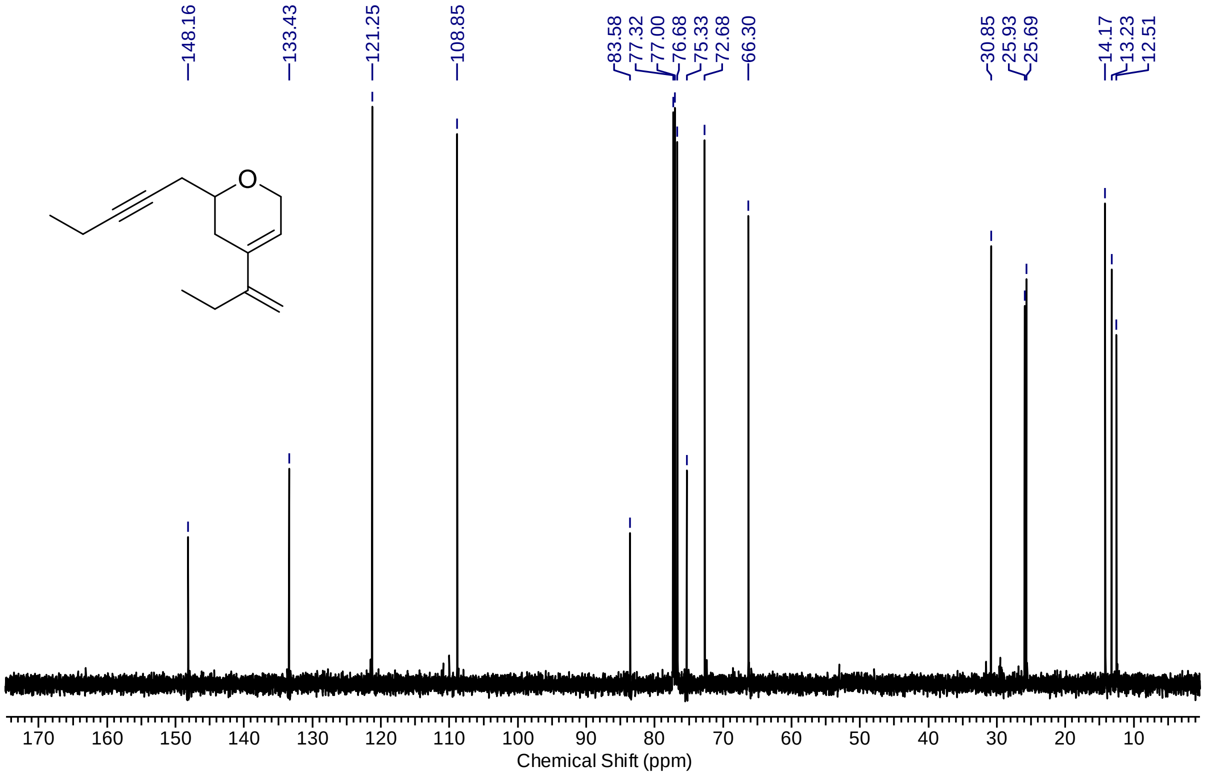

Figure S30. ^{13}C{1H} NMR spectrum, 75.44 MHz, $CDCl_3$, 4-(but-1-en-2-yl)-2-(pent-2-yn-1-yl)-3,6-dihydro-2*H*-pyran (**12c**).

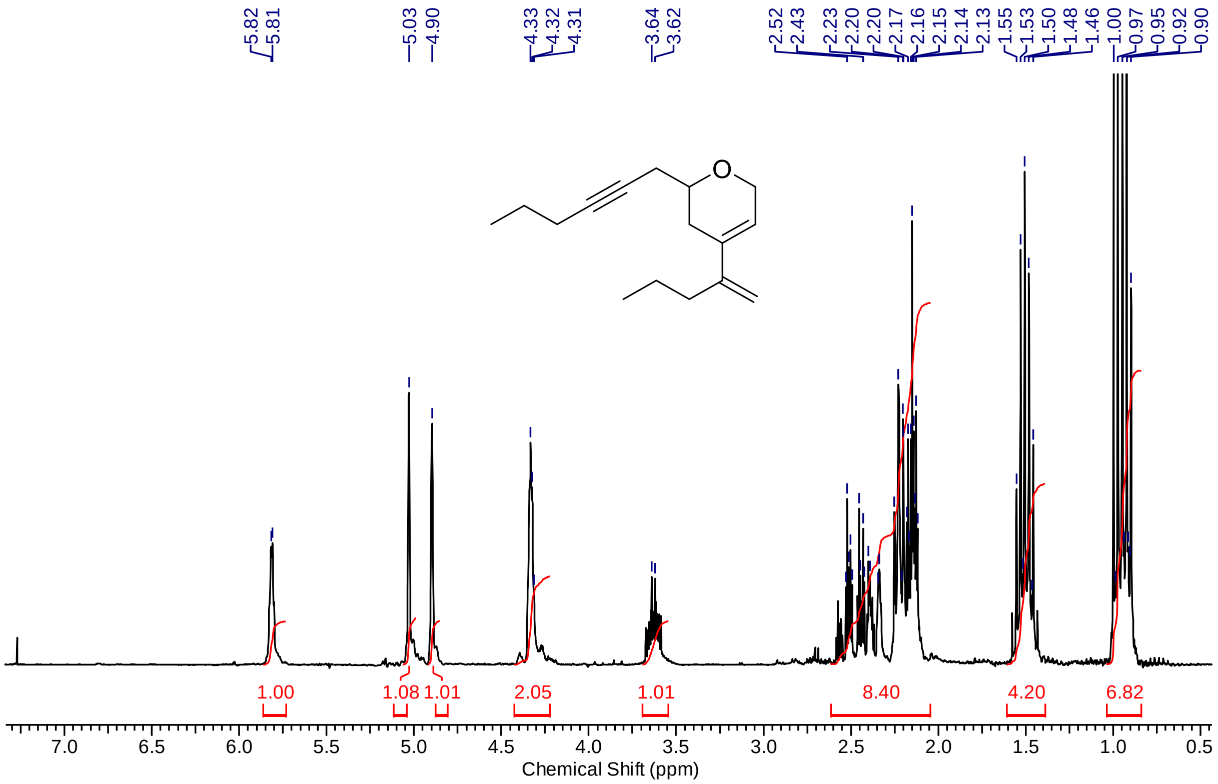

Figure S31. ^{1}H NMR spectrum, 299.97 MHz, $CDCl_3$, 2-(hex-2-yn-1-yl)-4-(pent-1-en-2-yl)-3,6-dihydro-2*H*-pyran (**12d**).

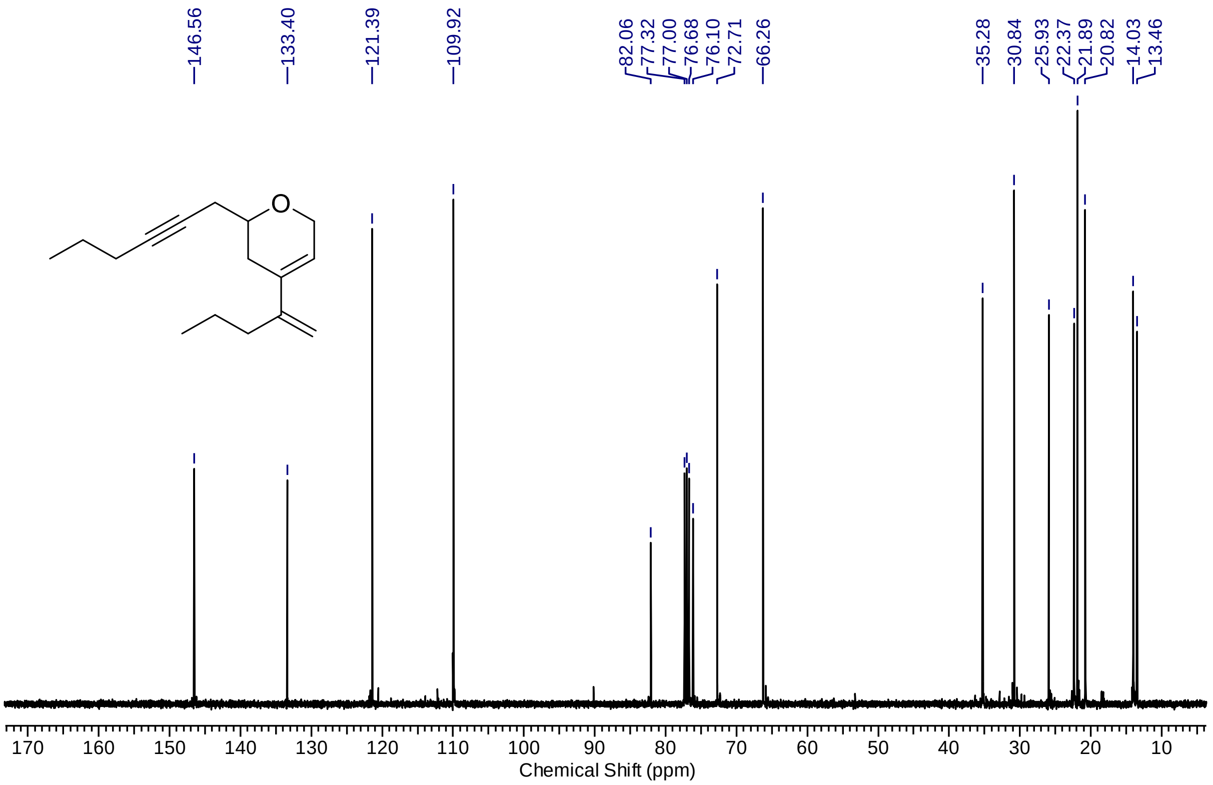

Figure S32. ^{13}C{1H} NMR spectrum, 75.44 MHz, $CDCl_3$, 2-(hex-2-yn-1-yl)-4-(pent-1-en-2-yl)-3,6-dihydro-2*H*-pyran (**12d**).

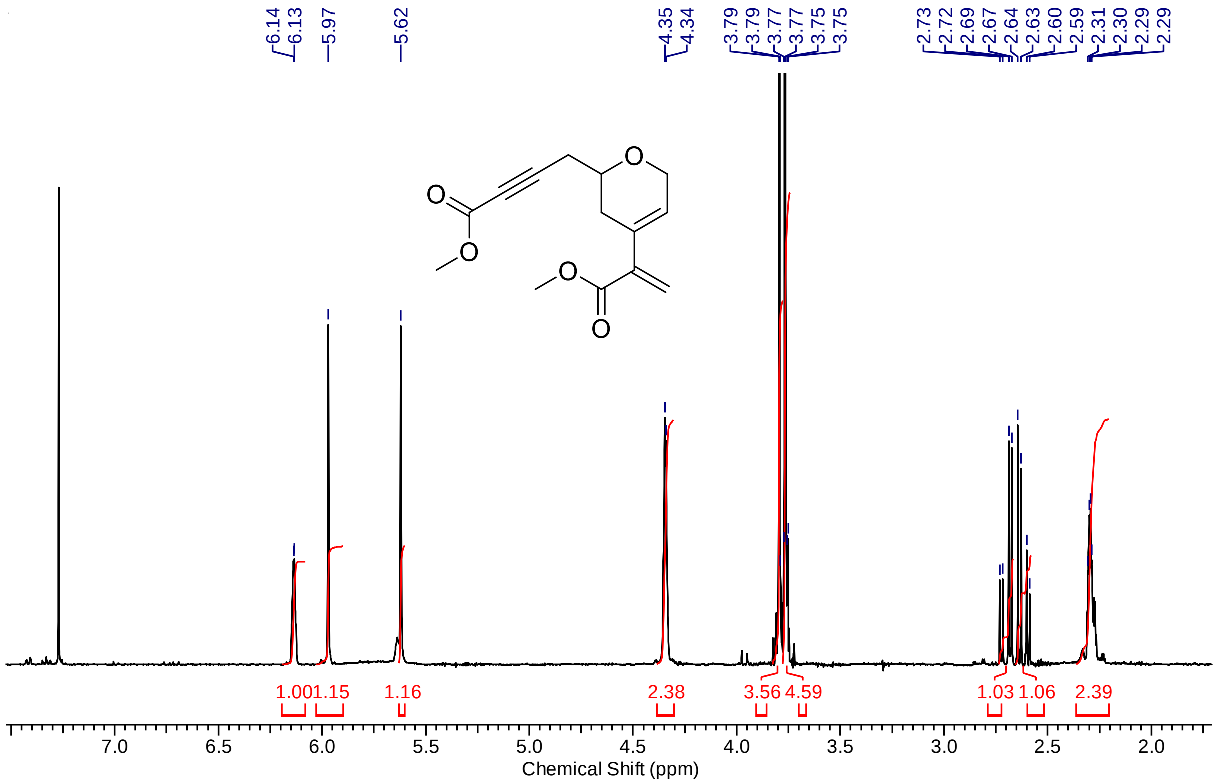

Figure S33. ¹H NMR spectrum, 299.97 MHz, CDCl₃, methyl 4-(4-(3-methoxy-3-oxoprop-1-en-2-yl)-3,6-dihydro-2*H*-pyran-2-yl)but-2-ynoate (**12e**).

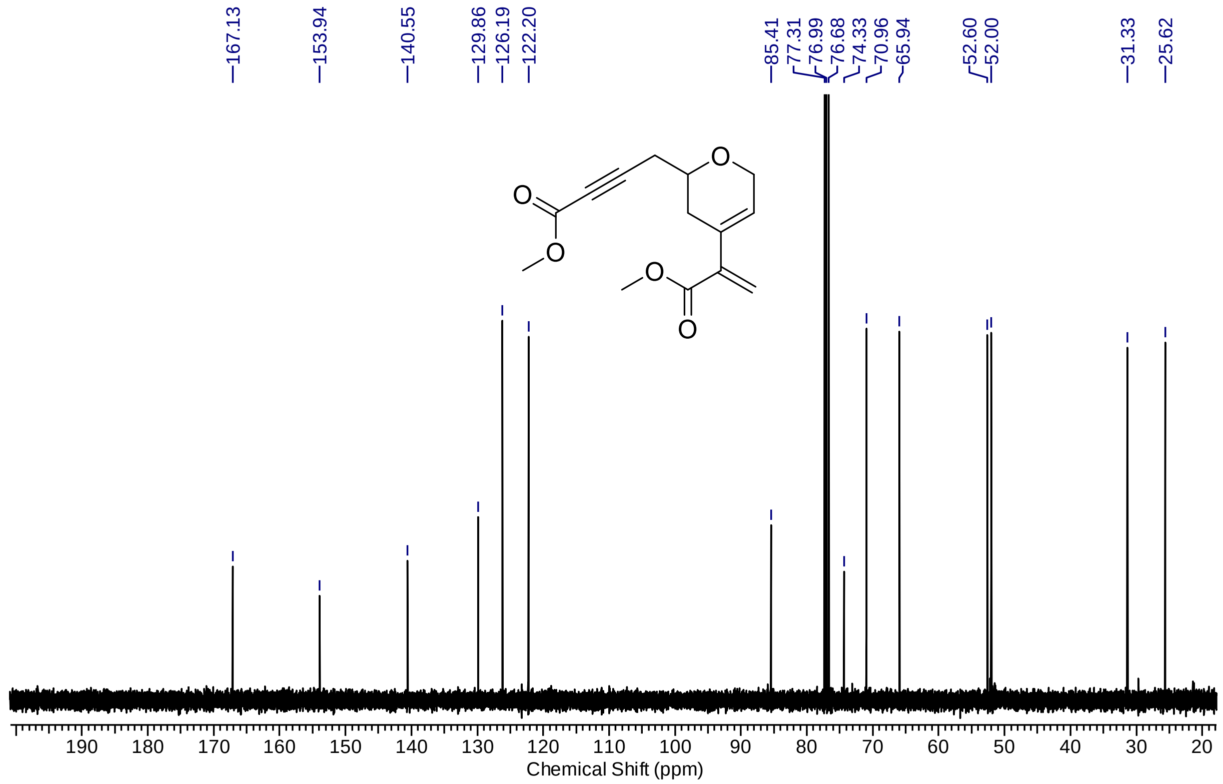

Figure S34. ¹³C{1H} NMR spectrum, 75.44 MHz, CDCl₃, methyl 4-(4-(3-methoxy-3-oxoprop-1-en-2-yl)-3,6-dihydro-2*H*-pyran-2-yl)but-2-ynoate (**12e**).

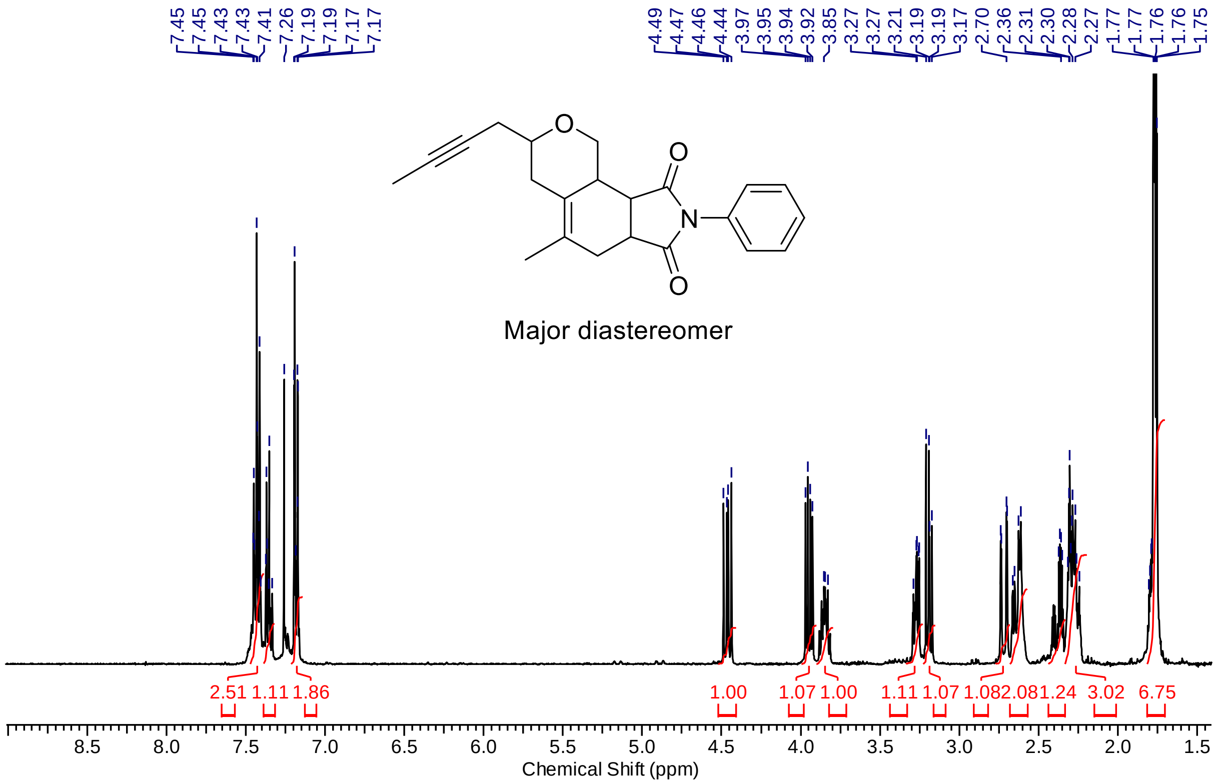

Figure S35. ^{1}H NMR spectrum, 299.97 MHz, $CDCl_3$, 3-(but-2-yn-1-yl)-5-methyl-8-phenyl-3,4,6,6a,9a,9b-hexahydropyrano[3,4-e]isoindole-7,9(1*H*,8*H*)-dione (**14**, major diastereoisomer).

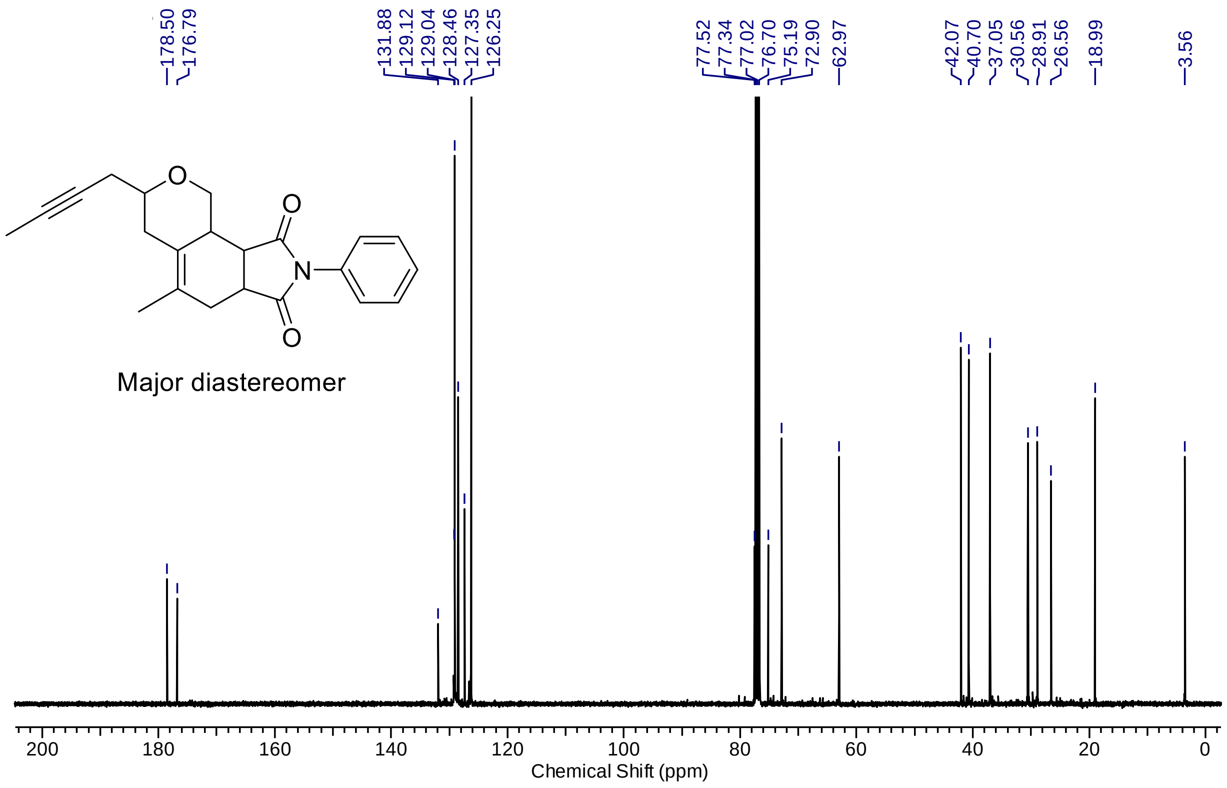

Figure S36. $^{13}C\{1H\}$ NMR spectrum, 75.44 MHz, $CDCl_3$, 3-(but-2-yn-1-yl)-5-methyl-8-phenyl-3,4,6,6a,9a,9b-hexahydropyrano[3,4-e]isoindole-7,9(1*H*,8*H*)-dione (**14**, major diastereoisomer).

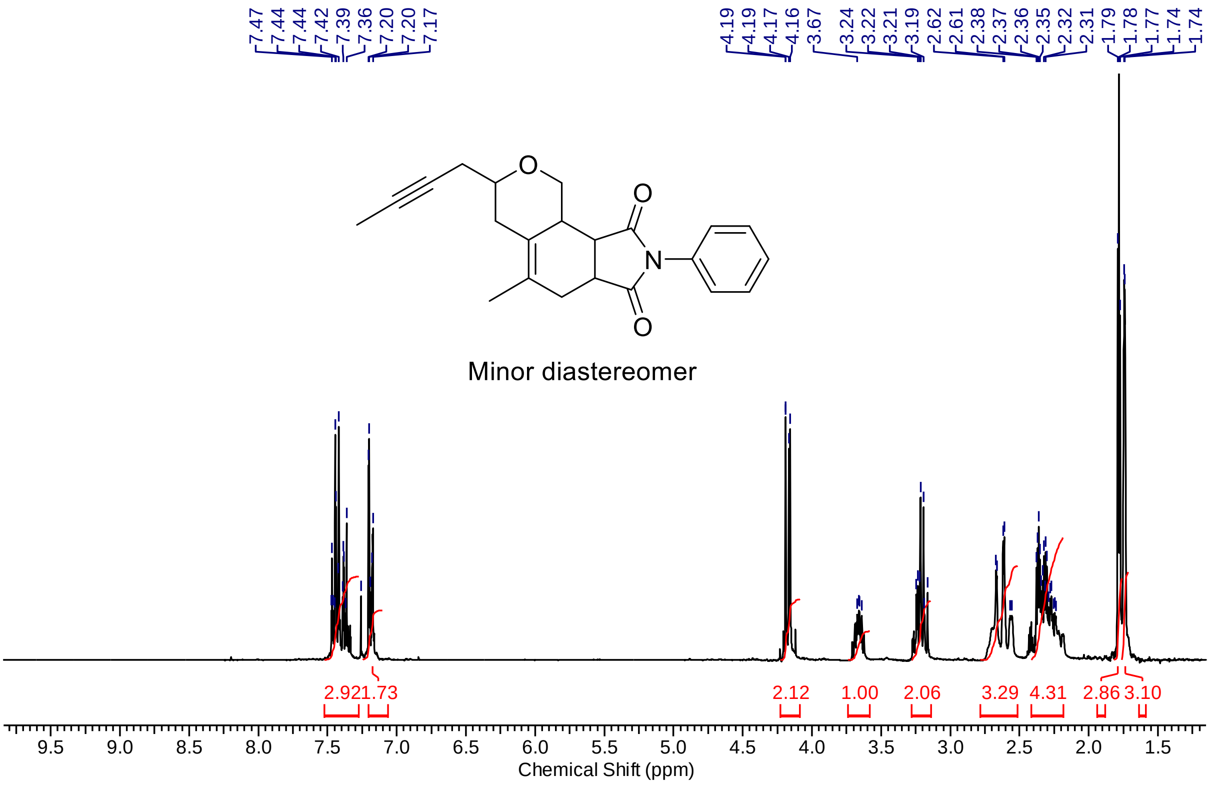

Figure S37. ^{1}H NMR spectrum, 299.97 MHz, $CDCl_3$, 3-(but-2-yn-1-yl)-5-methyl-8-phenyl-3,4,6,6a,9a,9b-hexahydropyrano[3,4-e]isoindole-7,9(1*H*,8*H*)-dione (**14**, minor diastereoisomer).

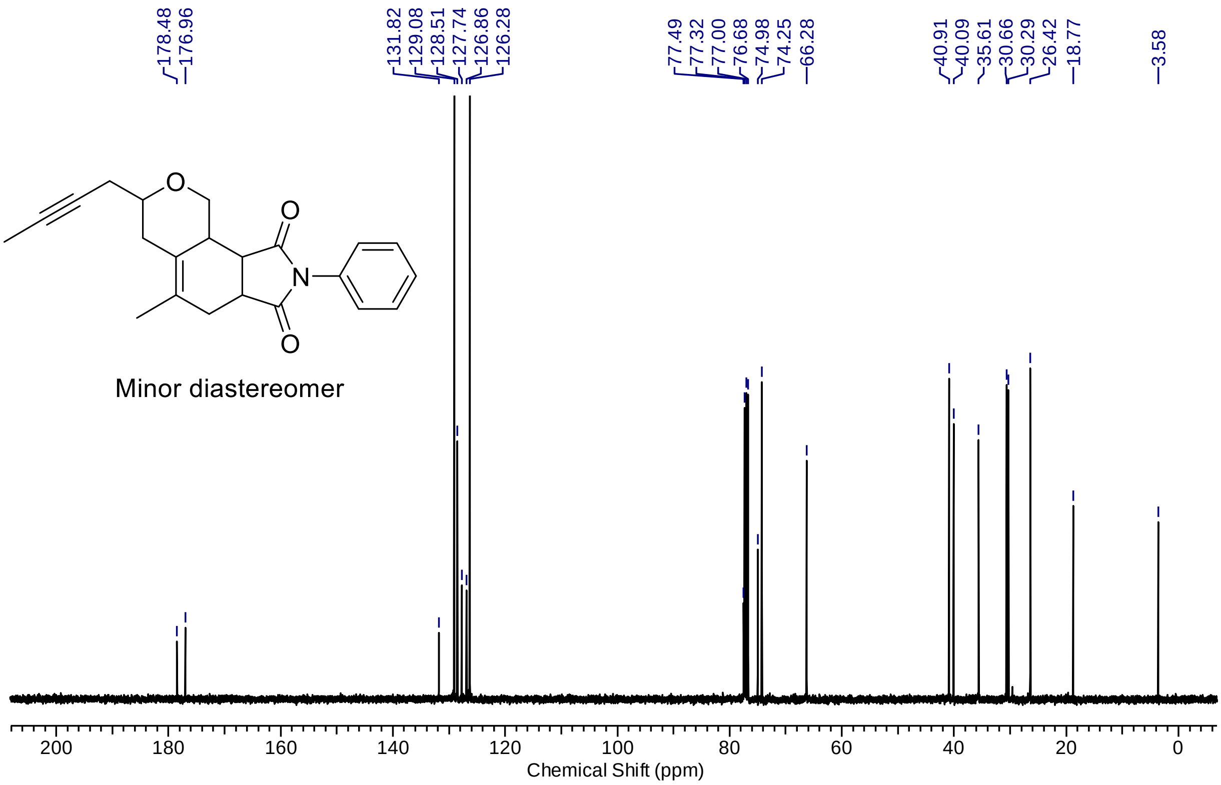

Figure S38. $^{13}C\{1H\}$ NMR spectrum, 75.44 MHz, $CDCl_3$, 3-(but-2-yn-1-yl)-5-methyl-8-phenyl-3,4,6,6a,9a,9b-hexahydropyrano[3,4-e]isoindole-7,9(1*H*,8*H*)-dione (**14**, minor diastereoisomer).

3. XYZ of all computed geometries (SMD(DCM)-RI-M06L/def2-SVP)

12b + 13, *endo-cis* complex

Gibbs free energy: -2969959.217 kJ/mol

C	-0.57768	-0.31897	1.62671
C	-0.49889	-1.63417	1.91955
C	-1.38480	-2.65302	1.29139
C	-2.68845	-0.92227	0.39258
C	-1.65526	0.15469	0.68642
H	-0.79902	-3.52919	0.95512
H	-3.36372	-1.02034	1.27250
H	-1.20964	0.51700	-0.26321
H	-2.17335	1.03272	1.11412
H	-2.11287	-3.05236	2.03749
O	-2.06694	-2.17518	0.15910
H	0.22198	-2.00070	2.66328
C	0.34512	0.69042	2.19922
C	1.58970	0.37195	2.60495
H	2.25660	1.13246	3.03429
H	1.99968	-0.64054	2.50298
C	-0.14119	2.10692	2.27409
H	-0.36907	2.51436	1.27083
H	-1.07509	2.19569	2.86161
H	0.61324	2.76783	2.73307
C	-3.52433	-0.62884	-0.84909
H	-4.21082	-1.48083	-1.01421
H	-2.84430	-0.61277	-1.72464
C	-4.27205	0.61622	-0.77137
C	-4.88837	1.66299	-0.69569
C	-5.62002	2.91118	-0.60861
H	-5.03747	3.75627	-1.01821
H	-6.57065	2.86837	-1.17020
H	-5.87160	3.16452	0.43717
C	2.10224	-1.90746	-0.29258
C	1.19123	-2.96877	-0.78789
C	0.33674	-2.43234	-1.66582
C	0.65175	-0.98778	-1.82109
H	1.26318	-3.99925	-0.43314
H	-0.47334	-2.90754	-2.22213
O	2.98015	-2.01226	0.53223
O	0.10617	-0.18451	-2.54301
N	1.73956	-0.72314	-0.96754
C	2.37121	0.53391	-0.80423
C	1.61066	1.70986	-0.82298
C	3.75498	0.60517	-0.59951
C	2.22962	2.94134	-0.62319
H	0.53176	1.66160	-0.98874
C	4.36077	1.84258	-0.38746
H	4.35741	-0.30630	-0.60044
C	3.60410	3.01490	-0.39481

H	1.62204	3.85405	-0.63431
H	5.44344	1.88674	-0.22116
H	4.08716	3.98482	-0.22938

12b + 13, *endo-trans* complex

Gibbs free energy: -2969956.083 kJ/mol

C	1.66431	1.37212	0.73828
C	2.39841	1.74359	-0.33764
C	1.87847	2.63942	-1.40417
C	-0.27254	2.36947	-0.51109
C	0.23139	1.83525	0.82057
H	2.60555	3.44803	-1.60893
H	-0.41726	1.51622	-1.20951
H	0.12699	2.61778	1.60118
H	-0.42860	1.00859	1.14294
H	1.77589	2.07262	-2.36035
O	0.66871	3.26920	-1.07258
H	3.44109	1.42335	-0.45064
C	2.19807	0.53635	1.83327
C	3.47133	0.08154	1.85964
H	3.82185	-0.55401	2.68333
H	4.21518	0.33996	1.09719
C	1.26427	0.17313	2.94826
H	0.82985	1.07045	3.42704
H	0.40695	-0.42724	2.59386
H	1.77724	-0.41231	3.72892
C	-1.57538	3.15213	-0.40258
H	-1.83089	3.52757	-1.41176
H	-1.37965	4.05318	0.21257
C	-2.69315	2.40412	0.15015
C	-3.64548	1.80845	0.61904
C	-4.79385	1.11975	1.17387
H	-5.54018	1.83457	1.56639
H	-5.30562	0.49798	0.41760
H	-4.51220	0.45473	2.01016
C	1.67074	-2.24701	0.23665
C	2.98170	-1.86599	-0.34521
C	2.77345	-1.10108	-1.42759
C	1.31695	-0.93521	-1.62683
H	3.92171	-2.21569	0.08694
H	3.50149	-0.65409	-2.10836
O	1.45635	-2.95504	1.19403
O	0.74952	-0.33516	-2.51347
N	0.68681	-1.64175	-0.57668
C	-0.70812	-1.86256	-0.46385
C	-1.50144	-1.98274	-1.61415
C	-1.30395	-1.98268	0.79951
C	-2.87161	-2.20789	-1.49462
H	-1.04520	-1.90862	-2.60353

C	-2.67316	-2.21960	0.90483
H	-0.69260	-1.91206	1.70167
C	-3.46545	-2.32929	-0.23808
H	-3.47871	-2.29945	-2.40277
H	-3.12416	-2.31656	1.89951
H	-4.54266	-2.51238	-0.15027

12b + 13, *exo-cis* complex

Gibbs free energy: -2969957.267 kJ/mol

C	1.40275	0.24960	-1.22815
C	0.95328	-0.91748	-1.73596
C	1.55186	-2.23316	-1.38458
C	3.36178	-1.09423	-0.41879
C	2.64031	0.24465	-0.37134
H	0.75504	-2.94365	-1.09445
H	3.88017	-1.18769	-1.39965
H	2.38107	0.50136	0.67870
H	3.33755	1.03760	-0.70061
H	2.06159	-2.68829	-2.26640
O	2.43923	-2.16440	-0.29257
H	0.09366	-0.93673	-2.41925
C	0.70481	1.53633	-1.44680
C	-0.62276	1.58794	-1.67824
H	-1.13006	2.54494	-1.86133
H	-1.24498	0.68324	-1.71198
C	1.51710	2.79264	-1.35137
H	1.93595	2.93197	-0.33662
H	2.38114	2.77805	-2.04246
H	0.91111	3.68396	-1.58357
C	4.37852	-1.27979	0.70147
H	4.78742	-2.30512	0.62559
H	3.83955	-1.24227	1.66943
C	5.45859	-0.30596	0.68596
C	6.36508	0.50582	0.66057
C	7.44627	1.47068	0.63587
H	7.35165	2.21485	1.44728
H	8.43080	0.98294	0.75376
H	7.47275	2.02997	-0.31677
C	-1.75112	0.82235	1.72110
C	-0.37887	0.31277	1.96987
C	-0.21796	-0.84467	1.31474
C	-1.47750	-1.16344	0.58896
H	0.33035	0.85159	2.60285
H	0.66229	-1.49187	1.25880
O	-2.26700	1.82787	2.15037
O	-1.71029	-2.11225	-0.12541
N	-2.37435	-0.12166	0.88153
C	-3.69692	-0.02492	0.38784
C	-4.52129	-1.15659	0.37188
C	-4.17870	1.19905	-0.09237

C	-5.81685	-1.06150	-0.13192
H	-4.14501	-2.10930	0.75534
C	-5.48160	1.28553	-0.57874
H	-3.53202	2.08087	-0.08399
C	-6.30339	0.15778	-0.60584
H	-6.45576	-1.95212	-0.14478
H	-5.85414	2.24752	-0.94938
H	-7.32538	0.22951	-0.99528

12b + 13, *exo-trans* complex

Gibbs free energy: -2969955.92 kJ/mol

C	1.40275	0.24960	-1.22815
C	0.95328	-0.91748	-1.73596
C	1.55186	-2.23316	-1.38458
C	3.36178	-1.09423	-0.41879
C	2.64031	0.24465	-0.37134
H	0.75504	-2.94365	-1.09445
H	3.88017	-1.18769	-1.39965
H	2.38107	0.50136	0.67870
H	3.33755	1.03760	-0.70061
H	2.06159	-2.68829	-2.26640
O	2.43923	-2.16440	-0.29257
H	0.09366	-0.93673	-2.41925
C	0.70481	1.53633	-1.44680
C	-0.62276	1.58794	-1.67824
H	-1.13006	2.54494	-1.86133
H	-1.24498	0.68324	-1.71198
C	1.51710	2.79264	-1.35137
H	1.93595	2.93197	-0.33662
H	2.38114	2.77805	-2.04246
H	0.91111	3.68396	-1.58357
C	4.37852	-1.27979	0.70147
H	4.78742	-2.30512	0.62559
H	3.83955	-1.24227	1.66943
C	5.45859	-0.30596	0.68596
C	6.36508	0.50582	0.66057
C	7.44627	1.47068	0.63587
H	7.35165	2.21485	1.44728
H	8.43080	0.98294	0.75376
H	7.47275	2.02997	-0.31677
C	-1.75112	0.82235	1.72110
C	-0.37887	0.31277	1.96987
C	-0.21796	-0.84467	1.31474
C	-1.47750	-1.16344	0.58896
H	0.33035	0.85159	2.60285
H	0.66229	-1.49187	1.25880
O	-2.26700	1.82787	2.15037
O	-1.71029	-2.11225	-0.12541
N	-2.37435	-0.12166	0.88153
C	-3.69692	-0.02492	0.38784

C	-4.52129	-1.15659	0.37188
C	-4.17870	1.19905	-0.09237
C	-5.81685	-1.06150	-0.13192
H	-4.14501	-2.10930	0.75534
C	-5.48160	1.28553	-0.57874
H	-3.53202	2.08087	-0.08399
C	-6.30339	0.15778	-0.60584
H	-6.45576	-1.95212	-0.14478
H	-5.85414	2.24752	-0.94938
H	-7.32538	0.22951	-0.99528

***endo-cis*-14A (*(3S,6aS,9aR,9bS)-14*)**

Gibbs free energy: -2970096.053 kJ/mol

C	-1.20326	-1.19442	0.41326
C	-0.80565	-1.88743	-0.87152
C	-1.54898	-1.26915	-2.04597
C	-3.33884	-0.45868	-0.67842
C	-2.17475	-0.06986	0.23831
H	-1.19864	-0.24232	-2.26629
H	-4.06155	-1.06483	-0.09752
H	-1.64783	0.79819	-0.21370
H	-2.57745	0.29127	1.19859
H	-1.41002	-1.85993	-2.96720
O	-2.93389	-1.28629	-1.76621
H	-1.14533	-2.94303	-0.81763
C	-0.63913	-1.63225	1.55905
C	0.35786	-2.75511	1.41956
H	0.90186	-2.93153	2.36362
H	-0.15625	-3.70542	1.17297
C	-0.87583	-1.09098	2.92749
H	0.05833	-0.67338	3.35325
H	-1.64080	-0.29813	2.96125
H	-1.18950	-1.89250	3.62434
C	-4.07406	0.76004	-1.25002
H	-4.84270	0.38932	-1.95492
H	-3.35816	1.34415	-1.86291
C	-4.68863	1.60878	-0.24162
C	-5.20433	2.29493	0.62192
C	-5.82241	3.11796	1.64259
H	-6.56183	3.81681	1.21116
H	-6.35359	2.50653	2.39424
H	-5.07748	3.72715	2.18579
C	1.37418	-0.62738	-1.28201
C	0.72942	-1.96753	-0.99833
C	1.37067	-2.45396	0.30659
C	2.29737	-1.33712	0.73685
H	0.97839	-2.62410	-1.85169
H	2.00067	-3.34594	0.13702
O	1.19192	0.08926	-2.23971
O	2.95376	-1.28276	1.75057

N	2.25971	-0.33430	-0.23830
C	3.03584	0.85427	-0.16997
C	4.41373	0.76526	0.04807
C	2.42189	2.10160	-0.31334
C	5.17636	1.92906	0.12320
H	4.88519	-0.21679	0.15479
C	3.19482	3.25986	-0.24530
H	1.34040	2.16281	-0.47059
C	4.57072	3.17776	-0.02567
H	6.25631	1.85733	0.29596
H	2.71214	4.23741	-0.35874
H	5.17349	4.09145	0.03071

***endo-trans*-14B (*(3S,6aR,9aS,9bR)-14*)**
Gibbs free energy: -2970099.658 kJ/mol

C	-1.20478	-1.80903	-0.03738
C	-0.51110	-2.08340	1.28543
C	-0.99974	-1.07892	2.32149
C	-1.87932	0.46118	0.71771
C	-2.34316	-0.84110	0.06202
H	-2.02292	-1.33299	2.66455
H	-1.31697	1.05373	-0.03112
H	-3.16171	-1.27792	0.67161
H	-2.78605	-0.60967	-0.91916
H	-0.35327	-1.06308	3.21345
O	-0.95713	0.22661	1.78033
H	-0.79668	-3.09959	1.62675
C	-0.77677	-2.42764	-1.15836
C	0.45908	-3.27943	-1.04115
H	0.86914	-3.51270	-2.03911
H	0.24288	-4.25272	-0.55681
C	-1.36995	-2.29764	-2.51962
H	-2.28893	-1.69078	-2.55310
H	-0.63958	-1.84882	-3.22125
H	-1.61481	-3.29346	-2.93769
C	-3.03586	1.30885	1.25869
H	-2.60331	2.20030	1.75198
H	-3.54268	0.73517	2.06110
C	-3.99771	1.71409	0.24601
C	-4.78866	2.04744	-0.61753
C	-5.73496	2.44755	-1.64005
H	-6.52981	1.69303	-1.78201
H	-6.23279	3.40078	-1.38537
H	-5.24267	2.58980	-2.61913
C	1.88511	-1.23086	-0.90053
C	1.50525	-2.53486	-0.22257
C	1.04522	-2.11294	1.17107
C	1.67133	-0.75033	1.36371
H	2.43463	-3.13197	-0.14846
H	1.43094	-2.76970	1.96754

O	2.07527	-1.05212	-2.08188
O	1.90222	-0.18004	2.40306
N	1.99773	-0.24948	0.09032
C	2.42260	1.08464	-0.13778
C	1.75537	2.13210	0.50851
C	3.49099	1.35323	-0.99928
C	2.16278	3.44604	0.28993
H	0.91823	1.90507	1.17680
C	3.88321	2.67306	-1.21729
H	4.01319	0.53043	-1.49534
C	3.22424	3.72175	-0.57409
H	1.63894	4.26434	0.79738
H	4.71942	2.88145	-1.89469
H	3.53890	4.75760	-0.74598

***exo-cis*-14C (*(3S,6aR,9aS,9bS)-14*)**

Gibbs free energy: -2970091.474 kJ/mol

C	-1.79110	0.73608	0.31920
C	-0.69001	-0.30721	0.40276
C	-1.21287	-1.73980	0.30823
C	-3.44273	-1.17867	0.02905
C	-3.21290	0.32633	0.02128
H	-0.44289	-2.41656	-0.09247
H	-3.56329	-1.52974	1.07925
H	-3.49170	0.71031	-0.98210
H	-3.92159	0.80420	0.72309
H	-1.48258	-2.10600	1.32219
O	-2.33331	-1.85158	-0.53578
H	-0.18422	-0.20576	1.38513
C	-1.40644	2.02940	0.38192
C	0.06771	2.29982	0.47991
H	0.29566	3.37295	0.36676
H	0.45699	2.00713	1.47676
C	-2.31627	3.20484	0.26839
H	-2.11253	3.77893	-0.65780
H	-3.38528	2.93732	0.26154
H	-2.15475	3.91766	1.10006
C	-4.66923	-1.60102	-0.77254
H	-4.73120	-2.70568	-0.75374
H	-4.50012	-1.32645	-1.83291
C	-5.91154	-1.01764	-0.29170
C	-6.94556	-0.52667	0.12208
C	-8.17976	0.05670	0.60890
H	-8.03118	0.58469	1.56824
H	-8.59619	0.79009	-0.10516
H	-8.95638	-0.71116	0.77772
C	2.29800	1.47930	-0.33210
C	0.81476	1.49626	-0.59809
C	0.38511	0.02803	-0.66586
C	1.66362	-0.76119	-0.48473

H	0.66080	1.99840	-1.57057
H	-0.03195	-0.23828	-1.65444
O	3.02020	2.43362	-0.16167
O	1.80445	-1.96302	-0.50901
N	2.71516	0.14295	-0.27389
C	4.05543	-0.25348	-0.01817
C	4.31827	-1.24154	0.93650
C	5.10716	0.35195	-0.71327
C	5.63411	-1.62451	1.18854
H	3.49166	-1.70749	1.48087
C	6.42045	-0.03052	-0.44531
H	4.89696	1.11633	-1.46671
C	6.68826	-1.01857	0.50317
H	5.83504	-2.40102	1.93550
H	7.24261	0.44831	-0.98944
H	7.72244	-1.31829	0.70816

***exo-trans*-14D (*(3S,6aS,9aR,9bR)-14*)**

Gibbs free energy: -2970098.673 kJ/mol

C	1.77907	0.68999	0.48522
C	0.71576	-0.39105	0.46967
C	1.37180	-1.74255	0.20099
C	3.42702	-0.84314	-0.60131
C	3.18081	0.16773	0.52378
H	1.88217	-2.12056	1.11104
H	3.63561	-0.29145	-1.53799
H	3.35976	-0.33335	1.49837
H	3.93170	0.97074	0.45611
H	0.62502	-2.49794	-0.08871
O	2.27312	-1.63153	-0.88031
H	0.21969	-0.43015	1.46352
C	1.39379	1.98280	0.47224
C	-0.08712	2.25142	0.43084
H	-0.30066	3.31807	0.24732
H	-0.54442	2.01242	1.41388
C	2.29018	3.17406	0.50610
H	3.36307	2.92431	0.52160
H	2.10869	3.83030	-0.36711
H	2.08305	3.79935	1.39653
C	4.60474	-1.78254	-0.31056
H	4.68396	-2.50054	-1.14894
H	4.35585	-2.38790	0.58436
C	5.87159	-1.09623	-0.11338
C	6.92015	-0.49776	0.04419
C	8.17334	0.20635	0.23131
H	9.04200	-0.45332	0.05450
H	8.26947	1.06151	-0.46187
H	8.27088	0.60588	1.25703
C	-2.26493	1.42375	-0.50112
C	-0.76376	1.39938	-0.65117

C	-0.36549	-0.07502	-0.58688
C	-1.65224	-0.82154	-0.32127
H	-0.51872	1.83706	-1.63515
H	0.02307	-0.42192	-1.56254
O	-2.98711	2.39277	-0.51167
O	-1.79386	-2.01069	-0.14755
N	-2.70120	0.10794	-0.29358
C	-4.05553	-0.23974	-0.04022
C	-4.37368	-1.01351	1.08021
C	-5.06366	0.20436	-0.90066
C	-5.70305	-1.34539	1.33406
H	-3.57963	-1.34750	1.75506
C	-6.39137	-0.12566	-0.63323
H	-4.80557	0.80509	-1.77830
C	-6.71464	-0.90133	0.48134
H	-5.94997	-1.95202	2.21294
H	-7.18067	0.22582	-1.30758
H	-7.75978	-1.16005	0.68635

***endo-cis*-15A (TS 12b + 13 to 14A)**

Gibbs free energy: -2969894.554 kJ/mol

One imaginary frequency detected

C	-2.13171	-0.04501	0.82617
C	-2.92082	-0.21455	-0.28334
C	-3.31562	0.93387	-1.13884
C	-2.02517	2.42821	0.18329
C	-1.65733	1.33719	1.19517
H	-2.70098	0.95282	-2.07016
H	-2.20783	3.36944	0.72905
H	-0.56285	1.34501	1.35787
H	-2.08764	1.58979	2.18390
H	-4.36133	0.82087	-1.47542
O	-3.25864	2.16402	-0.45837
H	-3.40673	-1.16914	-0.48893
C	-1.72085	-1.16953	1.61535
C	-2.14431	-2.46071	1.27820
H	-1.83373	-3.28251	1.93661
H	-3.13358	-2.60494	0.82960
C	-0.68856	-0.97519	2.67733
H	-0.45070	-1.91751	3.19610
H	0.25761	-0.56116	2.27509
H	-1.03218	-0.24770	3.43766
C	-0.91727	2.70630	-0.84858
H	-1.35567	3.34054	-1.64331
H	-0.60298	1.76704	-1.34901
C	0.22935	3.37736	-0.25823
C	1.15645	3.95301	0.28186
C	2.26844	4.63818	0.91119
H	2.68336	4.05705	1.75467
H	3.09454	4.81672	0.19926

H	1.96713	5.62319	1.31208
C	-0.26986	-1.23379	-1.57792
C	-1.38484	-2.15187	-1.48027
C	-1.18347	-3.01787	-0.40695
C	0.21143	-2.79195	0.08302
H	-2.18135	-2.18186	-2.22547
H	-1.59795	-4.02975	-0.35366
O	-0.05706	-0.31744	-2.35189
O	0.83444	-3.43708	0.89847
N	0.65788	-1.63561	-0.55917
C	1.94053	-1.06842	-0.38296
C	3.07838	-1.88450	-0.34596
C	2.07406	0.31809	-0.25622
C	4.33697	-1.31045	-0.17557
H	2.97482	-2.96741	-0.45737
C	3.33784	0.88478	-0.10174
H	1.18219	0.95156	-0.27649
C	4.47291	0.07380	-0.05688
H	5.22306	-1.95530	-0.14726
H	3.43303	1.97322	-0.01371
H	5.46539	0.52178	0.06881

***endo-trans*-15B (TS 12b + 13 to 14B)**

Gibbs free energy: -2969909.821 kJ/mol

One imaginary frequency detected

C	1.65942	1.27923	0.83545
C	2.39027	1.70431	-0.24744
C	1.83881	2.63059	-1.26820
C	-0.29519	2.29239	-0.35120
C	0.23503	1.75832	0.97003
H	2.54935	3.45598	-1.46025
H	-0.41589	1.44206	-1.05880
H	0.16732	2.54963	1.74499
H	-0.42306	0.94030	1.31614
H	1.73033	2.08277	-2.23493
O	0.62060	3.22503	-0.90283
H	3.45490	1.47398	-0.33743
C	2.17757	0.31036	1.75551
C	3.37398	-0.35743	1.47939
H	3.72541	-1.09685	2.21137
H	4.18210	0.14412	0.93690
C	1.33937	-0.13163	2.90944
H	1.05772	0.72979	3.54456
H	0.38368	-0.59167	2.59636
H	1.86830	-0.86165	3.54225
C	-1.62075	3.03322	-0.22968
H	-1.88132	3.42479	-1.23141
H	-1.45747	3.92463	0.40833
C	-2.71811	2.23632	0.29546
C	-3.65340	1.59524	0.73828

C	-4.78252	0.85816	1.27041
H	-5.48424	1.52609	1.80279
H	-5.35723	0.35330	0.47283
H	-4.46602	0.08170	1.99003
C	1.64420	-2.10583	0.22764
C	2.98058	-1.54800	-0.14501
C	2.80016	-0.79344	-1.29737
C	1.39590	-0.71810	-1.62128
H	3.87176	-2.14387	0.07493
H	3.56727	-0.37101	-1.94928
O	1.39734	-2.92960	1.08218
O	0.84172	-0.15982	-2.55297
N	0.70899	-1.48196	-0.61180
C	-0.68727	-1.70878	-0.57031
C	-1.45095	-1.73216	-1.74815
C	-1.32515	-1.92678	0.66116
C	-2.82643	-1.94930	-1.68321
H	-0.96772	-1.58481	-2.71514
C	-2.69708	-2.16093	0.70925
H	-0.74179	-1.93562	1.58418
C	-3.45860	-2.16749	-0.45967
H	-3.40776	-1.96154	-2.61284
H	-3.17565	-2.33638	1.68019
H	-4.53925	-2.34664	-0.41728

***exo-cis*-15C (TS 12b + 13 to 14C)**
Gibbs free energy: -2969900.209 kJ/mol
One imaginary frequency detected

C	1.66258	0.52882	-0.51091
C	0.78143	-0.53167	-0.55057
C	1.18207	-1.90768	-0.16523
C	3.41167	-1.21440	-0.05652
C	3.07159	0.26673	-0.04928
H	0.39408	-2.37016	0.45961
H	3.51061	-1.55869	-1.11080
H	3.21245	0.66849	0.97635
H	3.79163	0.81693	-0.68319
H	1.25730	-2.54415	-1.07963
O	2.37590	-1.95423	0.57015
H	-0.18781	-0.44359	-1.05356
C	1.21653	1.87320	-0.67846
C	-0.14388	2.14105	-0.82227
H	-0.47204	3.18424	-0.91707
H	-0.79123	1.42190	-1.33853
C	2.16584	2.99323	-0.40242
H	2.58367	2.93746	0.62226
H	3.03731	2.96842	-1.08314
H	1.68129	3.97647	-0.51636
C	4.69287	-1.54716	0.69891
H	4.82557	-2.64555	0.68452

H	4.54528	-1.27620	1.76325
C	5.87629	-0.88897	0.16864
C	6.86142	-0.33478	-0.28276
C	8.03927	0.32232	-0.81367
H	7.78421	1.01551	-1.63550
H	8.56173	0.91264	-0.03939
H	8.76715	-0.40548	-1.21594
C	-2.35848	1.61683	0.60078
C	-0.93583	1.64272	1.05523
C	-0.59542	0.34200	1.41970
C	-1.69415	-0.55134	1.09603
H	-0.55036	2.54618	1.53632
H	0.24619	0.01652	2.03397
O	-3.06411	2.55492	0.30133
O	-1.79604	-1.75406	1.25260
N	-2.72275	0.26500	0.53482
C	-3.93755	-0.22723	0.00960
C	-3.92895	-1.36093	-0.81450
C	-5.14949	0.41292	0.29875
C	-5.12474	-1.85190	-1.33431
H	-2.98130	-1.85667	-1.04621
C	-6.33770	-0.07695	-0.24132
H	-5.15828	1.29117	0.94970
C	-6.33258	-1.21003	-1.05590
H	-5.10885	-2.74198	-1.97427
H	-7.28131	0.43296	-0.01383
H	-7.27070	-1.59509	-1.47198

***exo-trans*-15D (TS 12b + 13 to 14D)**
Gibbs free energy: -2969880.25 kJ/mol
One imaginary frequency detected

C	1.62290	0.35465	1.10791
C	0.68670	-0.65782	1.00000
C	1.17152	-2.03222	0.71920
C	3.19468	-1.05959	-0.21653
C	3.04319	-0.11841	0.97391
H	1.51398	-2.50773	1.66933
H	3.08769	-0.46067	-1.14630
H	3.33425	-0.66831	1.89312
H	3.76330	0.70747	0.86553
H	0.35642	-2.66754	0.33237
O	2.20038	-2.07625	-0.24970
H	-0.34319	-0.54684	1.35593
C	1.25673	1.72736	1.12675
C	-0.08987	2.07102	1.03049
H	-0.37347	3.13108	0.99969
H	-0.85571	1.41231	1.45675
C	2.30397	2.78636	0.97281
H	3.06809	2.72984	1.76911
H	2.85062	2.69054	0.01404

H	1.86559	3.79695	1.00544
C	4.55428	-1.76326	-0.25053
H	4.53578	-2.48996	-1.08482
H	4.65634	-2.36722	0.67292
C	5.68568	-0.86126	-0.39535
C	6.62105	-0.09108	-0.51367
C	7.74356	0.81553	-0.65439
H	8.70792	0.27836	-0.60335
H	7.71939	1.35136	-1.62050
H	7.75442	1.58036	0.14306
C	-2.09128	1.55781	-0.72442
C	-0.62363	1.43982	-0.95228
C	-0.33296	0.08871	-1.12240
C	-1.54686	-0.69166	-0.89589
H	-0.08832	2.26506	-1.42902
H	0.53829	-0.35392	-1.60763
O	-2.76216	2.56259	-0.63540
O	-1.72116	-1.89313	-0.95169
N	-2.57048	0.24526	-0.58607
C	-3.88880	-0.10731	-0.21999
C	-4.09514	-1.10174	0.74490
C	-4.98694	0.52694	-0.81343
C	-5.39160	-1.46039	1.10738
H	-3.23447	-1.59010	1.21328
C	-6.28007	0.17099	-0.43248
H	-4.82534	1.29546	-1.57447
C	-6.48843	-0.82398	0.52379
H	-5.54376	-2.24100	1.86193
H	-7.13554	0.67342	-0.89905
H	-7.50741	-1.10410	0.81475

References

1. Nicolaou, K. C.; Skokotas, G.; Furaya, S.; Suemune, H.; Nicolaou, D. C., *Angew. Chem.* **1990,** *102*, 1066-1068.
